# The Association between High Fat Diet around Gestation and Metabolic Syndrome-related Phenotypes in Rats: A Systematic Review and Meta-Analysis

**DOI:** 10.1038/s41598-017-05344-7

**Published:** 2017-07-11

**Authors:** Mariana L. Tellechea, Melisa F. Mensegue, Carlos J. Pirola

**Affiliations:** 1 University of Buenos Aires, Institute of Medical Research A Lanari, Buenos Aires, Argentina; 2 National Scientific and Technical Research Council (CONICET) - University of Buenos Aires, Institute of Medical Research (IDIM), Department of Molecular Genetics and Biology of Complex Diseases, Buenos Aires, Argentina

## Abstract

Numerous rodent studies have evaluated the effects of a maternal high-fat diet (HFD) on later in life susceptibility to Metabolic Syndrome (MetS) with varying results. Our aim was to quantitatively synthesize the available data on effects of maternal HFD around gestation on offspring’s body mass, body fat, plasma leptin, glucose, insulin, lipids and systolic blood pressure (SBP). Literature was screened and summary estimates of the effect of maternal HFD on outcomes were calculated by using fixed- or random-effects models. 362 effect sizes from 68 studies together with relevant moderators were collected. We found that maternal HFD is statistically associated with higher body fat, body weight, leptin, glucose, insulin and triglycerides levels, together with increased SBP in offspring later in life. Our analysis also revealed non-significant overall effect on offspring’s HDL-cholesterol. A main source of variation among studies emerged from rat strain and lard-based diet type. Strain and sex -specific effects on particular data subsets were detected. Recommendations are suggested for future research in the field of developmental programming of the MetS. Despite significant heterogeneity, our meta-analysis confirms that maternal HFD had long-term metabolic effects in offspring.

## Introduction

Metabolic syndrome (MetS) is defined as a cluster of important risk factors including central obesity, high fasting plasma glucose or glucose intolerance, low high density lipoprotein cholesterol (HDL-c), high triglycerides, and elevated blood pressure, which are multiple metabolic risk factors for diabetes and cardiovascular morbimortality^1, 2^. MetS is a common multifactorial disease with rising prevalence worldwide, which relates largely to increasing obesity caused by western diet and sedentary lifestyles. MetS is considered a consequence of a complex interplay between genetic and environmental factors, and according to the “developmental origins of health and disease” hypothesis, the MetS can also be considered as a developmental process that can be modified by changes in the environment early in life. The “developmental origins of health and disease” hypothesis, also called developmental programming, can be defined as the response to a specific challenge during a critical developmental time period that changes the trajectory of development with resulting effects on health that linger throughout life^3^. The fetal origins of obesity, insulin resistance and cardiovascular disease have been investigated in a broad range of epidemiological and animal studies.

The late onset of such diseases in response to earlier transient experiences has led to the suggestion that developmental programming may have an epigenetic component, as epigenetic marks such as DNA methylation or covalent posttranslational histone modifications, both involving chromatin remodeling, could provide a persistent remembrance of earlier nutritional states^4–6^. A growing body of evidence supports the notion that epigenetic changes contribute to fetal metabolic programming^4–8^, however the mechanisms by which early environmental insults may have long-term effects on offspring are relatively unclear. To date, these mechanisms include changes in gene expression caused by epigenetic modifications, permanent changes in cellular composition and ageing, and permanent structural changes to the organ^4, 6^.

Whilst the programming of obesity is undoubtedly a multifactorial process, the diversity of models with a common end-point might suggest some common pathways^9, 10^. Several studies conducted in different animal species have shown that maternal high fat diet (HFD) consumption leads to metabolic abnormalities in offspring during adult lifetime such as increased body weight and fat mass, reduced insulin sensitivity, increased blood glucose and triglycerides levels, increased lipid deposition, vascular endothelial cell dysfunction and increased serum leptin levels^3, 11, 12^. Altogether this evidence suggest that consumption of a HFD by female rats results in an adverse maternal intrauterine environment predisposing the offspring to a MetS-like phenotype later in life.

The maternal HFD-induced phenotype varies distinctly among different studies because this intervention is not standardized. Diets with dissimilar fatty acid compositions are designated under the term HFD in bibliography. And it is not only the quantity of fat but also the type of fat that influences offspring phenotype. Furthermore, variation in diet macronutrient composition may explain differences among the results reported. However, diverse experimental results seem not likely explained only by the characteristics of the diet. There are also many other likely sources of heterogeneity among the results, including the diversity of experimental designs. How much intervention is needed? How long should intervention continue? Answers to these questions are needed. If the intervention time and duration is inappropriate, researchers may construct an unsuccessful model, with all the implications that this has for the advancement of science and for animal welfare. It also remains to be tested which biological factors, such as strain, offspring sex and age at measurement, should be taken into account when designing experimental protocols.

Our main aim was to quantify the overall effect of maternal HFD consumption on developmental programming of offspring’s metabolism. We collected the vast experimental data available on rats for the long-term effects of maternal HFD on MetS-related phenotypes: (1) body fat (adiposity was also estimated indirectly by body weight and plasma leptin concentration), (2) plasma fasting glucose and insulin concentrations, (3) HDL-c, (4) plasma triglycerides, and (5) systolic blood pressure (SBP). We selected studies in which offspring were given a standard diet after weaning. We assessed the long-term programming effects collecting data on phenotypes at different time points after weaning. Then, using meta-regression, we evaluated the influence of biological (offspring’s age and sex) and experimental factors (duration of maternal dietary manipulation, litter size and experimental diet macronutrient composition). Finally, we also reviewed the influence of additional moderators: maternal weight and birthweight. We predict that the above-mentioned moderators may account for the ambiguous results from different experimental studies. As far as possible, we had try to establish whether there is an ideal maternal HFD protocol to model MetS in rat offspring.

This meta-analysis is not intended to throw light on the subjacent mechanisms at the gene or cellular level. However, since mechanisms by which maternal dietary imbalance affects fetal and postnatal development remain poorly understood, we believe that this study will be a good start point for future maternal HFD experiments.

## Results

### Study characteristics

The characteristics of the selected studies are shown in Table 1. The experimental rat strains were predominantly Sprague Dawley and Wistar, and outcomes were reported either for males, females or mixed-sex groups. We extracted 362 effect sizes from 68 studies. Summary information for each data subset is presented in Supplementary Table S1. The number of data points (effect sizes) within each outcome ranged from 14 to 75 and the number of studies these data points were derived from ranged from 9 to 49. In experiments where different groups were subjected to different diet exposure or composition, we considered the groups to be independent. In 3 studies, male genitor rats were also provided with the same experimental diet as the female rats^13–15^. Data on timing of maternal dietary manipulations for each outcome are presented in Supplementary Fig. S1. The duration of the interventions ranged from 9 (gestation only) to 154 (inclusive of a pre-mating period, gestation and lactation) days. Dam nutritional manipulation was ceased at birth in 7.3% cases meanwhile in the remaining cases exposure extended into lactation.

**Table 1:** Characteristics of the selected studies.

Reference	Strain	Sex	Main fat source	Duration of Intervention (days)	Age stage	Outcomes
Burgueño A.L.^26^ ^a,b^	W	M, F	lard	57	A	FAT, BW, LEP, GLU, INS, TG, HDL
Srinivasan M.^43^ ^a^	SD	M	lard	141	PPP	INS
Srinivasan M.^43^ ^b^	SD	M	lard	141	YA	BW, GLU, INS, TG
Tamashiro K.L.^47^ ^a^	SD	M	lard	40	YA	FAT, BW, LEP, GLU, INS
Tamashiro K.L.^47^ ^b^	SD	F	lard	40	YA	FAT, BW
Sun B.^48^ ^a,b^	SD	M, F	lard	40	YA	FAT
Sun B.^48^ ^c,d^	SD	M, F	lard	19	YA	FAT
Sun B.^49^ ^a^	SD	M	lard	40	PPP	LEP
Sun B.^49^ ^b^	SD	M	lard	40	YA	FAT, LEP
White C.L., Bruce-Keller A.J.^50^	LE	M	lard	70	PPP	BW
White C.L., Morrison C.D.^17^ ^a^	LE	M	lard	70	PPP	FAT, GLU
White C.L., Morrison C.D.^17^ ^b^	LE	M	lard (food availability set)	70	PPP	FAT, GLU
Sasaki A.^51^ ^a,b^	LE	M, F	lard	70	YA	BW
Marco A.^[Bibr CR52][Bibr CR52]^	W	F	lard	100	YA	BW, LEP
Lecoutre S.^53^ ^a,b^	W	M, F	lard	154	A	FAT, BW, LEP, GLU, INS, TG
Ambrosetti V.^54^ ^a^	SD	F	lard	*na*	PPP	BW, INS
Ambrosetti V.^54^ ^b^	SD	F	lard	*na*	YA	BW
Guberman C.^55^ ^a,b^	SD	M	lard	98, 77	YA	BW, SBP
Seet E.L.^56^	SD	M	lard	98	YA	TG
Desai M.^57^ ^a,c^	SD	M, F	lard	98	PPP	SBP
Desai M.^57^ ^b,d^	SD	M, F	lard	98	YA	FAT, BW, LEP, GLU, INS, TG
Desai M.^57^ ^e,g^	SD	M, F	lard	77	PPP	SBP
Desai M.^57^ ^f,h^	SD	M, F	lard	77	YA	FAT, BW, LEP, GLU, INS, TG
Desai M.^58^	SD	M	lard	98	A	FAT, BW, GLU, INS, TG
Walker C.D.^59^ ^a^	SD	M	lard	28	PPP	FAT, INS
Walker C.D.^59^ ^b^	SD	M	lard	28	YA	FAT, BW, LEP
Naef L.^60^	SD	M	lard	28	YA	FAT, BW
Koukkou E.^61^	SD	*na*	lard	47	PPP	TG
Mendes-da-Silva C.^62^	W	both	lard	21	YA	BW
Taylor P.D.^63^	SD	F	lard	52	*na*	LEP
Khan I.Y.^21^ ^a,b^	SD	M	lard	52	YA, A	GLU, INS, HDL, TG
Khan I.Y.^21^ ^c^	SD	F	lard	52	YA	GLU, INS, HDL, TG, SBP
Khan I.Y.^20^ ^d^	SD	F	lard	52	A	BW, GLU, INS, HDL, TG, SBP
Khan I.Y.^21^ ^a,d^	SD	M	lard	52, 31	YA	FAT, BW, GLU, HDL, TG
Khan I.Y.^21^ ^b,e^	SD	F	lard	52, 31	YA	FAT, BW, GLU, HDL, TG, SBP
Khan I.Y.^21^ ^c,f^	SD	both	lard	52, 31	YA	INS
Khan I.Y.^19^ ^a^	SD	M	lard	52	YA	BW, GLU, INS, HDL, TG
Khan I.Y.^19^ ^b^	SD	F	lard	52	YA	GLU, INS, HDL, TG, SBP
Armitage J.A.^64^ ^a,b^	SD	M, F	lard	52	YA	BW
Eleftheriades M.^65^	W	both	lard	9	A	BW, GLU, HDL, TG
Vega C.C.^66^ ^a,b^	W	M, F	lard	141	PPP	FAT, LEP, GLU, INS, TG
Bautista C.J.^67^ ^a,b^	W	M, F	lard	141	PPP	FAT, BW
Rodríguez-González G.L. 2015^a,b^ ^68^	W	M	lard	141	YA, A	FAT, BW
Zambrano E.^69^	W	M	lard	141	YA	FAT, BW, LEP, GLU, INS
Santos M.^70^	W	M	lard	141	A	FAT, BW
Zhang X.^71^	SD	M	lard	42	YA	TG
Page K.C.^72^	SD	M	lard	73	YA	FAT, BW, LEP, GLU, INS
Howie G.J.^44^ ^a,b^	W	M, F	lard	42	YA	FAT, LEP, GLU, INS
Howie G.J.^44^ ^c,d^	W	M, F	lard	140	YA	FAT, LEP, GLU, INS
Howie G.J.^73^ ^a,b^	W	M	lard	42, 140	YA	BW
Smith T.^74^	W	M	lard	42	YA	FAT, BW, LEP, GLU, INS, HDL
Pereira T.J.^75^ ^a^	SD	M	lard	84	YA	FAT, LEP, GLU, INS
Pereira T.J.^75^ ^b^	SD	F	lard	84	YA	FAT, GLU, INS
Cordero P.^76^ ^a,b^	W	M, F	lard	42	YA	FAT, BW
Sloboda D.M.^77^ ^a,b^	W	F	lard	42, 140	PPP	BW
Tsoulis M.W.^78^	W	F	lard	42	YA	BW, LEP, INS
Gray C., Reynolds C.M.^79^	SD	M	lard	52	YA	FAT, BW, HDL, TG, SBP
Reynolds C.M.^80^	SD	F	lard	52	YA	FAT, LEP, HDL, TG
Pileggi C.A.^81^	SD	M	lard	52	YA	BW
Song Y.^82^	SD	M	lard	105	YA	FAT, BW, LEP
Latouche C.^83^	SD	M	lard	63	A	FAT, BW, GLU, INS
Yang K.F.^84^	SD	F	lard	42	YA	GLU, INS, TG
Ghosh P.^85^	SD	F	lard	52	YA	BW, HDL, TG
Miotto P.M.^86^ ^a,b^	W	M, F	lard	110	YA	FAT, LEP
MacPherson R.E.^87^	W	both	lard	110	YA	FAT, BW
Hanafi M.Y.^88^ ^a,c^	W	M, F	lard	*na*	YA	LEP
Hanafi M.Y.^88^ ^b,d^	W	M, F	lard	*na*	A	LEP, GLU, INS, HDL, TG
Mazzucco M.B.^89^ ^a,b^	W	M, F	butter	98	YA	BW, GLU, TG
Kozak R.^90^	LE	M	margarine	*na*	YA	BW, GLU, INS
Adamu H.A.^16^	SD	M	corn oil + cream milk	49	PPP	BW, LEP, INS
Trottier G.^38, 91^	SD	both	corn oil + cream milk	26.5	PPP	FAT, LEP
Couvreur O.^92^ ^a,b^	W	M, F	palm oil	89	YA	BW, LEP, GLU, INS, TG
Férézou-Viala J.^93^ ^a,b^	W	M, F	palm oil	91	YA	BW, LEP, GLU, INS, TG
Hellgren L.I.^94^	SD	M	palm oil	52	YA	INS
Gregersen S.^15^ ^a,b^	W	M	coconut oil	70, 42	YA	FAT, BW
Dyrskog S.E.^14^ ^a,b^	W	M	coconut oil	70, 42	YA	BW, INS
Dong Y.M. 2010^a^ ^23^	W	M	coconut oil	21	PPP	FAT, BW, HDL, TG
Dong Y.M. 2010^b^ ^23^	W	M	soybean oil	21	PPP	FAT, BW, HDL, TG
Burckley A.J. 2005^13^	W	M	safflower oil	49	YA	BW, FAT
Chen H.^95^	SD	M	hydrogenated vegetable oil + canola oil	76	YA	FAT, BW, LEP, GLU, INS, TG
Rajia S.^96^	SD	F	hydrogenated vegetable oil + canola oil	76	YA	FAT, BW, LEP, GLU, INS, TG, SBP
Chen H.^97^	SD	M	hydrogenated vegetable oil + canola oil	83	YA	FAT, BW, GLU, INS, TG
Xue Q.^98^ ^a,b^	SD	M, F	hydrogenated vegetable oil + canola oil	21	YA	BW
Sun B.^99^a,b	SD	M, F	*na*	40	YA	FAT, BW, LEP
Gray C., Vickens M.H.^99^	SD	M	*na*	63	YA	SBP
Zaborska K.E.^101^	SD	M	*na*	*na*	YA	FAT, INS
Hou M.	SD	M	*na*	56	PPP	FAT, GLU, INS, TG

Some studies appear more than one time with different sex, age stage or timing of the intervention. Some of the studies reported more than one experiment and therefore sometimes more than one experimental group could be identified for the purposes of our analysis. The same data point was used once in the meta-analysis, even if it appeared in multiple publications. Abbreviations: M: Male, F: Female, W: Wistar, SD: Sprague Dawley, LE: Long Evans. PPP: Prepubertal/Pubertal. YA: Young Adult. A: Adult. BW: Body Weight. FAT: Body Fat. LEP: Leptin. GLU: Glucose. INS: Insulin. TRI: Triglycerides. SBP: Systolic Blood Pressure. *na: not available data*.

The characteristics of the maternal diets are shown in Supplementary Table S2. Diets differed in their macronutrient composition among the experiments. Fat content in maternal HFD ranged from 13% to 74% calories from fat, and the main fat component varied between animal-derived fats (e.g. lard) and vegetal oils. Maternal HFD usually contained more energy from fat (increase by 3 to 53% energy relative to the control group) and less carbohydrates (decrease by 16% to 52% energy relative to the control group) than control diets. Protein content was also reduced in many cases (Supplementary Fig. S2). Metabolic energy in maternal HFD ranged from 4.0 to 5.8 Kcal/g. HFD treatment was imposed on dams by feeding with well-defined commercial (35 studies), custom made (13 studies), or chow-based diets (11 studies) mostly with high fat content. Feeding was reported as *ad libitum* in 45 of 47 studies (in two studies food availability was set, refs 16 and 17) Offspring were reared on the same or similar diet to the one fed to control dams (data not shown).

### Main findings

**Figure 1:**
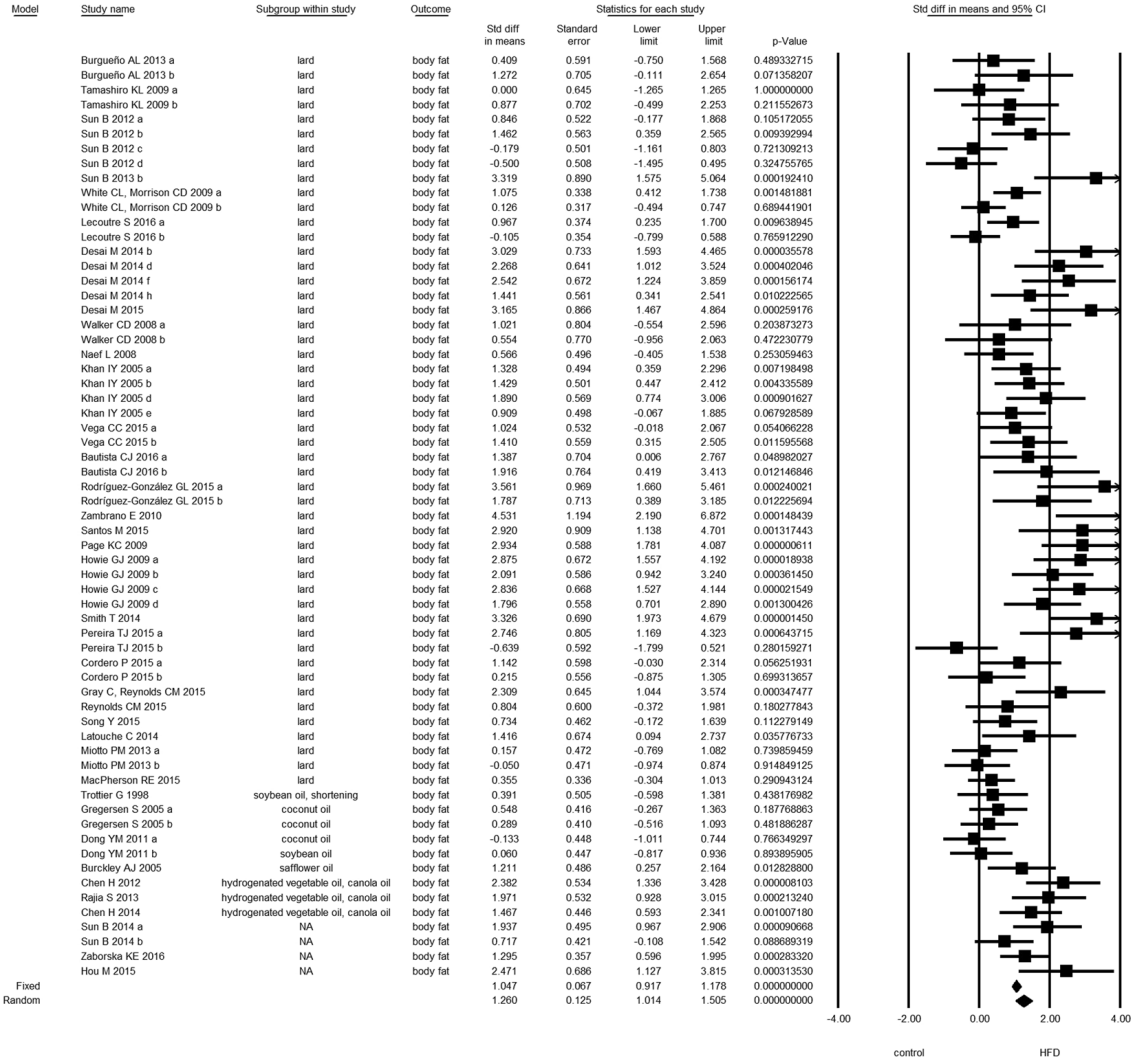
Forest Plot for Body Fat, extended dataset. Summary estimates for standardized difference in means (D, effect); the corresponding 95% CI (lower and upper) and significance (p-value) were estimated by fixed and random effects analysis. The first author of the study and the year of publication are shown. In the graph, numbers indicate D values, filled squares stand for the effect of individual studies, and filled diamonds express combined fixed and random effects. NA: not available.

**Figure 2:**
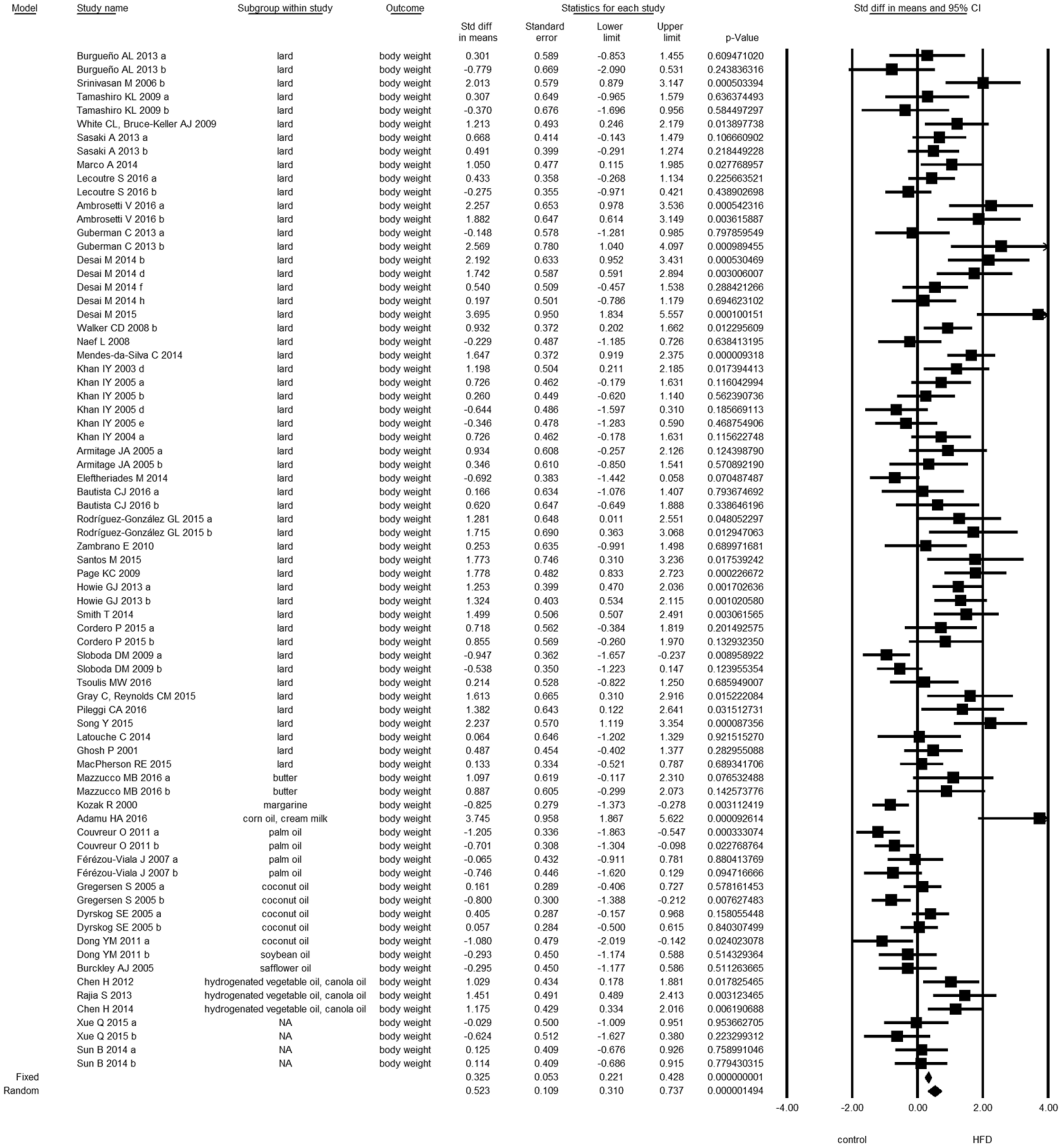
Forest Plot for Body Weight, extended dataset.

**Figure 3:**
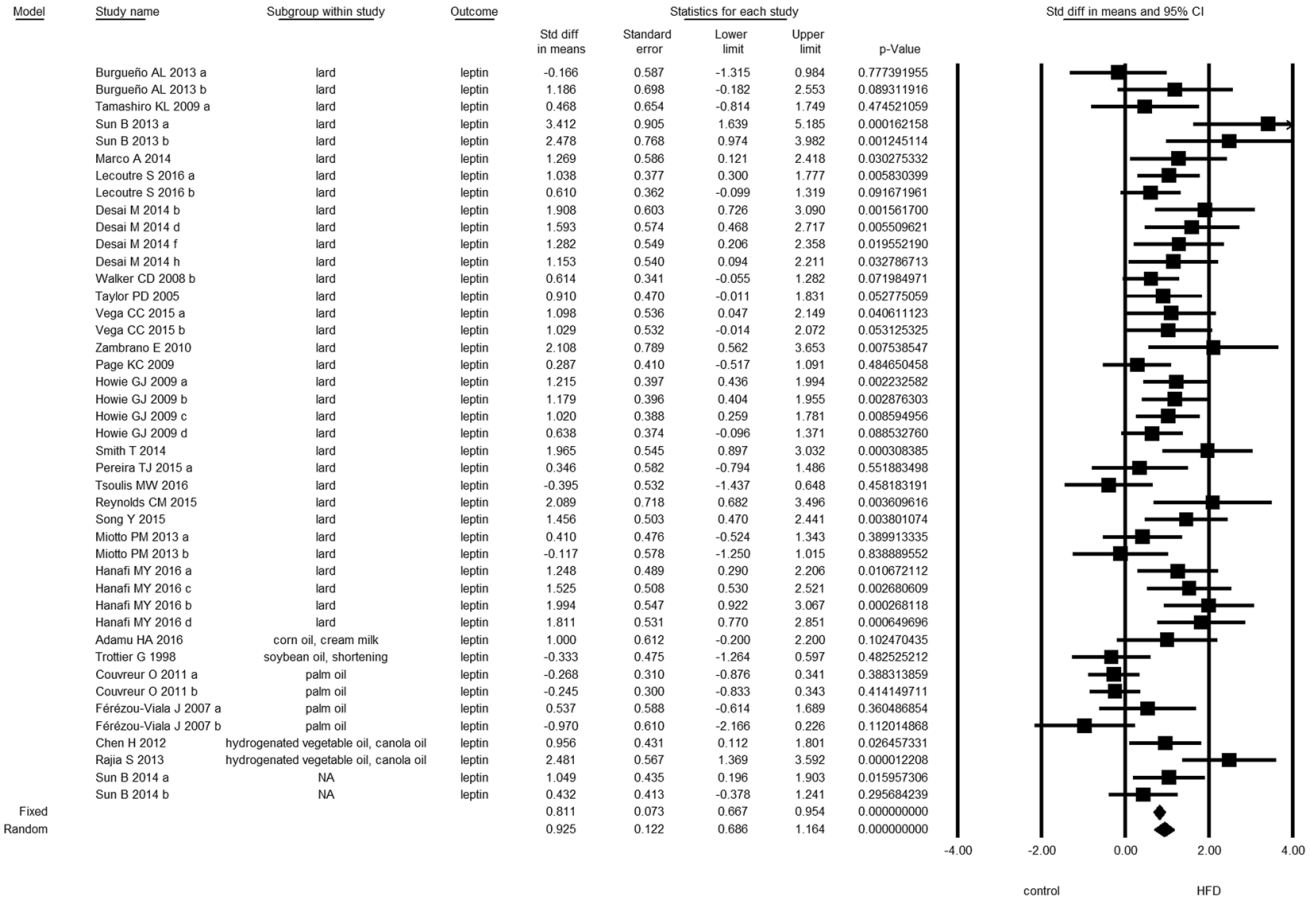
Forest Plot for Leptin, extended dataset.

**Figure 4:**
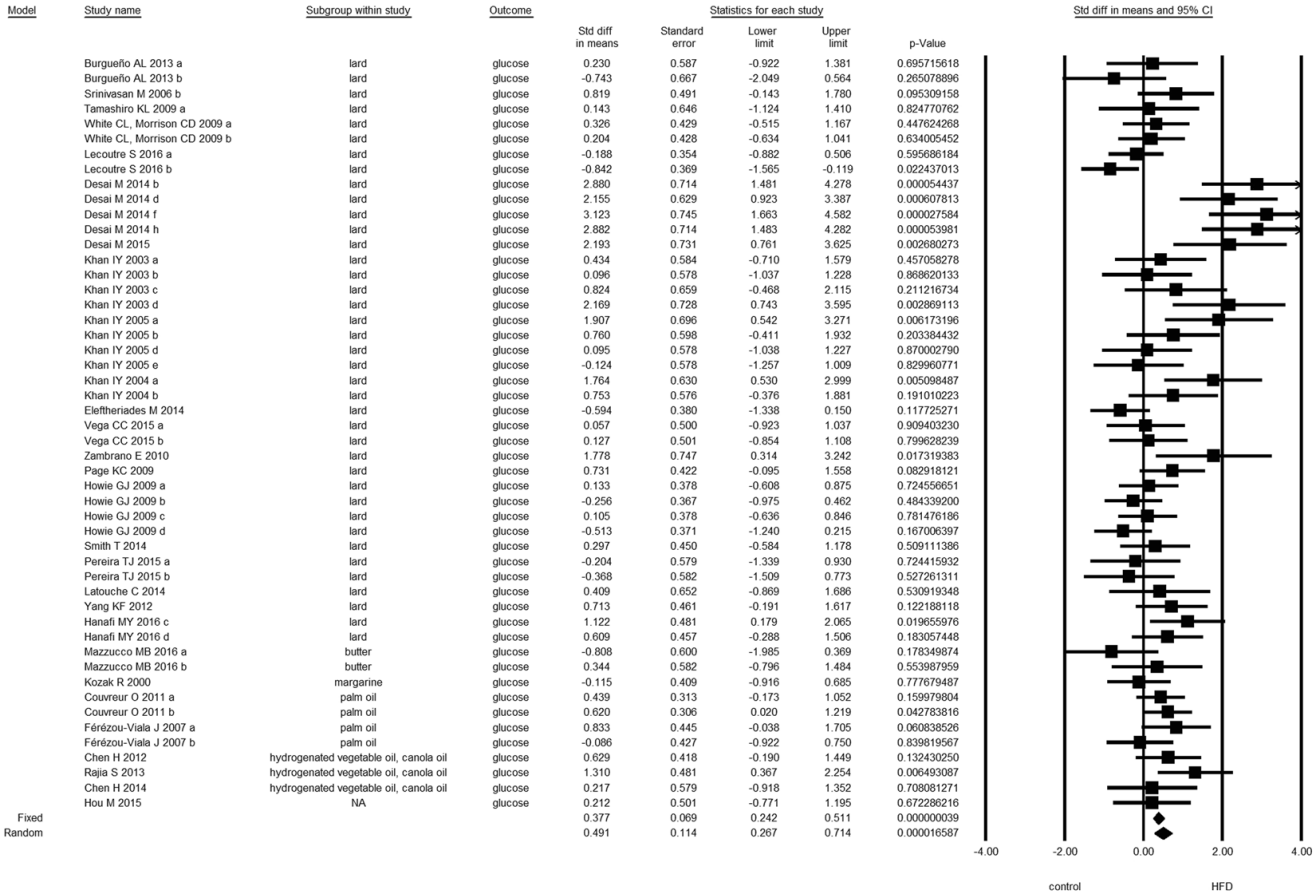
Forest Plot for Glucose, extended dataset.

**Figure 5:**
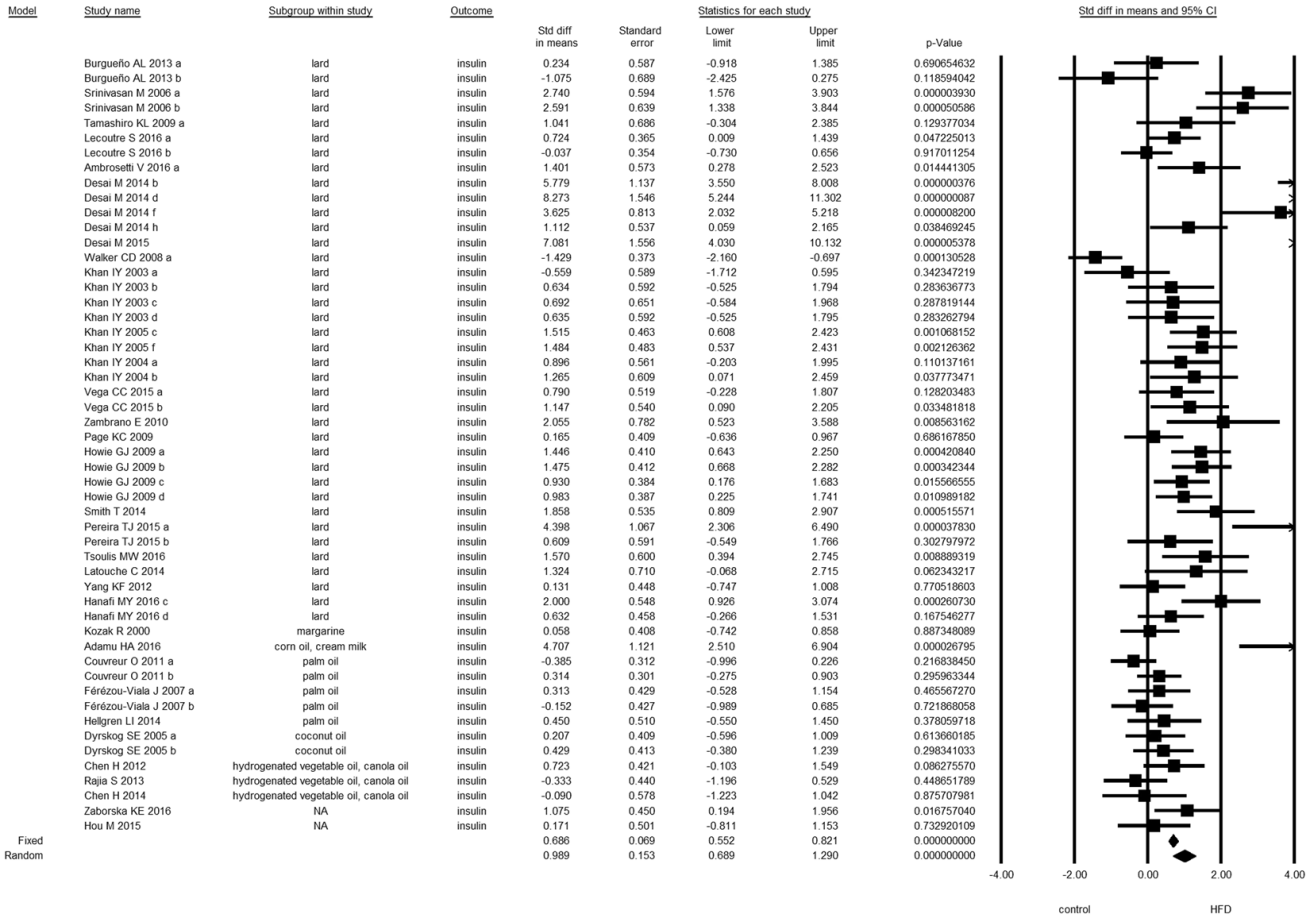
Forest Plot for Insulin, extended dataset.

**Figure 6:**
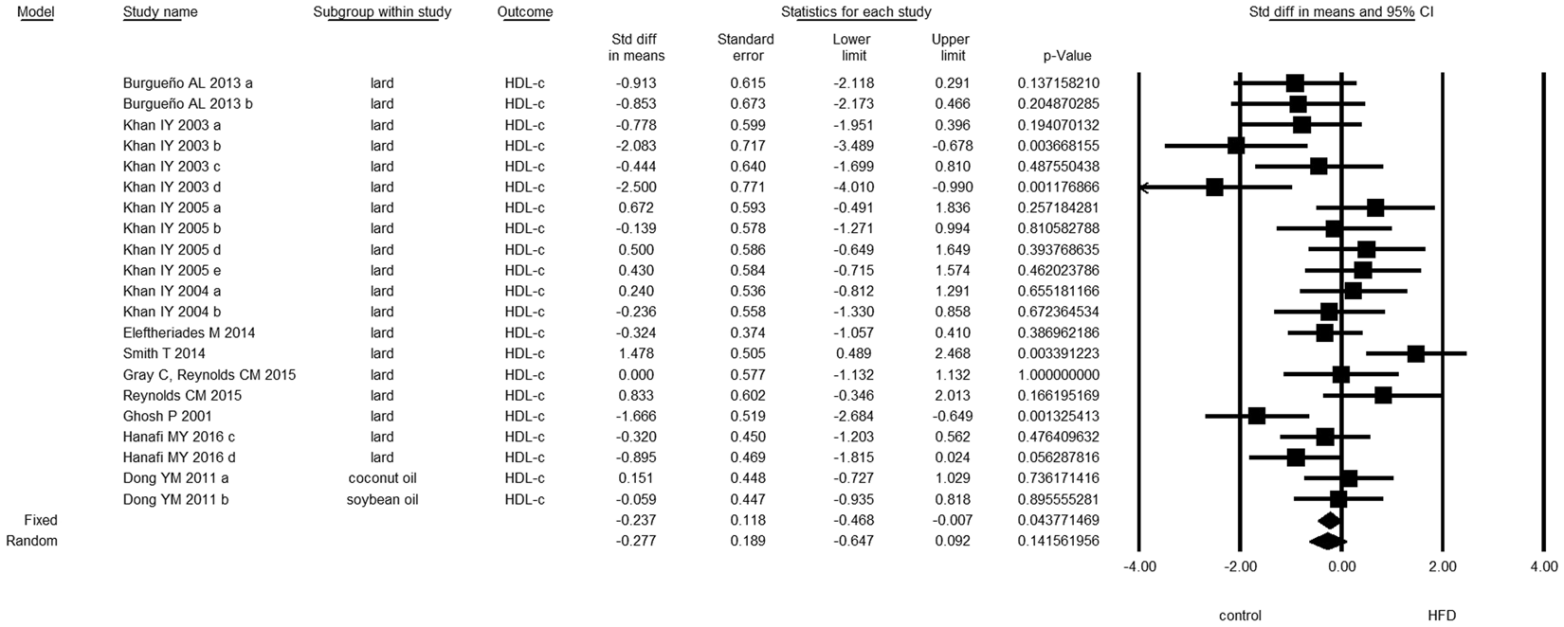
Forest Plot for HDL-c, extended dataset.

**Figure 7:**
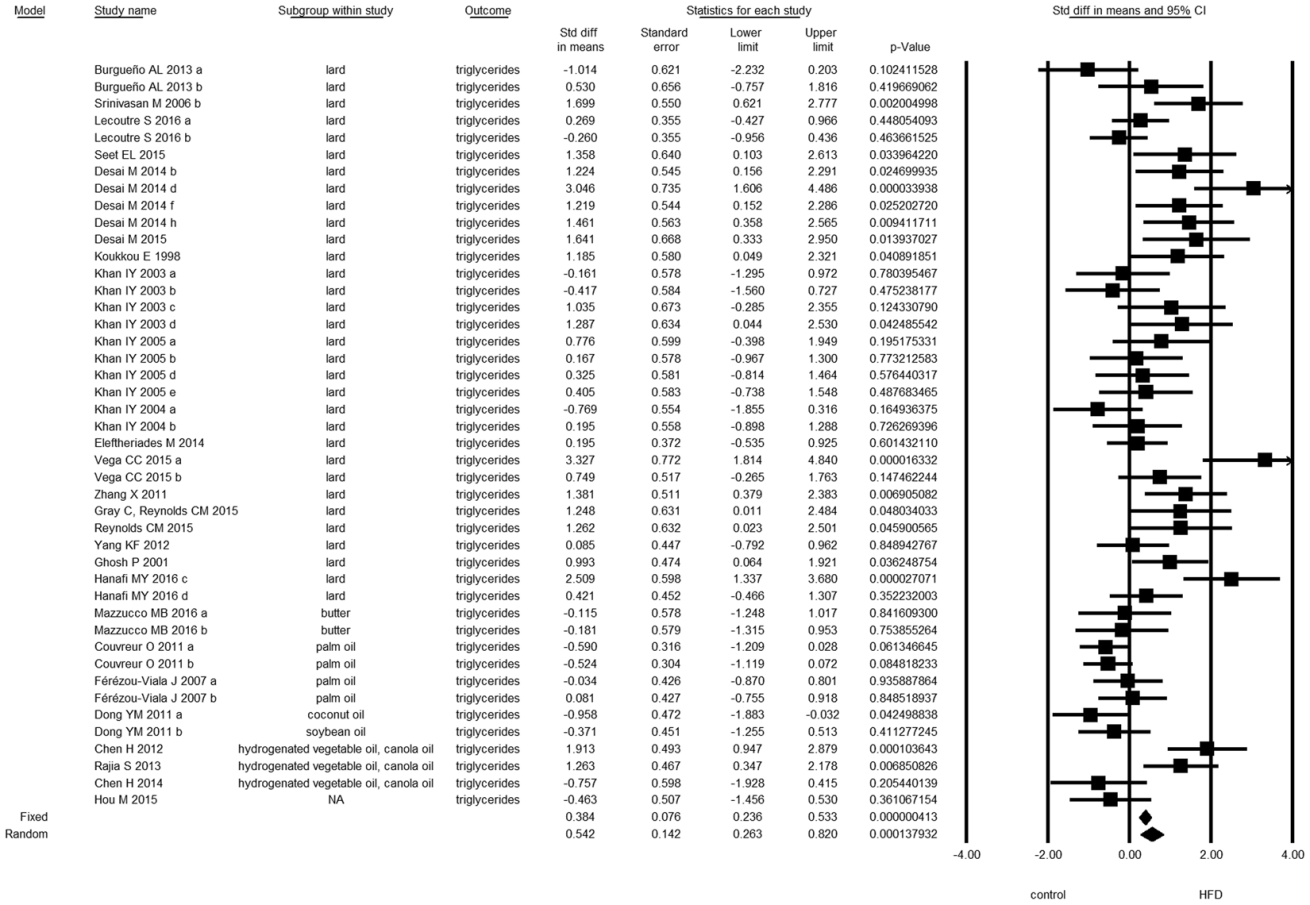
Forest Plot for Triglycerides, extended dataset.

**Figure 8:**
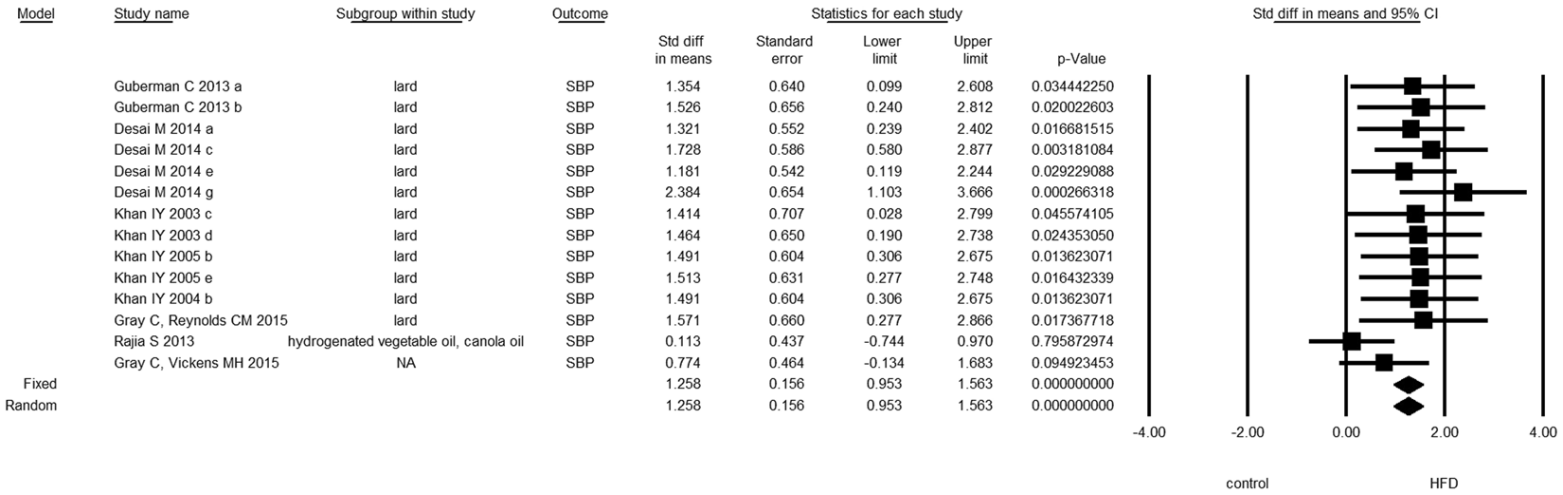
Forest Plot for Systolic Blood Pressure, extended dataset.

**Figure 9:**
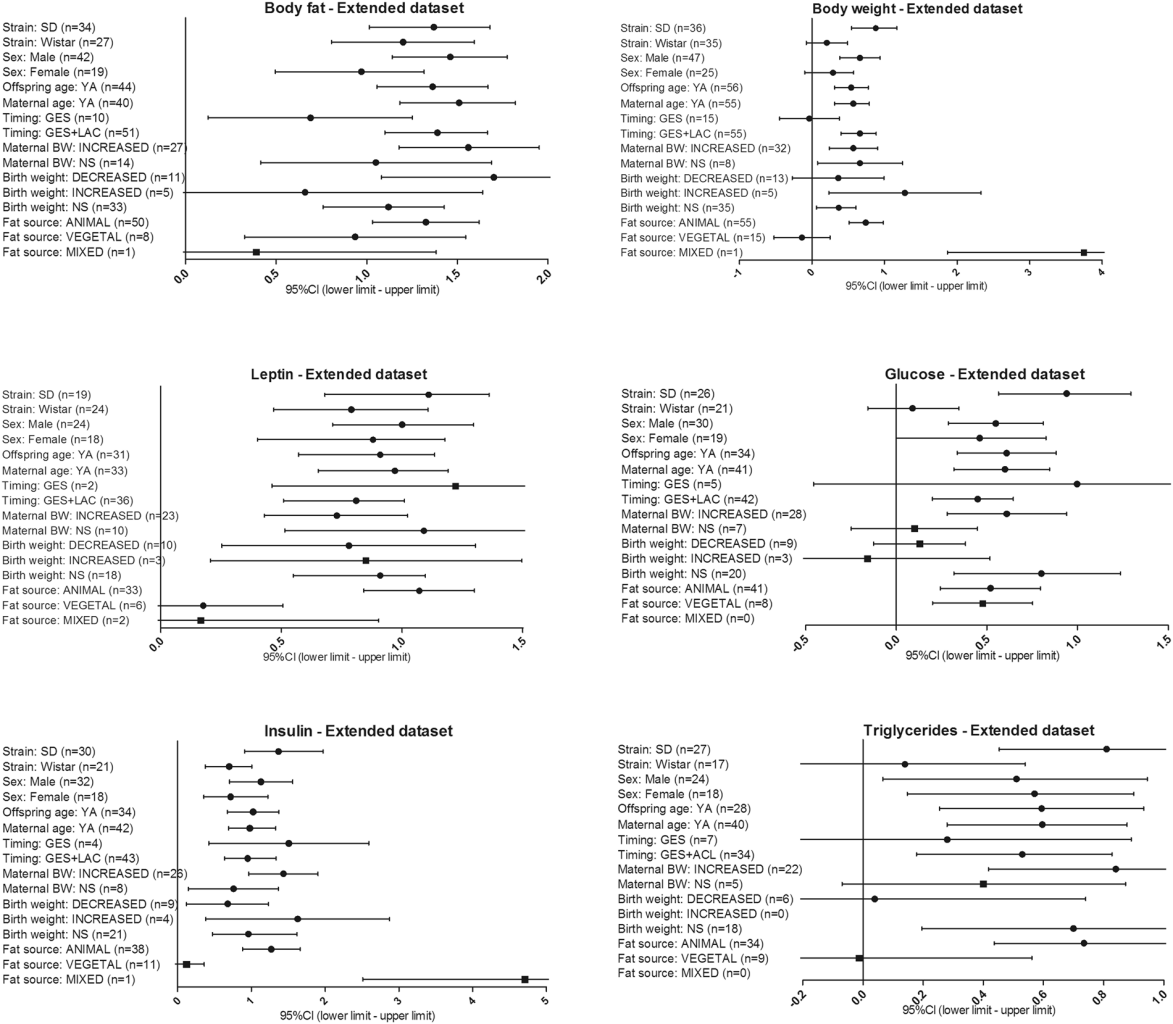
Subgroup analyses for extended dataset. Horizontal lines represent the 95% CIs for the data. The summarized effects (D) are considered statistically significant when their 95% CIs do not cross zero. We used a random-effect model (filled circles) whether heterogeneity was observed, while the fixed-effect model was applied in the absence of heterogeneity (filled squares). Included moderators for the extended data set are: strain (Sprague Dawley, Wistar), sex (male, female), offspring age at testing (young adult), maternal age (young adult), intervention timing [perinatal (gestation and lactation) vs. restricted to gestation period], maternal body weight (increased, not increased), birthweight (decreased, increased, not different), and main fat source (animal, vegetal, mixed; extended dataset). Subgroup analyses for HDL-c extended dataset is available in Supplementary Fig. S4. Subgroup analysis of subsets where heterogeneity was not significant was not performed (SBP subset). Abbreviations: n: number of data points, SD: Sprague Dawley, YA: Young Adult, GES: gestation period only, GES + LAC: gestation and suckling periods.

Overall, we found evidence for the effects of maternal HFD consumption around gestation time on the investigated offspring outcomes with just one exception, HDL-c (Figs 1–8, Table 2). The pooled estimates for the effects of maternal HFD consumption on outcomes are summarized in Table 2. Changes in body fat, body weight, leptin, glucose, insulin, triglycerides and SBP in offspring of dams exposed to HFD were significantly different from those in offspring of control-fed dams. Heterogeneity was not detected in SBP subset (I-squared = 0, Q statistic p-value = 0.5). Nonetheless, moderate to high levels of heterogeneity were found in the other seven data subsets (I2 between 59.8 and 78.5%, Table 2); therefore, we performed a random-effect analysis in the extended dataset, except for SBP. When separate analyzes were ran for studies using experimental diets based on animal fat, heterogeneity was found in every subgroup, except for the SBP subset as expected (extended dataset, Fig. 9).

**Table 2:** Data analysis summary.

Outcome	Dataset	N	Effect size		One study removed	Heterogeneity	
			Std diff in means ± Std error	p-value	p-value	I-squared	Q statistic p-value
Body weight	extended	75	0.52 ± 0.11	1.10-6	1.10-6	75.1	<0.001
	main	49	0.81 ± 0.13	<1.10-8	<1.10-8	67.1	<0.001
Body fat	extended	63	1.26 ± 0.13	<1.10-8	<1.10-8	69.9	<0.001
	main	47	1.38 ± 0.16	<1.10-8	<1.10-8	70.8	<0.001
Leptin	extended	43	0.93 ± 0.12	<1.10-8	<1.10-8	61.8	<0.001
	main	26	1.02 ± 0.10*	<1.10-8	<1.10-8	32.6	0.06
Glucose	extended	50	0.49 ± 0.11	2.10-5	2.10-5	61.6	<0.001
	main	33	0.64 ± 0.16	9.10-5	9.10-5	68.8	<0.001
Insulin	extended	52	0.99 ± 0.15	<1.10-8	<1.10-8	78.5	<0.001
	main	33	1.37 ± 0.22	<1.10-8	<1.10-8	81.0	< 0.001
HDL-c	extended	21	−0.28 ± 0.19	0.1	0.1	59.8	<0.001
	main	14	−0.21 ± 0.29	0.5	0.5	70.6	<0.001
Triglycerides	extended	44	0.54 ± 0.14	0.0001	0.0001	70.1	<0.001
	main	26	0.83 ± 0.17	1.10-6	1.10-6	60.0	<0.001
SBP	extended	14	1.26 ± 0.16*	<1.10-8	0.0003	0.0	0.5
	main	12	1.52 ± 0.18*	<1.10-8	<1.10-8	0.0	1.0

For each outcome, effect size stands for Cohen’s standardized difference in means (D), which was the difference of means between groups (experimental vs. control) divided by the common within-group SD. We used a random-effect model if heterogeneity was observed, while the fixed-effect model (*) was applied in the absence of heterogeneity. We performed sensitivity analyses by omitting one study at a time and calculating the pooled effect size for the remainder of the studies. Heterogeneity was evaluated with the Q statistic and I-squared statistic.

The main data set was processed and meta-analyzed in the same manner as the extended data. We extracted 240 effect sizes from 44 studies, and the analysis for the main data subsets revealed the same pattern of effects as the meta-analysis of the extended dataset (Table 2, Supplementary Fig. S3). Namely, we confirmed the effect of the maternal nutritional manipulation on the seven outcomes and the effect on the changes in HDL-c levels remained not significant. Heterogeneity was found with respect to all outcomes except for leptin (I-squared = 32.6, Q statistic p-value = 0.06) and SBP (I-squared = 0, Q statistic p-value = 1, Table 2) in main dataset. To evaluate the robustness of our results against influential studies, a leaving-one-out sensitivity analysis was performed. All sensitivity analyses confirmed the stability of our analysis as no influential individual study could be identified (data not shown, p-values available on Table 2).

Subgroup analysis for subsets that have proven to have heterogeneity is shown in Fig. 9 (summarized effects, D for the extended dataset), Supplementary Fig. S4 (HDL-c extended dataset) and in Supplementary Fig. S5 (main dataset). We decided not to focus on results from subgroups consisting only of few data points. Heterogeneity, where it could be tested, could not be eliminated except for some exceptions in main dataset (Supplementary Fig. S5). Exceptions are: 1) when limiting the analysis to Wistar rats, heterogeneity was solved for glucose and insulin subsets; and 2) when repeating the analysis on chow-based diets only, heterogeneity was solved for four outcomes: body fat, glucose, insulin and triglycerides. Restricting the analysis to males or females, Sprague Dawley strain, young adult offspring, young adult dams, perinatal intervention, or commercial lard-based experimental diet, did not eliminate the heterogeneity.

A diet enriched with animal fat mainly consists of non-essential fatty acids (saturated and polyunsaturated ω-9) as opposed to that of vegetable origin that primarily contains essential fatty acids (polyunsaturated ω-6 and, to a lesser extent, ω-3). The results of the subgroup analysis quantifying differences between different main fat sources are included in Fig. 9. Experimental offspring is not likely to have the full plethora of deleterious effects when their mothers were fed vegetable fats, given that the effect of maternal HFD on offspring remained only on two outcomes: body fat and glucose levels. However it should be bear in mind that, because the number of studies was too small to create several groups, we have grouped together diets with different fatty acid composition under the denomination of vegetable fat- rich diets. The evidence suggests that the consumption of polyunsaturated fatty acids (PUFAs), in particular long-chain PUFAs, during crucial periods of fetal development plays a beneficial physiologic and metabolic role in the health of offspring^18^. However, only few studies have been undertaken to directly compare the effects of the differences in the type of fat in the maternal diet; thus, it is yet not possible to determine whether the maternal intake of a specific fatty acid type during pregnancy and/or lactation correlates with the development of a particular phenotype of the offspring. The metabolic consequences of the maternal consumption of different types of fatty acids have been reviewed elsewhere^18^.

We observed that the effect of maternal lard consumption on the outcomes was independent of the type of diet (Supplementary Fig. S5), except for triglycerides. We found that when experimental dams were given a chow-based HFD, their offspring had no hypertriglyceridemia; however it should be noted that these results derive from three studies of the same group of authors^19–21^.

Subgroup analysis did not detect any substantial effect on levels of HDL-c in any subgroup.

We used meta-regression models to uncover the potential influence of differences in experimental protocols, such as: 1) offspring age at measurement, 2) maternal age, 3) duration of maternal dietary manipulation, 4) litter size, 5) increase in fat content in experimental diet with respect to control diet, and 6) protein-to-non protein ratio in experimental diet. Statistical summaries of results from meta-regression are shown in Supplementary Fig. S6. Visual inspection of the effect plots suggested that some of the results obtained could be driven by single data points, so we decided not to focus on those results.

Offspring’s age is a biological variable likely to contribute to variability in results. The offspring’s age at the time of outcome measurement was 151 ± 89 (mean ± SD) days, indicating that most of the measurements were taken on young adult individuals. Restriction of the analysis only to the young adult subgroup (PND63 to PND209) confirmed results but did not eliminate heterogeneity (Fig. 9 and Supplementary Fig. S5). Meta-regression analysis was ran to estimate if variation within this group depends on age (Supplementary Fig. S6), and according to our analyses age of offspring at testing had detectable influence on four outcomes: body fat (slope ± SE = 0.01 ± 0.002 and 0.02 ± 0.003 for extended and main datasets respectively), leptin (slope ± SE = 0.007 ± 0.0003, main dataset), insulin (slope ± SE = 0.006 ± 0.0002, extended dataset) and SBP (slope ± SE = 0.01 ± 0.006, extended dataset).

Young female dams were used in included studies, with maternal age ranging from 42 to 154 days at mating or conception, except for two studies designed with middle aged dams (32 weeks of age). When we repeated our statistical analyses on the young adult subset, heterogeneity was not solved (Fig. 9 and Supplementary Fig. S5). As shown in Supplementary Fig. S6, maternal age had detectable influence on blood glucose (main and extended dataset), blood insulin (main dataset) and triglycerides levels (main and extended dataset). Effect sizes were likely to be bigger when dams were younger. This finding is partly in disagreement with our hypotheses that older mothers would produce offspring that are more susceptible to MetS in a HFD environment.

Limiting the analysis only to studies where intervention was done during the perinatal period, by means of excluding studies where manipulation was done exclusively during gestation, heterogeneity was still evident. Through regression we observed that within this group of perinatal intervention, the starting point of manipulation appeared to significantly affect offspring insulin (slope ± SE = 0.004 ± 0.002 and 0.004 ± 0.002 for extended and main datasets respectively) and glucose levels (slope ± SE = −0.005 ± 0.002 and −0.006 ± 0.002 for extended and main datasets respectively).

Other differences in the experimental protocols such as litter size and fat content in experimental diet had poor detectable influence on the studied outcomes. Litter size threshold is usually set to prevent the possibility of under- or overnutrition during suckling. For 52 cohorts, upon birth litter sizes were adjusted to the same number of pups per dam, usually 8 pups/dam (ranging from 5 to 11). In the remaining 16 cases information on standardization was not available. Litter size should be critical in determining how and to what extent the metabolism is affected, however we did not find a statistically significant litter size effect, with few exceptions. It is presumed that individuals in small litters have greater access to milk during the suckling period; however, in narrow ranges of litter sizes this effect would be negligible. By decreasing the number of pups in the litter, increases the effect of maternal HFD only on leptin, insulin and triglycerides (slope ± SE = −0.3 ± 0.07, −0.2 ± 0.06 and −0.1 ± 0.05 in extended dataset respectively, Supplementary Fig. S6).

When analyzing the extended dataset we found that fat content affects two outcomes contrary as expected: body weight (slope ± SE = −0.02 ± 0.006) and insulin (slope ± SE = −0.02 ± 0.006). As discussed below, it could be associated with a lower protein content of HFD and then related to a decrease in the lean body mass; however these findings were not observed when repeating the analysis only on studies of dams fed animal fat (data not shown) or in main dataset. This could also indicate that the effects of vegetable oil rich- diets confound results. Main dataset is a refined subset that includes studies based on lard-based diets of a relatively narrow fat content (40–60 kcal%). Given these conditions, only glucose concentration increased depending on experimental fat content (slope ± SE = 0.03 ± 0.01, main dataset). Besides, the severity of the protein dietary manipulation has also shown to influence results. The effect of prenatal HFD on body weight, leptin, insulin and triglycerides depended on the proportional protein content in experimental diet (slope ± SE = 3.7 ± 0.7, 5.6 ± 1.1, 6.0 ± 1.2 and 6.1 ± 1.2 for body mass, leptin, insulin and triglycerides in extended dataset). When analysis was however repeated on main dataset, within studies where experimental diet had no extremely decreased protein content, variation was observed only on insulin (slope ± SE = 3.7 ± 1.8).

Finally, as expected, we did not find any statistically significant overall effects of moderators on SBP besides the aforementioned effect of offspring age.

### Methodological quality and publication bias

A summary of the methodological assessment for each included study is shown in Supplementary Table S3. The methodological quality scores ranged from 1 to 5, with 72% of studies scoring 4 or 5 points. In general, study design and reported statistics raised no concerns about good scientific practice. The median impact factor for all the included studies was 2.77 (J Neurochem, IF2014: 0.09 – Diabetes, IF2009: 8.35). We used funnel plot asymmetry to detect any publication bias in the meta-analysis, and Egger’s regression test to measure funnel plot asymmetry (Supplementary Fig. S7). In general, visual inspection of funnel plots indicated little to moderate asymmetry, with an intercept of Egger’s regression significantly different from zero. This finding suggests that publication bias cannot be completely excluded as a confounder of our meta-analysis. It remains possible that small studies yielding inconclusive results have not been published. No evidence of publication bias was found in two data subsets: HDL-c subset in extended dataset and SBP subset in main dataset.

## Discussion

We conducted a meta-analysis of the effect of maternal HFD on offspring’s phenotypic characteristics of the MetS. Results indicate that a maternal HFD around gestation appears to have a detrimental effect on the studied outcomes, relative to the control group. Maternal HFD resulted in increased body fat, body weight, leptin, glucose, insulin, triglycerides levels and SBP in young adult offspring.

We found no general effect of maternal HFD on HDL-c. The simplest explanation of the limited evidence for changes in HDL-c in the offspring of nutritionally challenged mothers is that such an effect is too small to be statistically detectable with the current sample (21 and 14 data points in extended and main datasets, respectively, although with a acceptable sample size of 316 animals). Few authors have evaluated HDL-c concentrations. Of course it could also happen that early-life programming of offspring HDL-c levels via maternal nutrition does not occur in rats. A third possible explanation is that offspring may not show some of the effects of maternal nutritional programming at some point after offspring had access to a standard diet. Optimal nutritional conditions could potentially reverse the effects of the maternal HFD^22^. However this hypothesis remains to be explored because in our meta-analysis no effect on HDL-c was detected in the prepubertal/pubertal group (2 datasets, data not shown)^23^. Finally, dyslipidemia is central to the diagnosis of the MetS, however it should be mentioned that the rat is not ideal as a model of human dyslipidemia because of the different lipid metabolism, and in general the rat is resistant to the development of atherosclerosis^24^.

We observed significant medium to high heterogeneity across all of our data, except in SBP data subset in both extended and main datasets, and in leptin subset in main dataset (Table 1). Heterogeneity suggests that only under some specific conditions, maternal nutrition may negatively influence offspring body fat, body weight, glucose, insulin and triglycerides levels. Anyway, a random effects-analysis was performed, in which heterogeneity is no longer an issue. Heterogeneity can be partly attributable to some of the moderators included in our study. To further explore heterogeneity that may be associated with differences in strain, offspring traits (sex and age) and experimental design (i.e. lard-based diet type and timing of manipulation), we performed sub-group analysis (Fig. 9 and Supplementary Fig. S5). None of the categorical variables clearly explained the observed variation among studies, except for rat strain and type of lard-based diet in specific subsets. Due to the unbalanced nature of sample sizes in our data subsets, we could not perform all of the planned subgroup analysis.

Overall, according to subgroup analyses, the differences in the experimental protocols used in the included studies had some detectable influence on body mass, plasma glucose and triglycerides levels. First, we observed strain -specific effects on these data subsets (Fig. 9 and Supplementary Fig. S5). Maternal HFD appeared not to affect body weight (extended dataset), plasma glucose (both datasets) or triglycerides levels (both datasets) in Wistar offspring. Thus, strain can influence the conclusions of different studies. Otherwise, it is still possible that effects were too small to be reliably detected.

On the other hand, the existence of sex specific differences in animal models of developmental programming is well described in currently available literature. The molecular and phenotypic outcomes of adverse *in utero* conditions are often more prominent in male than female offspring, although there is short regard given to the basis for this observation in most studies^25^. We have previously hypothesized that the metabolic programming effect of maternal HFD is sex specific^26^. Disparities in the sex specific genome and epigenome, the influence of sex hormones, and differences in placental function are important factors in this regard^27, 28^. Recently, some studies have reported a gender-specific regulation of the expression of genes involved in varying metabolic pathways in response to a HFD or a cafeteria diet^29–31^. A proteomic study have identified numerous proteins showing sex dimorphism in skeletal muscle in response to HFD feeding^32^. The lower tendency to undergo MetS in response to HFD in female rats may be related to lower reliance on lipid as an energy fuel, lower lipogenesis, as well as increased mitochondrial oxidative capacity^33^.

Differences between the sexes appear both morphologically and in the transcriptome at a very early time in mammalian development. Apart from innate differences between the sexes, male and female fetuses may adapt differently to early-life nutritional conditions. Several studies reported sex specific differences in the placenta during fetal life. Mao J. *et al*. have examined the impact of diet (very-high-fat, low-fat and chow diets) and fetal sex on placental gene expression in mice and interestingly found that each diet provides a distinctive signature of sexually dimorphic genes^34^. Maternal diet might also influence imprinted gene expression and epigenetic DNA methylation in male and female foetuses. The placentae of foetuses from mothers fed a HFD during pregnancy displays changes in both the expression of selected imprinted genes from different clusters, and in DNA methylation, with these changes differing between sexes^35^.

We found a sex-specific effect of the maternal nourishment on body weight- extended data subset (Fig. 9) and on glucose- main data subset (Supplementary Fig. S5) when data from male and female offspring were analyzed separately. We additionally ran a meta-regression with gender as predictor variable to estimate the impact of sex on effect size. Given that values of 1 for males and 2 for females were arbitrarily assigned, a negative slope would indicate a larger effect size in males. Specifically, we observed negative slopes in 3 outcomes: body fat, body weight and HDL-c (Supplementary Fig. S6), and no variation for sex in the other data subsets. A comparative microarray analysis in soleus muscle between male and female rats revealed 35 differentially expressed transcripts in response to HFD^31^. It has been suggested that lower weight gain in HFD female rats is, at least in part, associated with lower expression of genes involved in glycolysis and higher expression of genes involved in fatty acid oxidation^31^.

In terms of sexual dimorphism in animal models of developmental programming, phenotypic differences exist not only at the outcome level (eg. the difference reported here in body weight), but also at the molecular level. It is possible that even the same phenotype may be a consequence of different molecular mechanisms in males and females.

Timing of the maternal nutritional insult is another important factor to take into consideration. Subgroup analyses revealed that the effect of maternal HFD was unequivocally significant with a perinatal intervention including suckling period. But alternatively, in studies where maternal HFD was applied only during gestation, maternal effects were not significant on body weight (extended and main dataset), plasma glucose (extended and main dataset) and triglycerides levels (main dataset). This may be either because the effects were too small to be detected, and/or because we have insufficient data to detect the impact. This may also be because some outcomes are indeed affected by the duration of the manipulation. It is important to emphasize that this finding is in accordance with a previous meta-analysis where exposure to a maternal obesogenic diet that extended into the suckling period was more influential for programming of the offspring’s adult body weight than the exposure during gestation only^36^. Evidence in rats from cross-fostering studies in models of fat feeding and other models of postnatal overfeeding clearly shows that the suckling period is critical for developmental programming^7, 9^.

The nutritional composition of the breast milk is likely to be affected by the pre-existing maternal obesity, the diet during pregnancy and the diet during lactation. It seems clear that alterations in milk composition, pup ingestive behavior and maternal care during lactation may contribute to the long-term metabolic changes induced by the maternal HDF, however this parameters are not frequently addressed. HFD fed to rats during pregnancy and lactation increases milk lipid concentration^37–40^. More specifically, HFD changes the fatty acid composition by increasing the long-chain fatty acid content at the expense of medium-chain fatty acids^41^. Furthermore, feeding a cafeteria diet only during the suckling period also increases the fat, the energy and the long-chain fatty acid content in the milk of obese rats^42^.

As mentioned in results section, the effect of prenatal HFD depended also on the time at which dam diet manipulation had started in mothers who received HFD during gestation and lactation. Owing to differences in the duration of the high-fat dietary regimen, the maternal intrauterine environment may be differently affected, and consequently the severity of the metabolic abnormalities in the progeny would be different. Longer durations of maternal HFD contributed to the higher insulinemia, but to lower glycemia, in the experimental offspring relative to the control offspring. The direction of the effect on blood glucose was in opposition to that predicted. We predicted that offspring of mothers experiencing longer exposure to HFD should be “programmed” to have an overall worse phenotype. Long-term HFD regimen better represents the present dietary habits of humans in Western societies^43^. However there is little research on this specific issue. Howie GJ and colleagues^44^ have shown that lifetime consumption of a HFD appears to have similar influences on offspring phenotype compared to HFD consumption restricted to pregnancy and lactation alone^44^. Anyway, the effect of diet duration on glycemic control deserves further study.

The influence of protein content was also analyzed by meta-regression. As discussed previously, the fat component usually replaces carbohydrate and/or protein in experimental diets leading to an unbalanced diet composition with respect to macronutrients, especially protein (Supplementary Fig. S2), which might confound the fat effect. Indeed, the protein-to-non protein ratio is significantly decreased in custom-made and chow-based diets with respect to commercial diets (ANOVA p < 1.10-5, data not shown) showing a that those diets have greater nutritional imbalance. Lagisz M. *et al*.^36^ in their previous meta-analysis found that decreased offspring body weight was more likely when maternal obesogenic diet contained low ratios of protein^38^. We found similar results. Indeed, increased effect sizes with increasing levels of protein-to-non protein ratios are also seen for leptin, insulin and triglycerides levels.

Finally, in our study we have also focused on other two moderator variables considered to be of biological significance: maternal weight and birthweight. An interesting fact to consider is that none of these two factors were dependent of energy from fat and protein-to-non protein ratio in experimental diet (ANOVA, data not shown). It has been previously described in a narrative review of data from eleven studies that poor glycemic control in male offspring exposed to perinatal HFD appears to be independent of maternal obesity and birthweight^12^. For the purpose of this meta-analysis we collected data on whether body weight of experimental dams was reported as significantly increased or not in comparison to control dams during gestation. This data represents in some studies not only the gestational weight gain but also pre-conceptional weight gain. Interestingly, the effects of maternal HFD on glycemia and triglyceridemia appear to be dependent on maternal obesity (Fig. 9 and Supplementary Fig. S5). Then, in mothers who did not present high body weight, no effects on offspring glucose and triglycerides were observed. This could also be attributed to the minority of our data coming from studies where dams had normal weight: augmented body weight was presented in 68% dams. Increases in body size, body fat, leptin, insulin and SBP levels occurred in offspring of dams with elevated body weight but also in dams with normal weight. As both obesity and maternal weight gain are commonly induced by feeding dams an obesogenic diet during gestation, it is hard to isolate the effects of maternal gestational weight gain, maternal obesity and maternal diet per se. Therefore, any observable outcomes in offspring may be a result of the diet, maternal obesity, or an interaction between the two. Furthermore, a deeper understanding behind the causal factors associated with maternal obesity, such as hyperglycemia, hyperinsulinemia, and hyperleptinemia, would be of valuable help^10^.

Epidemiological studies and animal models have linked birthweight to risk of adult obesity and MetS, including insulin resistance. Deviations from “optimal” *in utero* growth, be it from limited or excess nutrition, increase the relative risk of MetS in adulthood^6^. The association between birthweight and obesity was reviewed elsewhere^6^. Maternal HFD has been reported to have variable effects on birthweight. In our meta-analysis, leptinemia and insulinemia were increased regardless of birthweight (Fig. 9 and Supplementary Fig. S5). When analyzing the extended dataset we identified three outcomes not affected by the maternal diet in low birthweight experimental offspring: body weight, blood glucose and plasma triglycerides (Fig. 9); although this lack of effect was observed only on glucose levels in main dataset (Supplementary Fig. S5). Interestingly, the effects on body fat and glycemia were not observed in the subgroup of large babies; and we have no concluding data on the effect of maternal HFD on triglycerides and HDL-c when birthweight is increased. Again, conclusions should be taken with caution because these results may be subjected to effects too small to be reliably detected, or to insufficient data to uncover an effect. The number of effect sizes in extended dataset range from 5 to 13 in low-birthweight subgroup and from 0 to 5 in high-birthweight subgroup. These data also show that in general there are no alterations of the birthweight in this model, which it is in accordance to a previous review on maternal HFD^12^. This fact establishes further evidence of *in utero* programming since adult offspring still consistently exhibit metabolic abnormalities.

Through conducting this review, results enable us to provide preliminary recommendations for future research in the field of developmental programming of the MetS. Firstly, in order to obtain optimal and reproducible benefits, animal fat is more effective than vegetable oils. More specifically, lard based diets (rich in saturated fatty acids) with fat energy content between 40 and 60% are recommended. Within this range, only plasma glucose concentrations appeared to be sensitive to the level of fat content in maternal diet. Secondly, optimizing macronutrient balance in the maternal diet is very important. Differences across studies in protein amounts available to the dams could potentially explain some of the contradictory experimental results^38^. As long as the protein difference between control and experimental diets remains below 10%, there would not be much variation in the phenotype, with one exception: lower protein content is associated with lower insulin concentration in offspring. Third, lactation is a critical period for programming offspring metabolism later in life. Lagisz M and colleges^36^ have previously provided review-generated evidence of the importance of the timing of diet manipulation^38^. Antenatal intervention alone can contribute to contradictory results. On the other hand, the beginning of the intervention would not be an important variable except for two outcomes, insulinemia and glycemia. Fourth, maternal obesity might be a key factor determining the extent of maternal effects on offspring phenotypes. Finally, we suggest studying the response of both sexes to maternal dietary interventions, and where possible, effects should be investigated in a sex-specific manner. We strongly discourage reporting outcomes of mixed-sex groups.

Some aspects of our meta-analysis deserve discussion. Despite extensive searches, some of our findings are based on a limited number of rats and strains of rats and, thus, our conclusions are not necessarily transferable to other laboratory rodents or mammals.

We found evidence for publication bias in our data sets that warrants further investigation of the factors influencing offspring phenotypes in later life. However, given the levels of quantified heterogeneity within our data sets and the described methodological differences in experimental designs, we believe funnel plot asymmetry may be ascribed to between-study variation, but we cannot disregard publication bias favoring significant results. It is strongly recommended the publication of good quality papers even with negative results.

Maternal HFD studies seem even more likely to be confounded by the details of experimental set-ups. Experimental designs varied widely among the studies included in our dataset. Moreover, in the present meta-analysis we did not take into account the physical form of the diet (powder, pellet, liquid), nor the methods of determination of the outcomes. Varied assessment tools were implemented in the determination of body fat and blood pressure. For example, adiposity was assessed by markedly different methods such as calorimetry, bioimpedance, nuclear magnetic resonance, and dual x-ray absorptiometry (8/37 studies), or alternatively was estimated as fat pad weight (24/37 studies). We do not expect publication bias to exist strictly in these data sets because most of the included articles were not originally designed to investigate the effect of maternal HFD on the phenotypes that are here studied as primary outcome. Researchers usually are specially concerned with testing of hypotheses rather than with rigorous animal model generation. In line with this, the quality of the included studies was scored as acceptable in 49 of 68 cases, with 63.0% of the included studies reporting randomization of the animals (Supplementary Table S3). Many papers on animal experiments are incomplete in reporting the necessary details^45, 46^. The quality of animal experimental work could be improved by standardized animal models, and therefore the reliability of its findings. Standardization in future studies may provide a good platform with which to evaluate the effects of maternal HFD and will help to reduce potentially confounding effects for among-study comparisons. Furthermore, today the 3Rs (Replacement, Reduction and Refinement) are increasingly seen as a framework for conducting high quality science. Improved experimental design would minimize the number of animals used per experiment or study.

In summary, this systematic review suggests transgenerational metabolic effects of maternal HFD in rat offspring. We infer that maternal HFD can drive to MetS in offspring by increasing body fat, body weight, and the levels of leptin, by also increasing plasma glucose, insulin, triglycerides concentrations, with the concomitant raise in blood pressure. These findings generally support the fetal origins hypothesis.

## Methods

### Search strategy

Systematic literature search was performed following guidelines outlined in PRISMA (Preferred Reporting Items for Systematic Reviews and Meta-Analyses) statement. We searched for published studies on Pubmed database and additionally obtained the citations of relevant articles by reviewing the references of retrieved studies and review articles. The literature search was done on articles published up to 30 June, 2016. Key MeSH terms used in the search strategy include: high fat, high-fat, lard-fed, fat-rich, mothers, maternal, pregnancy, gestation, rat and offspring. See Supplementary Table S4 for complete search strategy. Searches were restricted to studies on rats that were published in English. After screening of titles and abstracts, two reviewers independently examined full text articles. Disagreements were resolved in consensus discussions.

### Inclusion and Exclusion Criteria

Retrieved articles were screened to identify experimental studies on rats where dams were subjected to a HFD around gestation time and phenotypes were measured in offspring older than 30 days of life. One of the following eight phenotypes should have been reported in offspring: body fat, body weight, leptin, glucose, insulin, HDL-c, triglycerides or SBP. We further screened articles using the following inclusion criteria: (1) the study used healthy wild-type laboratory rats: Wistar, Sprague Dawley or Long Evans (obese, diabetic or hypertensive rats were excluded); (2) experimental dams were fed HFD before and/or during whole or any part of pregnancy and a control group was available where dams were fed standard diet; (3) dams and offspring were only subjected to nutritional manipulation (no surgery, drugs, stress, exercise and so on used); and (4) offspring of both control and HFD-fed dams received control diet after maternal dietary intervention. Experiments include a control group of dams on a standard diet consisting in standard chow or any custom-made or home-made diet with normal fat content. HFD consisted of commercial HFD, custom-made or chow-based HFD; based on animal or vegetable fats. Additional exclusion criteria were applied: (1) dams or offspring were subjected to preference tests; (2) parenteral nutrition or any other than natural feeding (force-feeding by gavage); (3) repeated administration of vehicle (although one injection with saline was allowed); (4) normolipidemic diets and comparisons of groups fed diets differing only in qualitative changes in fat content; (5) cafeteria diets or junk food diets (chow or HFD supplemented with obesogenic food items), or other specific diets based on e.g. diet supplements; and (6) animals fed the HFD and also provided with water containing fructose. We excluded the so called cafeteria diets or junk food diets because, in general, it is not easy to quantify the exact ratios of macronutrients eaten by the animals.

### Data Collection

Two review authors independently collected data on study characteristics, quality and results using a standardized data collection form. The detailed technical description of the coding of extracted data and parameters are presented in Supplementary Table S5. Differences were solved by mutual agreement, if needed. We extracted the mean, standard deviation (SD) and sample size for the control and experimental groups for each outcome. Studies that reported results as mean and standard error, and number of animals per group, were also used for meta-analysis. When data were provided in 2D bar plots instead of in a table or text, we extracted the values using WebPlotDigitizer Version 3.10. Data from XY plots were not extracted. We also gathered information on several moderator variables to explain potential heterogeneity in the data: rat strain, offspring sex, age at testing, maternal diet composition (macronutrient composition and caloric density), litter size, timing and duration of maternal dietary manipulation, maternal age at mating or conception, maternal weight during gestation, and birthweight. Finally, information on author’s names, publication year and journal, male genitor diet, maternal food availability, methods of measurement, and any other potentially relevant information, was also recorded. In studies with multiple experimental and control groups, we only extracted data for the pairs of experimental and control groups that matched our inclusion criteria. When outcomes were measured at several time points, we extracted only the last measure of outcome at each different “stage of life” (off_age_stage, see Supplementary Table S5).

### Selection of included studies

**Figure 10:**
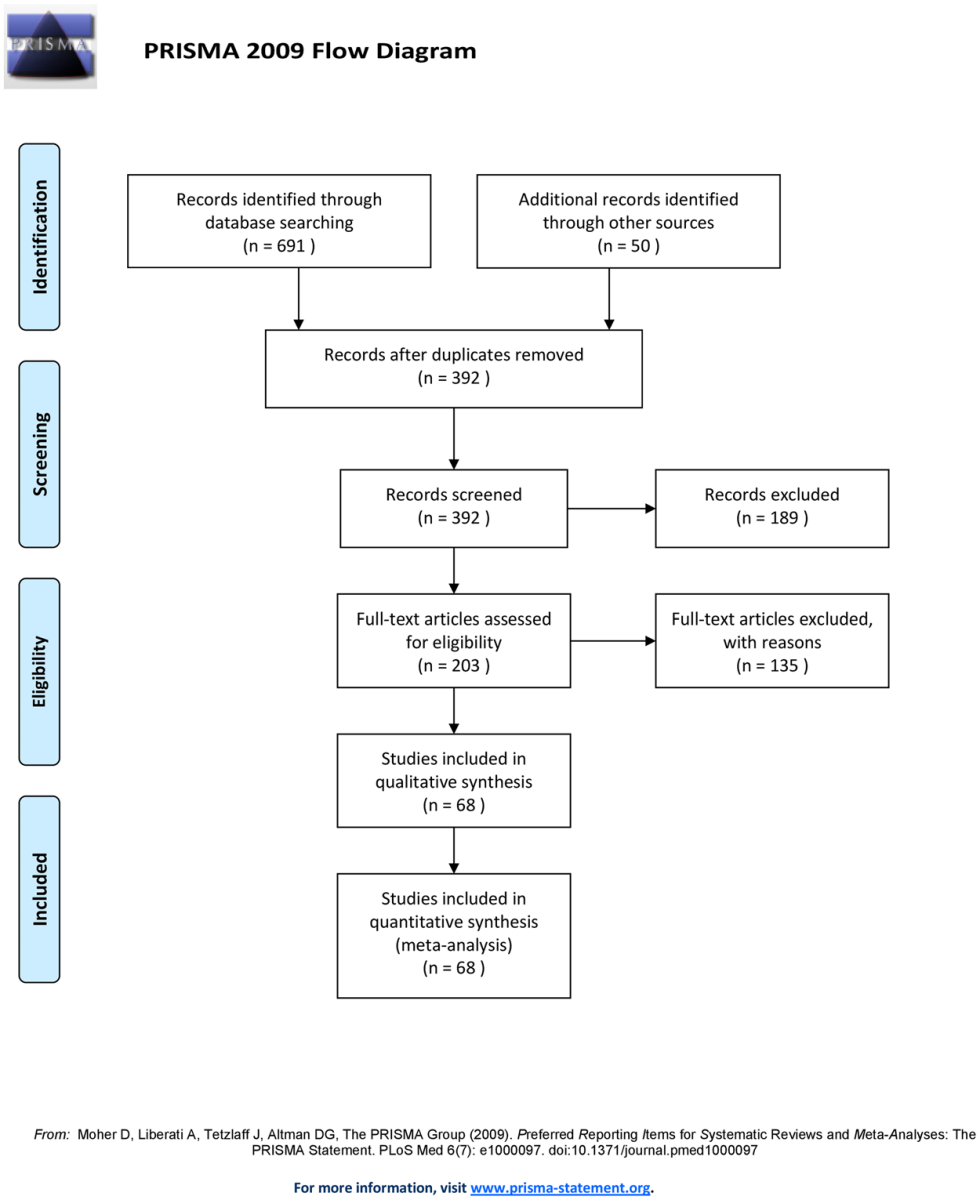
PRISMA flow chart summarizing study selection processes.

The Pubmed search strategy resulted in 380 hits. Reviews and experimental papers were used to perform further searches resulting in approximately additional 50 records. Literature search is summarized in the PRISMA diagram presented in Fig. 10. The initial screen was based on the paper’s title, abstract and occasional whole-text scan and then after in-depth screening, 203 relevant citations remained for further review. Finally, 68 citations were used in meta-analysis^13–17, 19–21, 23, 26, 38, 43, 44, 47–101^. The 135 excluded studies with the reasons for their exclusion are available in Supplementary Table S6.

### Assessment of methodological quality

Methodological quality was assessed based on statements of 1) random allocation into treatment and control groups, 2) husbandry conditions, 3) compliance with animal welfare regulations, and 4) potential conflicts of interests, and whether the study appeared in a peer-reviewed publication^102^. Each article was assessed independently by two reviewers and scored on a scale from 0 to 5 points.

### Data Analysis

#### Extended data set

We performed the analyses independently for the eight outcomes which were extracted or calculated from the collected data. For each outcome, effect size stands for Cohen’s standardized mean difference (D), which was the difference of means between groups (experimental vs. control) divided by the common within-group SD. Where a standard error was presented, the value was converted to a standard deviation. Forest plots were generated to illustrate the study-specific effect sizes along with 95% CI. While the fixed-effect model was applied in the absence of heterogeneity, in general we used a random-effect model. To test robustness of the estimates, we performed sensitivity analyses by omitting one study at a time and calculating the pooled effect size for the remainder of the studies. Meta-regression was used to uncover the potential influence of moderators. These moderators were: age of offspring at testing, maternal age at mating/conception, starting time of manipulation, litter size, increase in energy from fat in experimental diet with respect to control diet, and protein-to-non protein ratio in experimental diet.

Heterogeneity was evaluated with the Q statistic and I-squared statistic. Subgroup analyses were conducted to examine possible sources of heterogeneity. We ran a separate analysis for each level of rat strain, offspring’s sex, offspring’s age, maternal age, timing of maternal diet manipulation, maternal weight, and birthweight. Subgroup analyses were further conducted on extended dataset for the type of fat source in the experimental diet. To check for publication bias we used the Egger’s test and visual inspection of funnel plots for the presence of data distribution asymmetry.

#### Main data set

We created a main data set including only the experiments where lard-based diets were fed to the dams, where we had reliable information on diet caloric density and macronutrient composition, where energy from fat were between 40 and 60% energy and the decrease in protein content was up to 10% energy in the experimental diet with respect to the control diet. We excluded those studies with male genitor HFD and restrictions in maternal food availability. The main data set was processed and analyzed in the same manner as the extended data set. Further subgroup analyses were conducted for the type of experimental diet in main dataset.

Calculations were performed using the Comprehensive Meta-Analysis computer program (Biostat, Englewood, NJ, USA). Between groups comparisons of energy from fat and protein-to-non protein ratio were performed by using ANOVA (CSS/Statistica program package, StatSoft V 6.0, Tulsa, USA). A p-value < 0.05 was considered to be statistically significant.

## Electronic supplementary material

Supplementary Information
