# Supplementary material for: The Association between High Fat Diet around Gestation and Metabolic Syndrome-related Phenotypes in Rats: A Systematic Review and Meta-Analysis
Source: Sci Rep. 2017 Jul 11;7:5086. doi: 10.1038/s41598-017-05344-7 (PMC5506021; doi:10.1038/s41598-017-05344-7)

SUPPLEMENTARY INFORMATION

for

The Association Between High Fat Diet around gestation and Metabolic Syndrome-related Phenotypes in Rats: A Systematic Review and Meta-Analysis

Mariana L Tellechea, PhD; Melisa F Mensegue, MD; Carlos J Pirola, PhD.

Table S1: Summary of the included data (extended dataset), page 2

Table S2: Characteristics of the experimental diets, page 3

Table S3: Assessment of methodological quality, page 5

Table S4: Search Strategy for Pubmed, page 7

Table S5: Detailed technical description of the coding of extracted data and parameters, page 8

Table S6: List of excluded studies with reasons for exclusion, page 9

Figure S1: Timing of maternal dietary manipulations, page 12

Figure S2: Energy from fat and protein in experimental and control diets, page 14

Figure S3: Forest Plots for data on main dataset, page 16

Figure S4: Subgroup analyses for HDL-c, extended dataset, page 21

Figure S5: Subgroup analyses for main dataset, page 21

Figure S6: Meta-regression uncovering the potential influence of moderators, page 23

Figure S7: Funnel plots for the presence of data distribution asymmetry, page 30

Supplementary Table S1: Summary of the included data (extended dataset).

The data is split into eight subsets determined by outcome. N cohorts: total number of experimental groups identified for the purposes of our analysis. N data points: number of effect sizes. Abbreviations: N: number, NA: not available, W: Wistar, SD: Sprague Dawley, LE: Long Evans, PPP: Prepubertal/Pubertal, YA: Young Adult and A: Adult.

Outcome		Body weight	Body fat	Leptin	Glucose	Insulin	HDL-c	Triglycerides	SBP
N studies		49	37	26	28	32	11	26	9
N cohorts		105	84	54	62	72	24	55	21
N rats		1589	1131	861	927	1019	316	776	204
N data points		75	63	43	50	52	21	44	14
N (%) data points by sex	male	47 (63)	42 (67)	24 (56)	30 (60)	32 (61)	11 (52)	24 (55)	6 (43)
	female	25 (33)	19 (30)	18 (42)	19 (38)	18 (35)	9 (43)	18 (41)	8 (57)
	both	3 (4)	2 (3)	1 (2)	1 (2)	2 (4)	1 (5)	1 (2)	0 (0)
	NA	0 (0)	0 (0)	0 (0)	0 (0)	0 (0)	0 (0)	1 (2)	0 (0)
N (%) data points by strain	W	35 (47)	27 (43)	24 (56)	21 (42)	21 (40)	8 (38)	17 (39)	0 (0)
	SD	36 (48)	34 (54)	19 (44)	26 (52)	30 (58)	13 (62)	27 (61)	14 (100)
	LE	4 (5)	2 (3)	0 (0)	3 (6)	1 (2)	0 (0)	0 (0)	0 (0)
N (%) data points by age stage	PPP	11 (14)	12 (19)	5 (12)	5 (10)	7 (13)	2 (10)	7 (16)	4 (29)
	YA	56 (75)	43 (68)	32 (74)	34 (68)	35 (67)	12 (57)	27 (61)	7 (50)
	A	8 (11)	8 (13)	6 (14)	11 (22)	10 (19)	7 (33)	10 (23)	3 (21)

Supplementary Table S2: Characteristics of the experimental diets of the studies included in extended dataset.

The data are grouped by main fat source and fat type: 1) animal (lard, butter, milk fat, mainly ω-9 saturated and monounsaturated fatty acids), 2) mixed and 3) vegetal (high in saturated fatty acids: e.g. coconut and palm oils, monounsaturated: e.g. canola oil, and polyunsaturated: e.g. ω-6 oils like safflower and corn oils, or ω-3 oils like soybean oil). Abbreviations: comm: commercial, NA: not available. When the composition in %kcal was not available, it was calculated from the available data in %w/w or it was taken from data declared by manufacturer.

Reference	fat_source	fat_exp (%kcal)	fat_ exp-con (%kcal)	carb_ exp-con (%kcal)	protein_ exp-con (%kcal)	energy_exp (Kcal/g)	energy _exp-con (Kcal/g)	diet_type
Burgueño AL 2013	lard	68.0	48.8	-49	-22.3	5.8	2.8	chow based
Srinivasan M 2006	lard	60.3	48.5	-51.4	-5.8	5.3	2.1	comm.
Tamashiro KL 2009	lard	60	43	-39.3	-3.2	5.2	2.1	comm.
Sun B 2012<br>Sun B 2013	lard	60	46.5	-36.6	-9.8	5.2	1.9	comm.
White CL, Bruce-Keller AJ 2009<br>White CL, Morrison CD 2009	lard	60	50	-50	0	5.2	1.4	comm.
Sasaki A 2013	lard	60	46.5	-38	-8.5	5.2	2.2	comm.
Marco A 2014	lard	60	42.6	-39.3	-3.2	5.2	2.1	comm.
Lecoutre S 2016	lard	60	50	-50	0	5.2	1.4	comm.
Ambrosetti V 2016	lard	60	47					comm.
Guberman C 2013	lard	60	50	-50	0	5.2	1.4	comm.
Seet EL, Desai M 2015<br>Desai M, Ross MG 2014<br>Desai M, Ross MG 2015	lard	60	50	-50	0	5.1	1.4	comm.
Walker CD, Azeddine B 2008<br>Naef L, Walker CD 2008	lard	59.5	46.4	-48.4	-4.2	4.5	1.1	custom-made
Koukkou E 1998	lard	53.8	42.8	-30.3	-12.5	5.0	1.7	comm.
Mendes-da-Silva C 2014	lard	52	37.3	-34.3	-5.9	4.1	1.3	chow based
Taylor PD, Poston L 2005<br>Khan IY, Poston L 2003	lard	48.8	35.6	-28.6	-7.0	4.7	1.1	chow based
Khan IY 2004	lard	48.7	35.6	-28.6	-7.0	4.7	1.1	chow based
Khan IY 2005	lard	51.6	37.2	-28.6	-8.3	4.5	1.2	chow based
Armitage JA 2005	lard	47.4	33.7	-26.4	-7.4	4.6	1.1	chow based
Eleftheriades M 2014	lard	46.7	34.5	-35.3	-15.5	4.4	1.6	chow based
Vega CC, Zambrano E 2015<br>Bautista CJ, Zambrano E 2016<br>Rodríguez-González GL, Zambrano E 2015<br>Zambrano E, Nathanielsz PW 2010<br>Santos M, Zambrano E 2015	lard	45.9	34.7	-27.7	-2.8	4.9	0.9	NA
Zhang X 2011	lard	45.9	30.2	-29.1	0.4	4.7	0.9	comm.
Page KC 2009	lard	45.7	35.6	-35.2	0.3	4.7	0.9	comm.
Howie GJ 2009<br>Howie GJ 2013	lard	45.7	28.2	-24.7	-2.9	4.7	1.6	comm.
Smith T 2014	lard	45.7	32.4	-19.4	-4.3	4.7	1.3	comm.
Pereira TJ 2015	lard	45	35	-35.2	0.3	4.7	0.9	comm.
Cordero P 2015	lard	45	32	-32	0	4.7	1.8	comm.

Sloboda DM 2009	lard	45	31.8	-19.4	-1.2	4.7	1.3	comm.
Tsoulis MW 2016	lard	45	31	-19.8	-11.7	4.7	1.7	comm.
Gray C, Reynolds CM 2015	lard	45	35.0	-35.2	0.3	4.7	0,9	comm.l
Reynolds CM 2015	lard	45	35	-35	0	4.7	0.9	comm.
Pileggi CA 2016	lard	45	35	-35	0	4.7	0.9	comm.
Song Y 2015	lard	45	35.0	-35.2	0.3	4,7	0.9	comm.
Latouche C 2014	lard	45	28.7	-28.6	-0.4	4.7	0.8	comm.
Yang KF 2012	lard	45	29	-29	0	4.0	0	NA
Ghosh P 2001	lard	43.3	32.3	-22.8	-9.5	4.2	0.9	comm.
Miotto PM, Ward WE 2013 MacPherson RE, Ward WE, Peters SJ 2015	lard	41	23.8	-24	0.3	4.4	0.6	custom-made
Hanafi MY 2016	lard	13.0	2.8	-16.0	-2.4	4.7	0.9	NA
Mazzucco MB 2016	butter	48.5	35.7	-25.3	-10.4	4.8	1.3	chow based
Adamu HA 2016	corn oil, cream milk	51.4	38.5	-36.1	-12.1	5.5	2.1	chow based
Trottier G 1998	soybean oil, shortening	42.2	29.2	-19.3	-3.3	4.3	0.8	custom-made
Kozak R 2000	margarine	73.7	42.5	-39.1	-3.4	5.5	1.2	custom-made (in the lab)
Couvreur O 2011	palm oil	64.5	53.4	-43.6	-9.8	5.7	2.0	custom-made
Férézou-Viala J 2007	palm oil	64.5	52.6	-45.9	-6.7	5.7	1.9	custom-made
Hellgren LI, Lauritzen L 2014	palm oil	48.5	33.2	-20.4	-12.8	4.2	1.3	chow based
Gregersen S, Hermansen K 2005 Dyrskog SE, Hermansen K 2005	coconut oil	58.5	51.0	-51.9	0.9	4.7	1.0	custom-made
Dong YM 2011 cohort a	coconut oil	39.8	24.4	-21.5	-2.9	4.8	0.6	custom-made
Dong YM 2010 cohort b	soybean oil	37.9	22.5	-19.8	-2.7	4.7	0.6	custom-made
Burckley AJ 2005	safflower oil	59	47	45	2	NA	NA	custom-made
Chen H 2012 Chen H 2014	hydrogenated vegetable oil, canola oil	43	29	-25	-4	4.8	2.1	comm.
Rajia S 2013	hydrogenated vegetable oil, canola oil	NA	NA	NA	NA	NA	NA	comm.
Xue Q 2015	NA	60.6	48.3	-42.3	-5.4	5.2	2.0	NA
Sun B 2014	NA	50.5	37.5	-29.7	-7.8	4.3	1.3	custom-made (in the lab)
Gray C, Vickens MH 2015	NA	45	35	NA	NA	NA	NA	comm.
Zaborska KE 2016	NA	45	35	NA	NA	NA	NA	comm.
Hou M 2015	NA	28.8	18.0	-16.1	-1.9	4.7	0.5	comm.

Supplementary Table S3: Assessment of methodological quality.

Methodological quality was assessed based on statements of 1) random allocation into treatment and control groups, 2) husbandry conditions (e.g., light/dark cycle, temperature, access to water, and environmental enrichment), 3) compliance with animal welfare regulations, and 4) potential conflicts of interests, and whether the study appeared in a peer-reviewed publication. Each article was assessed independently by two reviewers and scored on a scale from 0 to 5 points.

Reference	Statement of				Peer reviewed	Score
	Random allocation to experimental and control diets	Husbandry conditions	Compliance with animal welfare regulations	Potential conflict of interests		
Burgueño AL 2013	1	1	1	1	1	5
Srinivasan M 2006	1	1	1	0	1	4
Tamashiro KL 2009	0	1	1	1	1	4
Sun B 2012	0	1	1	1	1	4
Sun B 2013	0	1	1	1	1	4
White CL, Bruce-Keller AJ 2009	0	1	1	0	1	3
White CL, Morrison CD 2009	0	1	1	0	1	3
Sasaki A 2013	0	1	1	0	1	3
Marco A 2014	1	1	1	0	1	4
Lecoutre S 2016	0	1	1	1	1	4
Ambrosetti V 2016	0	1	1	1	1	4
Guberman C 2013	0	1	1	1	1	4
Seet EL 2015	1	1	1	1	1	5
Desai M 2014	0	1	1	1	1	4
Desai M 2015	0	1	1	1	1	4
Walker CD 2008	0	0	0	1	1	2
Naef L 2008	0	1	1	1	1	4
Koukkou E 1998	0	0	0	0	1	1
Mendes-da-Silva C 2014	1	1	1	1	1	5
Taylor PD 2005	0	1	1	0	1	3
Khan IY 2003	0	0	0	0	1	1
Khan IY 2005	0	1	0	0	1	2
Khan IY 2004	0	1	1	0	1	3
Armitage JA 2005	0	1	1	0	1	3
Eleftheriades M 2014	1	1	1	1	1	5
Vega CC 2015	1	1	1	1	1	5
Bautista CJ 2016	1	1	1	1	1	5
Rodríguez-González GL 2015	1	1	0	1	1	4
Zambrano E 2010	1	1	1	0	1	4
Santos M 2015	1	1	1	1	1	5
Zhang X 2011	0	1	1	1	1	4
Page KC 2009	0	1	1	0	1	3
Howie GJ 2009	0	1	1	0	1	3
Howie GJ 2013	0	1	1	1	1	4
Smith T 2014	0	1	1	1	1	4
Pereira TJ 2015	1	1	1	1	1	5
Cordero P 2015	0	1	1	0	1	3
Sloboda DM 2009	0	1	1	1	1	4
Tsoulis MW 2016	1	1	1	0	1	4
Gray C, Reynolds CM 2015	1	1	1	1	1	5
Reynolds CM 2015	1	1	1	0	1	4
Pileggi CA 2016	1	1	1	1	1	5
Song Y 2015	0	1	1	1	1	4
Latouche C 2014	0	1	1	1	1	4

Yang KF 2012	1	1	1	0	1	4
Ghosh P 2001	0	1	1	0	1	3
Miotto PM 2013	1	1	1	1	1	5
MacPherson RE 2015	0	1	1	1	1	4
Hanafi MY 2016	1	1	1	1	1	5
Kozak R 2000	1	1	1	0	1	4
Mazzucco MB 2016	0	1	1	0	1	3
Adamu HA 2016	0	1	1	1	1	4
Trottier G 1998	0	1	1	0	1	3
Couvreur O 2011	1	1	1	1	1	5
Férézou-Viala J 2007	0	1	1	0	1	3
Hellgren LI 2014	0	1	1	1	1	4
Gregersen S 2005	0	1	1	0	1	3
Dyrskog SE 2005	0	1	1	0	1	3
Dong YM 2011	0	1	1	1	1	4
Burckley AJ 2005	1	1	1	0	1	4
Chen H 2012	0	1	1	1	1	4
Rajia S 2013	0	1	1	1	1	4
Chen H 2014	0	1	1	1	1	4
Xue Q 2015	1	1	1	1	1	5
Sun B 2014	0	1	1	1	1	4
Gray C, Vickens MH 2015	1	1	1	1	1	5
Zaborska KE 2016	0	1	1	1	1	4
Hou M 2015	1	1	1	1	1	5

Supplementary Table S4: Search Strategy for Pubmed (last conducted June 30, 2016).

Key MeSH terms used in the search strategy include: high fat, high-fat, lard-fed, fat-rich, mothers, maternal, pregnancy, gestation, rat, offspring.

Query	Items found
HIGH-FAT[All Fields] AND ("mothers"[MeSH Terms] OR "mothers"[All Fields] OR "maternal"[All Fields]) AND ("rats"[MeSH Terms] OR "rats"[All Fields] OR "rat"[All Fields]) AND OFFSPRING[All Fields]	312
HIGH-FAT[All Fields] AND ("pregnancy"[MeSH Terms] OR "pregnancy"[All Fields] OR "gestation"[All Fields]) AND ("rats"[MeSH Terms] OR "rats"[All Fields] OR "rat"[All Fields]) AND OFFSPRING[All Fields]	338
LARD-FED[All Fields] AND ("mothers"[MeSH Terms] OR "mothers"[All Fields] OR "maternal"[All Fields]) AND ("rats"[MeSH Terms] OR "rats"[All Fields] OR "rat"[All Fields]) AND OFFSPRING[All Fields]	3
LARD-FED[All Fields] AND ("pregnancy"[MeSH Terms] OR "pregnancy"[All Fields] OR "gestation"[All Fields]) AND ("rats"[MeSH Terms] OR "rats"[All Fields] OR "rat"[All Fields]) AND OFFSPRING[All Fields]	3
FAT-RICH[All Fields] AND ("mothers"[MeSH Terms] OR "mothers"[All Fields] OR "maternal"[All Fields]) AND ("rats"[MeSH Terms] OR "rats"[All Fields] OR "rat"[All Fields]) AND OFFSPRING[All Fields]	16
FAT-RICH[All Fields] AND ("pregnancy"[MeSH Terms] OR "pregnancy"[All Fields] OR "gestation"[All Fields]) AND ("rats"[MeSH Terms] OR "rats"[All Fields] OR "rat"[All Fields]) AND OFFSPRING[All Fields]	19
	691

OTHER SOURCES: forward and backward searches, reviews, experimental papers, author-based searches [50 records].

Supplementary Table S5: Detailed technical description of the coding of extracted data and parameters (modified from Besson AA et al, {Besson, 2016 BESSON2016 /id}).

reference: author's names and date
publish_date: publish date (year)
impact_factor: publication impact factor
strain: strain
fat_exp_kcal: energy from fat in experimental diet (%kcal)
carb_exp_kcal: energy from carbohydrates in experimental diet (%kcal)
protein_exp_kcal: energy from protein in experimental diet (%kcal)
fat_con_kcal: energy from fat in control diet (%kcal)
carb_con_kcal: energy from carbohydrates in control diet (%kcal)
protein_con_kcal: energy from protein in control diet (%kcal)
When the composition in % kcal was not available, it was calculated from the available data in %w/w or it was taken from data declared by manufacturer
fat_exp-con_kcal: increase in energy from fat in experimental diet relative to the control group (%kcal), fat is expressed as a difference relative to the control diet
carb_exp-con_kcal: carbohydrates in experimental diet are expressed as a difference relative to the control diet (%kcal)
protein_exp-con_kcal: protein in experimental is expressed as a difference relative to the control diet (%kcal)
P:NP ratio: protein-to-non protein ratio in experimental diet (%kcal)
energy_exp: metabolic energy of the experimental maternal diet (Kcal/g)
energy_con: metabolic energy of the control maternal diet (Kcal/g)
energy_exp-con: energy in experimental is expressed as a difference relative to the control diet (Kcal/g)
fat_source: fat source of the experimental diet
diet_type: maternal diet type (commercial, custom-made or chow-based, as far as could be judged from the description)
adlib_con: were maternal control groups fed *ad libitum*? (yes or no)
adlib_exp: were maternal experimental groups fed *ad libitum*? (yes or no)
dam_diet_start: start of the maternal diet in days (pre-conception)
diet_duration: duration of intervention from start (pregestation or gestation) to end (birth or weanling) in days.
diet_timing: period of maternal diet manipulation (pre/ges+lac, ges, ges+lac)
age_mating: dam age at mating/conception
dam_age_stage: maternal age stage at mating/conception [2 sub-groups: young adult rats (PND63 to PND209) and adult (aged ≥ 210 days, musculoskeletal maturity)]
random_allocation: random allocation into experimental and control groups (yes or no)
husbandry: husbandry conditions (e.g., breeding programs, light/dark cycle, temperature, type of food, access to water, and environmental enrichment) [yes or no]
animal_welfare: compliance with animal welfare regulations [yes or no]
disclosure: statement of potential conflicts of interests [yes or no]
peer_reviewed: whether the study appeared in a peer-reviewed publication [yes or no]
litter_size: size of the litter (number of pups per dam)
litter_size_equalized: has litter size been equalized? (yes or no)
sex: sex of the offspring (male, female, mixed sex)
offspring_diet: offspring diet after weaning (type of control diet)
birth_weight_sign: was offspring birth weight in the experimental group decreased, increased or not different from control group?
maternal_weight_sign: was maternal weight during gestation in the experimental group increased or not different from control group? Maternal obesity during gestation was assumed if studies reported a significant increase in body weight in comparison to control dams.
male_genitor_diet: was male genitor fed experimental diet? (yes or no)
For each outcome:
unit: unit of the outcome measure taken
exp_mean: mean of the offspring outcome measured for the experimental group
exp_sd: SD of the offspring outcome measured for the experimental group
exp_n: sample size for the offspring experimental group.
con_mean: mean of offspring outcome measured for the control group
con_sd: SD of the offspring outcome measured for the control group
con_n: sample size for the offspring control group
offspring_age: offspring age when outcome was tested [in days post-parturition]
off_age_stage: offspring age stage when outcome was tested [3 sub-groups: pre-pubertal and pubertal rats (< PND63), young adult rats (PND63 to PND209) and adult (aged ≥ 210 days, musculoskeletal maturity)]
data_plot: was data extracted from 2D Bar Plot? (yes or no)
method_measurement

Supplementary Table S6: List of excluded studies with reasons for exclusion.

The initial screen was based on the paper's title, abstract and occasional whole-text scan. We initially excluded studies not published in English, studies on other laboratory animals than rats, studies using no wild-type laboratory rats or rats with a condition or disease (obese, diabetic or hypertensive rats), studies were dams and/or offspring were subjected to other treatments (surgery, drugs, stress, exercise), and studies with maternal manipulations not matching inclusion criteria. Studies on male genitor HFD or maternal HFD during lactation alone were excluded. After initial screening, 203 full text articles were examined, 135 articles were excluded, with reasons:

PubMed ID	Article	Reasons for exclusion
22395468	Epigenetics. 2012 Feb;7(2):183-90.	Data from XY Plots or Whiskers Bar Graphs
26102652	J Hypertens. 2015 Jun;33 Suppl 1:e101.	
25716581	J Nutr Biochem. 2015 May;26(5):433-40.	
26075698	J Matern Fetal Neonatal Med. 2016;29(9):1491-7.	
26643911	J Mol Endocrinol. 2016 Feb;56(2):101-12.	
21187125	Neuroscience. 2011 Mar 10;176:225-36.	
26115784	Chem Biol Interact. 2015 Sep 5;239:118-28.	Missing or incomplete data on offspring outcome
23786443	Stress. 2013 Sep;16(5):540-8.	
12429652	Clin Cancer Res. 2002 Nov;8(11):3601-10.	
23836027	Psychopharmacology (Berl). 2013 Dec;230(4):509-24.	
26738750	J Cereb Blood Flow Metab. 2016 Jan 6.	
16646052	Int J Cancer. 2006 Oct 1;119(7):1537-46.	
19707592	PLoS One. 2009 Aug 25;4(8):e6744.	
24746835	J Nutr Biochem. 2014 Jun;25(6):613-22.	
17255173	Exp Physiol. 2007 May;92(3):583-9.	
22094600	Am J Physiol Gastrointest Liver Physiol. 2012 Jan 15;302(2):G250-9.	
22858203	Nutrition. 2012 Nov-Dec;28(11-12):1165-71.	
24791714	Neuroscience. 2014 Jul 11;272:92-101	Incomplete data (n)
22411006	J Physiol. 2012 May 1;590(9):2167-80.	
18614261	Clin Nutr. 2008 Aug;27(4):623-34.	
15774514	J Physiol. 2005 May 15;565(Pt 1):171-84.	Duplicated data
15326063	Circulation. 2004 Aug 31;110(9):1097-102.	
15194731	J Physiol. 2004 Aug 1;558(Pt 3):943-51.	
20229585	Hepatology. 2010 Jun;51(6):2234-5.	
27004609	Sci Rep. 2016 Mar 23;6:23525.	
24523341	Am J Physiol Regul Integr Comp Physiol. 2014 Apr 15;306(8):R527-37.	
25141872	J Dev Orig Health Dis. 2010 Aug;1(4):245-54.	
27163678	PLoS One. 2016 May 10;11(5):e0155108.	
24498431	PLoS One. 2014 Feb 3;9(2):e88127.	Postnatal experimental diet
19576260	Food Chem Toxicol. 2009 Oct;47(10):2407-18.	
23310925	J Endocrinol Invest. 2012 Dec;35(11):981-6.	
2336034	Metabolism. 1990 May;39(5):468-73.	
20403085	J Neuroendocrinol. 2010 Aug;22(8):905-14.	
25498225	Neuroscience. 2015 Feb 12;286:364-70.	
22564493	Physiol Behav. 2012 Nov 5;107(4):568-75.	
27342757	Mol Nutr Food Res. 2016 Jun 25.	
24498431	PLoS One. 2014 Feb 3;9(2):e88127.	
19576260	Food Chem Toxicol. 2009 Oct;47(10):2407-18.	
25498225	Neuroscience. 2015 Feb 12;286:364-70.	
22564493	Physiol Behav. 2012 Nov 5;107(4):568-75.	
2336034	Metabolism. 1990 May;39(5):468-73.	
27342757	Mol Nutr Food Res. 2016 Jun 25.	

25801629<br>24570028<br>24337156<br>22548153	Eur J Nutr. 2016 Mar;55(2):601-10.<br>Eur J Nutr. 2014 Dec;53(8):1669-83.<br>Diabetologia. 2014 Mar;57(3):614-22.<br>J Nutr Metab. 2012;2012:296935.	Administration of vehicle
24416203<br>21901160<br>25336449<br>24450870<br>18032473<br>21862610	PLoS One. 2014 Jan 9;9(1):e84209.<br>PLoS One. 2011;6(8):e24068.<br>Physiol Genomics. 2014 Dec 1;46(23):841-50.<br>J Agric Food Chem. 2014 Feb 19;62(7):1590-601.<br>Am J Physiol Regul Integr Comp Physiol. 2008 Feb;294(2):R528-38.<br>Endocrinology. 2011 Nov;152(11):4158-70.	Force-feeding
23310925<br>20403085<br>21980407<br>23701669<br>23928377<br>19606226<br>21208789<br>26545531<br>20494379<br>18986302<br>22160775<br>23255587<br>27179663<br>26718219<br>19282828<br>26195235<br>24249969<br>21427213<br>24727340	J Endocrinol Invest. 2012 Dec;35(11):981-6.<br>J Neuroendocrinol. 2010 Aug;22(8):905-14.<br>PLoS One. 2011;6(9):e25261.<br>J Neuroendocrinol. 2013 Aug;25(8):742-52.<br>Endocrinology. 2013 Oct;154(10):3610-21.<br>PLoS One. 2009 Jul 16;4(7):e6259.<br>Nutr Metab Cardiovasc Dis. 2012 Mar;22(3):269-76.<br>Nutr Hosp. 2015 Oct 1;32(4):1653-8.<br>Metabolism. 2010 Dec;59(12):1701-9.<br>Clin Sci (Lond). 2009 Apr;116(8):669-80.<br>Am J Physiol Renal Physiol. 2012 Mar 15;302(6):F774-83.<br>Am J Physiol Regul Integr Comp Physiol. 2013 Feb 15;304(4):R278-85.<br>Clin Exp Nephrol. 2016 May 14.<br>Behav Brain Res. 2016 Mar 15;301:124-31. (study ends before weaning)<br>Obesity (Silver Spring). 2009 Jul;17(7):1356-62. (study ends before weaning)<br>Eur J Nutr. 2016 Jun;55(4):1741-53. (study ends before weaning)<br>J Clin Biochem Nutr. 2013 Nov;53(3):150-7. (study ends before weaning)<br>FASEB J. 2011 Jul;25(7):2167-79. (preference test)<br>Physiol Behav. 2014 Jun 22;133:14-21. (preference test)	Cafeteria / Western diet<br>Junk food diet<br>High-fat-sucrose diet<br>High-fat-fructose diet
21722163<br>23819696<br>23782871<br>20688090	Clin Exp Pharmacol Physiol. 2011 Sep;38(9):613-22. (chocolate and fructose)<br>Clin Exp Pharmacol Physiol. 2013 Sep;40(9):652-61. (chocolate and fructose)<br>Acta Physiol (Oxf). 2014 Jan;210(1):142-53. (chocolate and fructose)<br>Physiol Behav. 2010 Nov 2;101(4):494-502. (highly palatable liquid diet supplement)	Other specific diets
20044786<br>12378380<br>16815496	Eur J Nutr. 2010 Sep;49(6):337-43.<br>Diabetologia. 2002 Oct;45(10):1397-403.<br>Nutrition. 2006 Jul-Aug;22(7-8):820-9.	Qualitative changes in fat content<br>Normolipidemic diets
24500433<br>19005075<br>15926937<br>26423785<br>22964789<br>22239918	Am J Physiol Regul Integr Comp Physiol. 2014 Apr 1;306(7):R499-509<br>J Neurosci. 2008 Nov 12;28(46):12107-19.<br>Eur J Neurosci. 2005 May;21(10):2887-92.<br>Physiol Behav. 2015 Dec 1;152(Pt A):119-27.<br>Int J Obes (Lond). 2013 Jun;37(6):885-8.<br>Int J Dev Neurosci. 2012 Apr;30(2):75-81.	Preference test<br>(consumption behavior)
25738800<br>25614359	PLoS One. 2015 Mar 4;10(3):e0118586. (exercise)<br>Psychoneuroendocrinology. 2015 Mar;53:82-93. (stress)	Offspring subjected to other treatments

24146946<br>21779332<br><br>25991721<br>25191597<br>21730967<br>21779332<br>26224356<br>18635655<br>27385731<br>1941255<br>4691274<br>15705804<br>20160134<br>22696509<br>24445041<br>24147051<br>26979053<br>19622195<br>19074472<br>22693204<br>22131269<br>24043569<br>20972941<br>26493953<br>25096554<br>26873412<br>25853572<br>20711951<br>17414058<br>20195946<br>21266050<br>16469158<br>9802435<br>27127509<br>22869015<br>26287659<br>18982008<br>7777603<br>21605577<br>23038630<br>26583035<br>25152240<br>26318151<br>23333087<br>24852604<br>16487482<br>26801311<br>12719763<br>10615212<br>25920746	PLoS One. 2013 Oct 17;8(10):e76961.<br>PLoS One. 2011;6(7):e21662.<br>Journal of Developmental Origins of Health and Disease (2011), 2(1), 63–70.<br>Physiol Rep. 2015 May;3(5).<br>J Nutr Sci. 2014 Jan 2;2:e41.<br>Int J Obes (Lond). 2012 May;36(5):744-51.<br>PLoS One. 2011;6(7):e21662.<br>Nutr Metab Cardiovasc Dis. 2015 Sep;25(9):881-8.<br>Endocrinology. 2008 Nov;149(11):5348-56.<br>Am J Physiol Regul Integr Comp Physiol. 2016 Sep 1;311(3):R497-504<br>J Nutr. 1991 Dec;121(12):1917-23.<br>Am J Physiol. 1973 Mar;224(3):596-9.<br>Am J Physiol Regul Integr Comp Physiol. 2005 May;288(5):R1122-8.<br>Endocrinology. 2010 Apr;151(4):1685-94.<br>J Mol Endocrinol. 2012 Jul 25;49(2):107-14.<br>J Nutr Biochem. 2014 Feb;25(2):170-6.<br>PLoS One. 2013 Oct 11;8(10):e77668.<br>Neuroscience. 2016 Jun 2;324:407-19.<br>Br J Nutr. 2009 Dec;102(11):1601-10.<br>J Endocrinol. 2009 Mar;200(3):293-300.<br>Am J Physiol Endocrinol Metab. 2012 Aug 1;303(3):E432-41.<br>FASEB J. 2012 Mar;26(3):1131-41.<br>Metab Brain Dis. 2013 Dec;28(4):721-5.<br>Horm Metab Res. 2010 Dec;42(13):923-9.<br>Physiol Rep. 2015 Oct;3(10). pii: e12588.<br>Physiol Rep. 2014 Aug 5;2(8). pii: e12110.<br>Physiol Behav. 2016 Apr 1;157:237-45.<br>PLoS One. 2015 Apr 8;10(4):e0120980.<br>Horm Metab Res. 2010 Oct;42(11):821-5.<br>Pancreas. 2007 Apr;34(3):347-53.<br>Horm Metab Res. 2010 May;42(5):307-10.<br>Lipids Health Dis. 2011 Jan 25;10:22.<br>Br J Nutr. 2006 Feb;95(2):391-6.<br>Regul Pept. 1998 Sep 25;75-76:397-402.<br>Int J Endocrinol. 2016;2016:5739763.<br>J Physiol. 2012 Nov 1;590(21):5503-18.<br>Int J Dev Neurosci. 2015 Nov;46:67-75.<br>Int J Obes (Lond). 2009 Jan;33(1):115-22.<br>Physiol Behav. 1995 Apr;57(4):681-6.<br>Physiol Behav. 2011 Sep 1;104(3):474-9.<br>Mol Nutr Food Res. 2012 Nov;56(11):1715-28.<br>Nutr Metab (Lond). 2015 Nov 14;12:40.<br>Int J Obes (Lond). 2015 Jan;39(1):7-15.<br>J Nutr Biochem. 2015 Dec;26(12):1448-57.<br>J Nutr Biochem. 2013 Jul;24(7):1340-8.<br>PLoS One. 2014 May 22;9(5):e97838.<br>Biochem Biophys Res Commun. 2006 Apr 7;342(2):452-8.<br>Am J Physiol Heart Circ Physiol. 2016 Mar 15;310(6):H681-92.<br>Exp Physiol. 2003 May;88(3):389-98.<br>Br J Nutr. 1999 May;81(5):395-404.<br>Lipids. 2015 Jun;50(6):605-10.	Study ends before weaning

Supplementary Figure S1: Timing of maternal dietary manipulations in experiments included in the extended dataset.

Data are presented separately for each outcome. Each horizontal line represents the manipulation timing associated with one effect size.

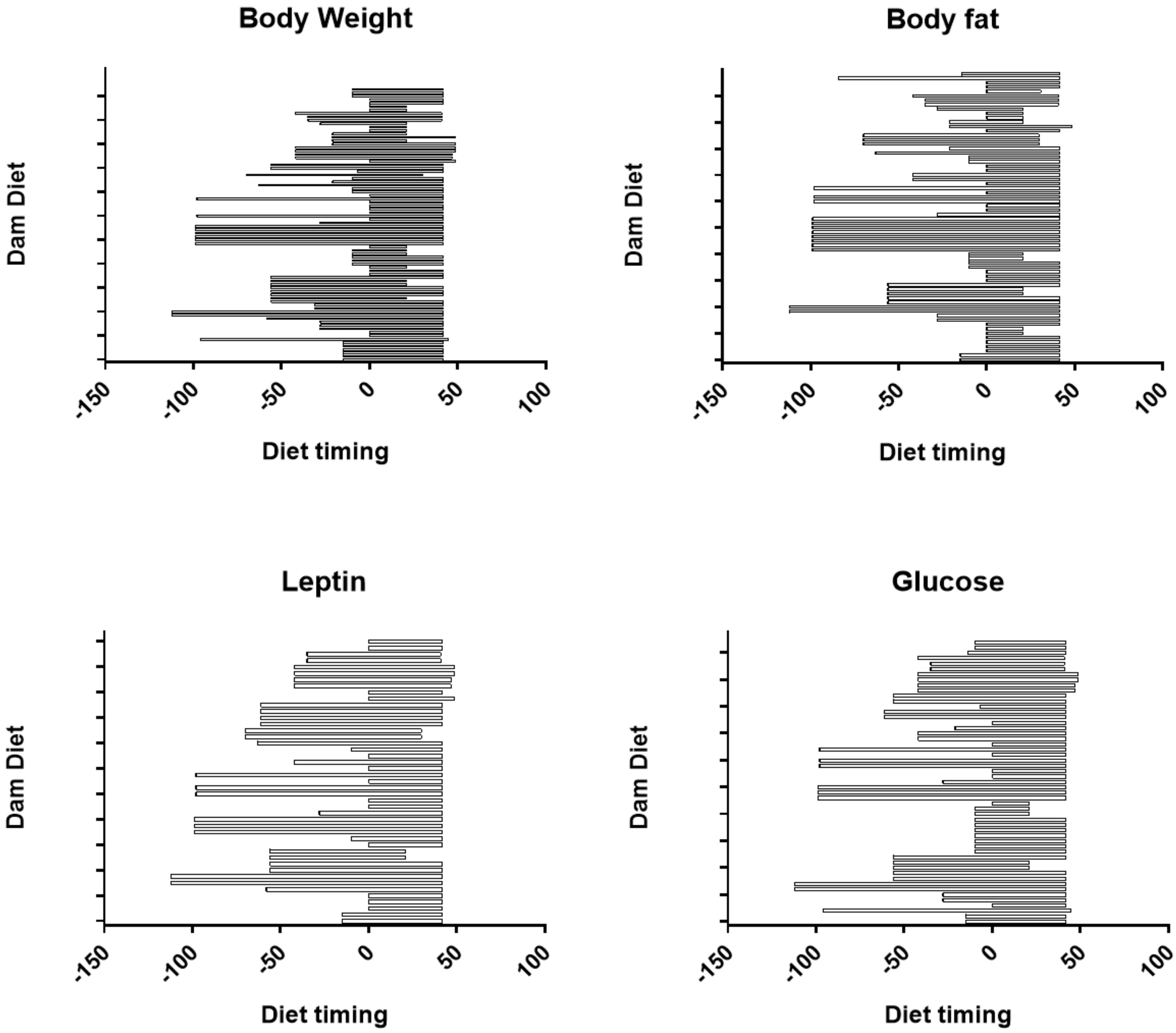

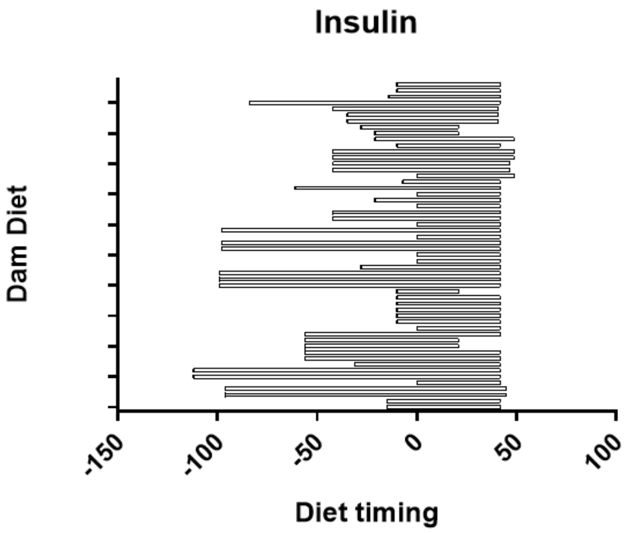
Insulin
Dam Diet
-150
-100
-50
0
50
100
Diet timing

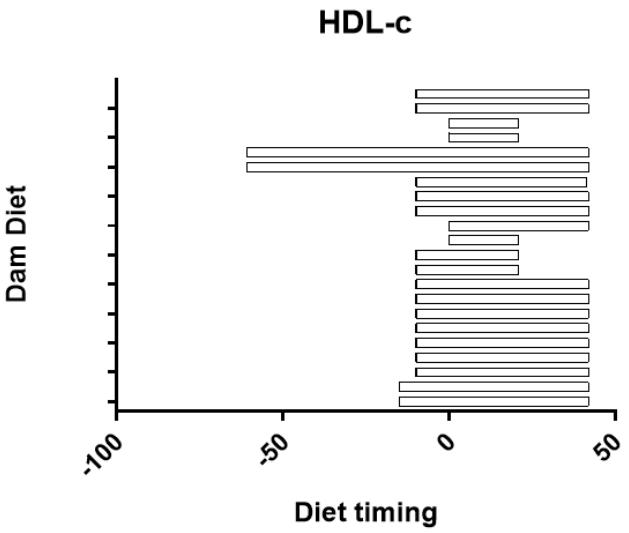
HDL-c
Dam Diet
-100
-50
0
50
Diet timing

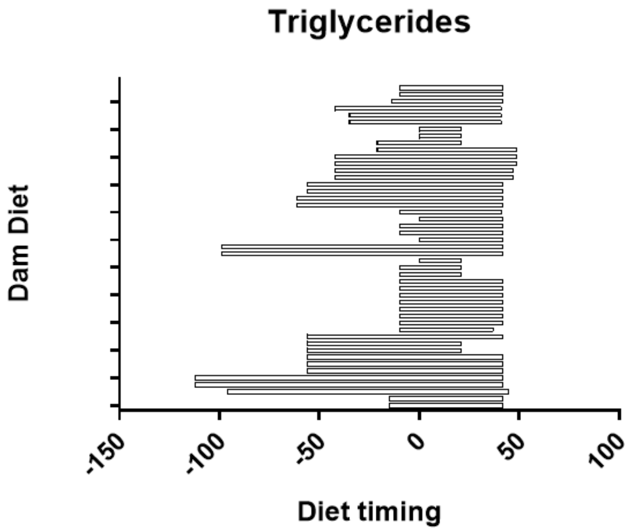
Triglycerides
Dam Diet
-150
-100
-50
0
50
100
Diet timing

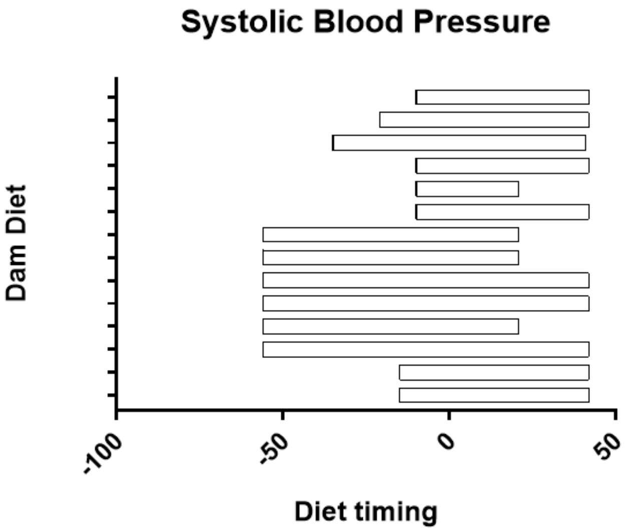
Systolic Blood Pressure
Dam Diet
-100
-50
0
50
Diet timing

Supplementary Figure S2: Energy from fat (%kcal) and protein (%kcal) in experimental and control diets. Data are presented separately for each outcome.

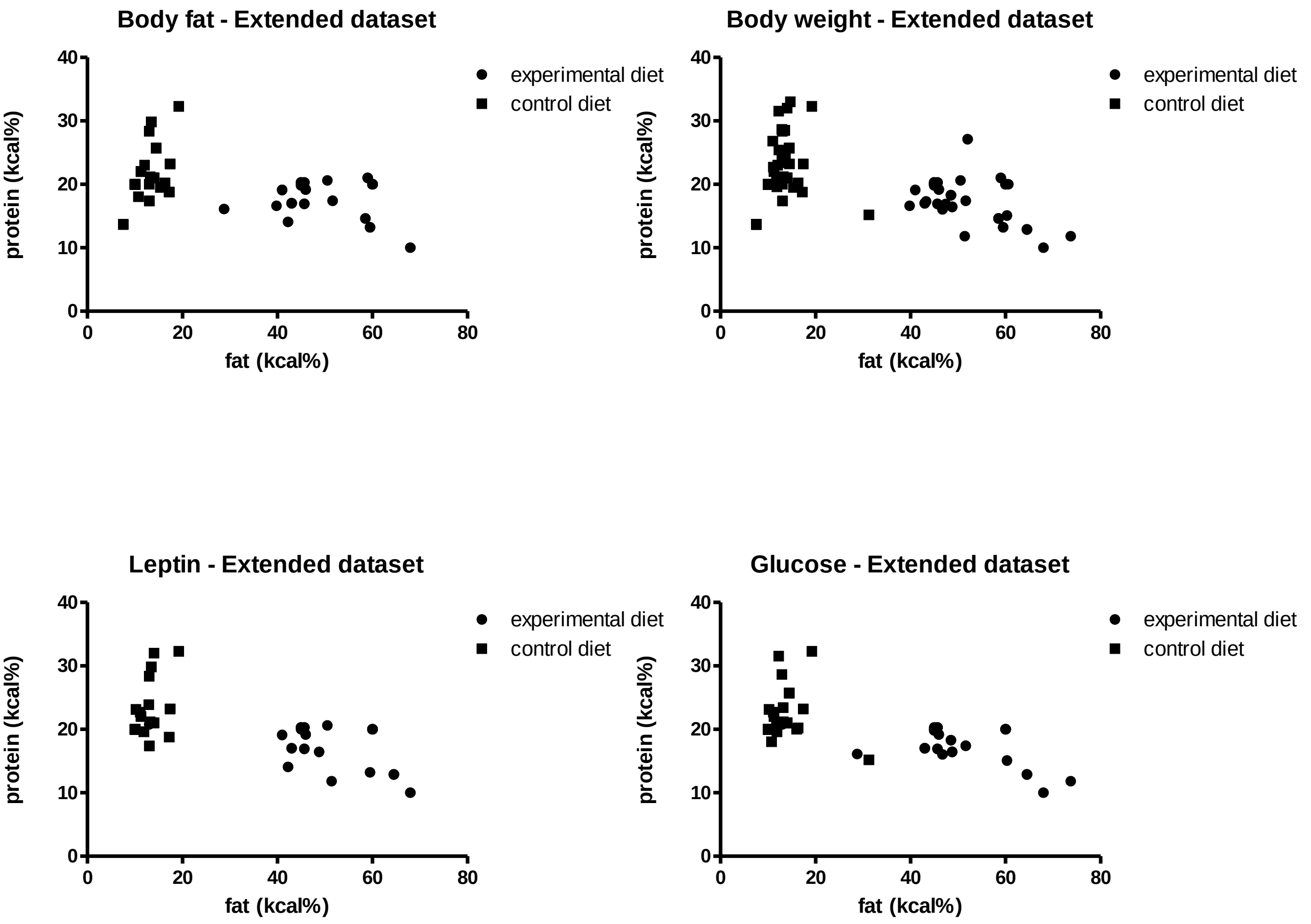

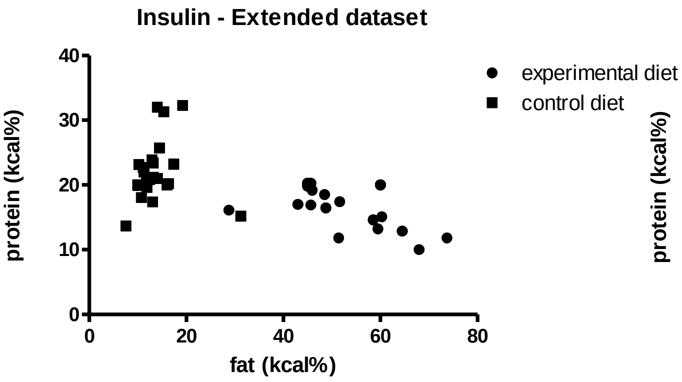
Insulin - Extended dataset
protein (kcal%)
fat (kcal%)
experimental diet
control diet
0
10
20
30
40
0
20
40
60
80

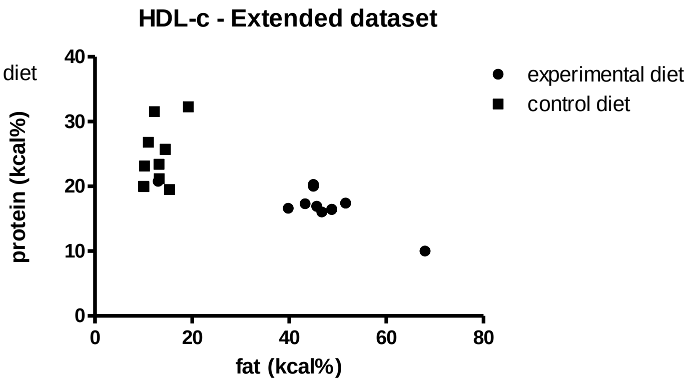
HDL-c - Extended dataset
protein (kcal%)
fat (kcal%)
experimental diet
control diet
0
10
20
30
40
0
20
40
60
80

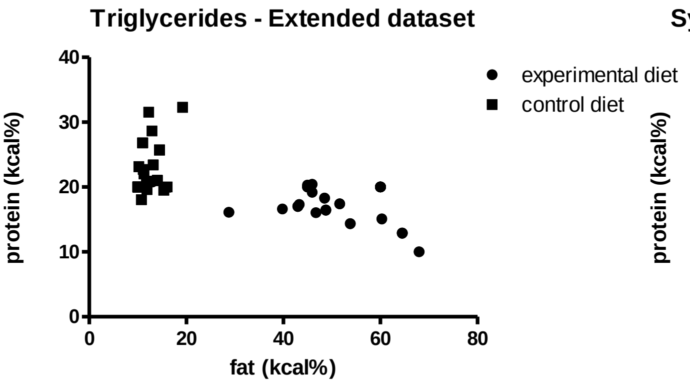
Triglycerides - Extended dataset
protein (kcal%)
fat (kcal%)
experimental diet
control diet
0
10
20
30
40
0
20
40
60
80

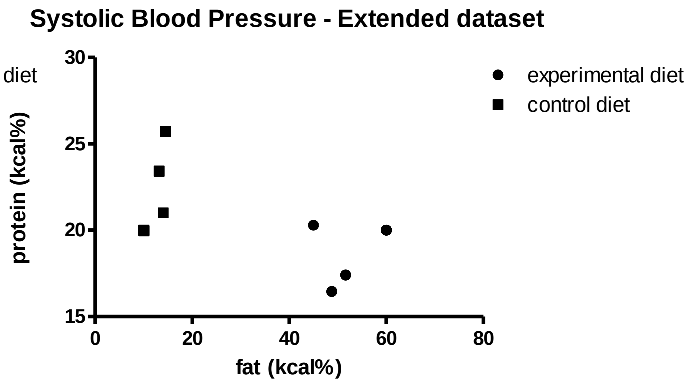
Systolic Blood Pressure - Extended dataset
protein (kcal%)
fat (kcal%)
experimental diet
control diet
15
20
25
30
0
20
40
60
80

Supplementary Figure S3: Forest Plots for data on main dataset.

Summary estimates for standardized difference in means (D) (effect); the corresponding 95 % CI (lower and upper) and significance (p value) were estimated by fixed and random effects analysis. The first author of the study and the year of publication are shown. In the graph, numbers indicate D values, filled squares stand for the effect of individual studies, and filled diamonds express combined fixed and random effects.

Panel A Forest Plot for Body fat

Subgroup within study	Model	Study name	Outcome	Statistics for each study				
				Std diff in means	Standard error	Lower limit	Upper limit	p-Value
main dataset		Tamashiro KL 2009 a	body fat	0.000	0.645	-1.265	1.265	1.000000000
main dataset		Tamashiro KL 2009 b	body fat	0.877	0.702	-0.499	2.253	0.211552673
main dataset		Sun B 2012 a	body fat	0.846	0.522	-0.177	1.868	0.105172055
main dataset		Sun B 2012 b	body fat	1.462	0.563	0.359	2.565	0.009392994
main dataset		Sun B 2012 c	body fat	-0.179	0.501	-1.161	0.803	0.721309213
main dataset		Sun B 2012 d	body fat	-0.500	0.508	-1.495	0.495	0.324755765
main dataset		Sun B 2013 b	body fat	3.319	0.890	1.575	5.064	0.000192410
main dataset		White CL, Morrison CD 2009 a	body fat	1.075	0.338	0.412	1.738	0.001481881
main dataset		Lecoutre S 2016 a	body fat	0.967	0.374	0.235	1.700	0.009638945
main dataset		Lecoutre S 2016 b	body fat	-0.105	0.354	-0.799	0.588	0.765912290
main dataset		Desai M 2014 b	body fat	3.029	0.733	1.593	4.465	0.000035578
main dataset		Desai M 2014 d	body fat	2.268	0.641	1.012	3.524	0.000402046
main dataset		Desai M 2014 f	body fat	2.542	0.672	1.224	3.859	0.000156174
main dataset		Desai M 2014 h	body fat	1.441	0.561	0.341	2.541	0.010222565
main dataset		Desai M 2015	body fat	3.165	0.866	1.467	4.864	0.000259176
main dataset		Walker CD 2008 a	body fat	1.021	0.804	-0.554	2.596	0.203873273
main dataset		Walker CD 2008 b	body fat	0.554	0.770	-0.956	2.063	0.472230779
main dataset		Naef L 2008	body fat	0.566	0.496	-0.405	1.538	0.253059463
main dataset		Khan IY 2005 a	body fat	1.328	0.494	0.359	2.296	0.007198498
main dataset		Khan IY 2005 b	body fat	1.429	0.501	0.447	2.412	0.004335589
main dataset		Khan IY 2005 d	body fat	1.890	0.569	0.774	3.006	0.000901627
main dataset		Khan IY 2005 e	body fat	0.909	0.498	-0.067	1.885	0.067928589
main dataset		Vega CC 2015 a	body fat	1.024	0.532	-0.018	2.067	0.054066228
main dataset		Vega CC 2015 b	body fat	1.410	0.559	0.315	2.505	0.011595568
main dataset		Bautista CJ 2016 a	body fat	1.387	0.704	0.006	2.767	0.048982027
main dataset		Bautista CJ 2016 b	body fat	1.916	0.764	0.419	3.413	0.012146846
main dataset		Rodríguez-González GL 2015 a	body fat	3.561	0.969	1.660	5.461	0.000240021
main dataset		Rodríguez-González GL 2015 b	body fat	1.787	0.713	0.389	3.185	0.012225694
main dataset		Zambrano E 2010	body fat	4.531	1.194	2.190	6.872	0.000148439
main dataset		Santos M 2015	body fat	2.920	0.909	1.138	4.701	0.001317443
main dataset		Page KC 2009	body fat	2.934	0.588	1.781	4.087	0.000000611
main dataset		Howie GJ 2009 a	body fat	2.875	0.672	1.557	4.192	0.000018938
main dataset		Howie GJ 2009 b	body fat	2.091	0.586	0.942	3.240	0.000361450
main dataset		Howie GJ 2009 c	body fat	2.836	0.668	1.527	4.144	0.000021549
main dataset		Howie GJ 2009 d	body fat	1.796	0.558	0.701	2.890	0.001300426
main dataset		Smith T 2014	body fat	3.326	0.690	1.973	4.679	0.000001450
main dataset		Pereira TJ 2015 a	body fat	2.746	0.805	1.169	4.323	0.000643715
main dataset		Pereira TJ 2015 b	body fat	-0.639	0.592	-1.799	0.521	0.280159271
main dataset		Cordero P 2015 a	body fat	1.142	0.598	-0.030	2.314	0.056251931
main dataset		Cordero P 2015 b	body fat	0.215	0.556	-0.875	1.305	0.699313657
main dataset		Gray C, Reynolds CM 2015	body fat	2.309	0.645	1.044	3.574	0.000347477
main dataset		Reynolds CM 2015	body fat	0.804	0.600	-0.372	1.981	0.180277843
main dataset		Song Y 2015	body fat	0.734	0.462	-0.172	1.639	0.112279149
main dataset		Latouche C 2014	body fat	1.416	0.674	0.094	2.737	0.035776733
main dataset		Miotto PM 2013 a	body fat	0.157	0.472	-0.769	1.082	0.739859459
main dataset		Miotto PM 2013 b	body fat	-0.050	0.471	-0.974	0.874	0.914849125
main dataset		MacPherson RE 2015	body fat	0.355	0.336	-0.304	1.013	0.290943124
	Fixed			1.141	0.082	0.980	1.301	0.000000000
	Random			1.383	0.156	1.077	1.689	0.000000000

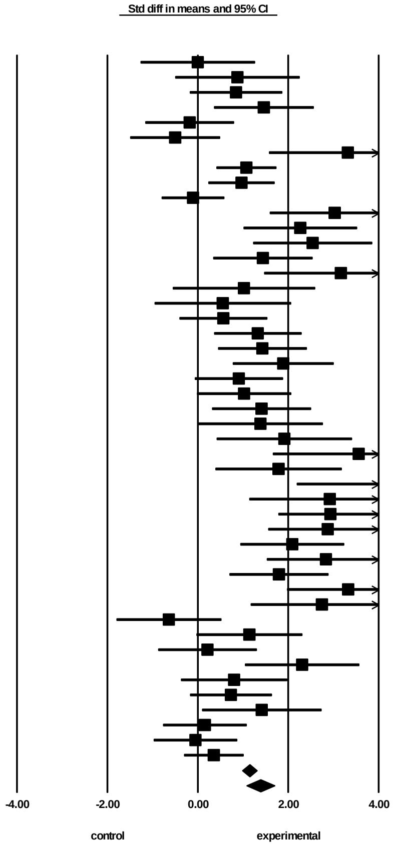

Panel B Forest Plot for Body weight

Subgroup within study	Model	Study name	Outcome	Std diff in means	Standard error	Lower limit	Upper limit	p-Value
main dataset		Srinivasan M 2006 b	body weight	2.013	0.579	0.879	3.147	0.000503394
main dataset		Tamashiro KL 2009 a	body weight	0.307	0.649	-0.965	1.579	0.636374493
main dataset		Tamashiro KL 2009 b	body weight	-0.370	0.676	-1.696	0.956	0.584497297
main dataset		White CL, Bruce-Keller AJ 2009	body weight	1.213	0.493	0.246	2.179	0.013897738
main dataset		Sasaki A 2013 a	body weight	0.668	0.414	-0.143	1.479	0.106660902
main dataset		Sasaki A 2013 b	body weight	0.491	0.399	-0.291	1.274	0.218449228
main dataset		Marco A 2014	body weight	1.050	0.477	0.115	1.985	0.027768957
main dataset		Lecoutre S 2016 a	body weight	0.433	0.358	-0.268	1.134	0.225663521
main dataset		Lecoutre S 2016 b	body weight	-0.275	0.355	-0.971	0.421	0.438902698
main dataset		Ambrosetti V 2016 a	body weight	2.257	0.653	0.978	3.536	0.000542316
main dataset		Ambrosetti V 2016 b	body weight	1.882	0.647	0.614	3.149	0.003615887
main dataset		Guberman C 2013 a	body weight	-0.148	0.578	-1.281	0.985	0.797859549
main dataset		Guberman C 2013 b	body weight	2.569	0.780	1.040	4.097	0.000989455
main dataset		Desai M 2014 b	body weight	2.192	0.633	0.952	3.431	0.000530469
main dataset		Desai M 2014 d	body weight	1.742	0.587	0.591	2.894	0.003006007
main dataset		Desai M 2014 f	body weight	0.540	0.509	-0.457	1.538	0.288421266
main dataset		Desai M 2014 h	body weight	0.197	0.501	-0.786	1.179	0.694623102
main dataset		Desai M 2015	body weight	3.695	0.950	1.834	5.557	0.000100151
main dataset		Walker CD 2008 b	body weight	0.932	0.372	0.202	1.662	0.012295609
main dataset		Naef L 2008	body weight	-0.229	0.487	-1.185	0.726	0.638413195
main dataset		Mendes-da-Silva C 2014	body weight	1.647	0.372	0.919	2.375	0.000009318
main dataset		Khan IY 2003 d	body weight	1.198	0.504	0.211	2.185	0.017394413
main dataset		Khan IY 2005 a	body weight	0.726	0.462	-0.179	1.631	0.116042994
main dataset		Khan IY 2005 b	body weight	0.260	0.449	-0.620	1.140	0.562390736
main dataset		Khan IY 2005 d	body weight	-0.644	0.486	-1.597	0.310	0.185669113
main dataset		Khan IY 2005 e	body weight	-0.346	0.478	-1.283	0.590	0.468754906
main dataset		Khan IY 2004 a	body weight	0.726	0.462	-0.178	1.631	0.115622748
main dataset		Armitage JA 2005 a	body weight	0.934	0.608	-0.257	2.126	0.124398790
main dataset		Armitage JA 2005 b	body weight	0.346	0.610	-0.850	1.541	0.570892190
main dataset		Bautista CJ 2016 a	body weight	0.166	0.634	-1.076	1.407	0.793674692
main dataset		Bautista CJ 2016 b	body weight	0.620	0.647	-0.649	1.888	0.338646196
main dataset		Rodríguez-González GL 2015 a	body weight	1.281	0.648	0.011	2.551	0.048052297
main dataset		Rodríguez-González GL 2015 b	body weight	1.715	0.690	0.363	3.068	0.012947063
main dataset		Zambrano E 2010	body weight	0.253	0.635	-0.991	1.498	0.689971681
main dataset		Santos M 2015	body weight	1.773	0.746	0.310	3.236	0.017539242
main dataset		Page KC 2009	body weight	1.778	0.482	0.833	2.723	0.000226672
main dataset		Howie GJ 2013 a	body weight	1.253	0.399	0.470	2.036	0.001702636
main dataset		Howie GJ 2013 b	body weight	1.324	0.403	0.534	2.115	0.001020580
main dataset		Smith T 2014	body weight	1.499	0.506	0.507	2.491	0.003061565
main dataset		Cordero P 2015 a	body weight	0.718	0.562	-0.384	1.819	0.201492575
main dataset		Cordero P 2015 b	body weight	0.855	0.569	-0.260	1.970	0.132932350
main dataset		Sloboda DM 2009 a	body weight	-0.947	0.362	-1.657	-0.237	0.008958922
main dataset		Sloboda DM 2009 b	body weight	-0.538	0.350	-1.223	0.147	0.123955354
main dataset		Gray C, Reynolds CM 2015	body weight	1.613	0.665	0.310	2.916	0.015222084
main dataset		Pileggi CA 2016	body weight	1.382	0.643	0.122	2.641	0.031512731
main dataset		Song Y 2015	body weight	2.237	0.570	1.119	3.354	0.000087356
main dataset		Latouche C 2014	body weight	0.064	0.646	-1.202	1.329	0.921515270
main dataset		Ghosh P 2001	body weight	0.487	0.454	-0.402	1.377	0.282955088
main dataset		MacPherson RE 2015	body weight	0.133	0.334	-0.521	0.787	0.689341706
	Fixed			0.690	0.071	0.551	0.829	0.000000000
	Random			0.813	0.127	0.564	1.061	0.000000000

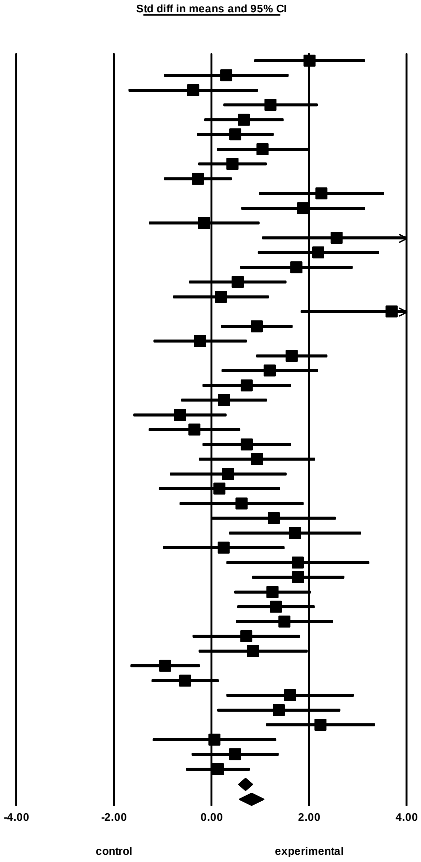

Panel C Forest Plot for Leptin

Subgroup within study	Model	Study name	Outcome	Std diff in means	Standard error	Lower limit	Upper limit	p-Value
main dataset		Tamashiro KL 2009 a	leptin	0.468	0.654	-0.814	1.749	0.474521059
main dataset		Sun B 2013 a	leptin	3.412	0.905	1.639	5.185	0.000162158
main dataset		Sun B 2013 b	leptin	2.478	0.768	0.974	3.982	0.001245114
main dataset		Marco A 2014	leptin	1.269	0.586	0.121	2.418	0.030275332
main dataset		Lecoutre S 2016 a	leptin	1.038	0.377	0.300	1.777	0.005830399
main dataset		Lecoutre S 2016 b	leptin	0.610	0.362	-0.099	1.319	0.091671961
main dataset		Desai M 2014 b	leptin	1.908	0.603	0.726	3.090	0.001561700
main dataset		Desai M 2014 d	leptin	1.593	0.574	0.468	2.717	0.005509621
main dataset		Desai M 2014 f	leptin	1.282	0.549	0.206	2.358	0.019552190
main dataset		Desai M 2014 h	leptin	1.153	0.540	0.094	2.211	0.032786713
main dataset		Walker CD 2008 b	leptin	0.614	0.341	-0.055	1.282	0.071984971
main dataset		Taylor PD 2005	leptin	0.910	0.470	-0.011	1.831	0.052775059
main dataset		Vega CC 2015 a	leptin	1.098	0.536	0.047	2.149	0.040611123
main dataset		Vega CC 2015 b	leptin	1.029	0.532	-0.014	2.072	0.053125325
main dataset		Zambrano E 2010	leptin	2.108	0.789	0.562	3.653	0.007538547
main dataset		Page KC 2009	leptin	0.287	0.410	-0.517	1.091	0.484650418
main dataset		Howie GJ 2009 a	leptin	1.215	0.397	0.436	1.994	0.002232582
main dataset		Howie GJ 2009 b	leptin	1.179	0.396	0.404	1.955	0.002876303
main dataset		Howie GJ 2009 c	leptin	1.020	0.388	0.259	1.781	0.008594956
main dataset		Howie GJ 2009 d	leptin	0.638	0.374	-0.096	1.371	0.088532760
main dataset		Smith T 2014	leptin	1.965	0.545	0.897	3.032	0.000308385
main dataset		Pereira TJ 2015 a	leptin	0.346	0.582	-0.794	1.486	0.551883498
main dataset		Reynolds CM 2015	leptin	2.089	0.718	0.682	3.496	0.003609616
main dataset		Song Y 2015	leptin	1.456	0.503	0.470	2.441	0.003801074
main dataset		Miotto PM 2013 a	leptin	0.410	0.476	-0.524	1.343	0.389913335
main dataset		Miotto PM 2013 b	leptin	-0.117	0.578	-1.250	1.015	0.838889552
	Fixed			1.015	0.096	0.827	1.203	0.000000000
	Random			1.061	0.120	0.826	1.296	0.000000000

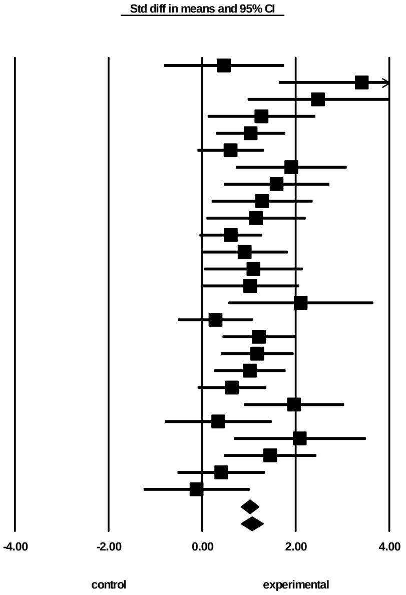

Panel D Forest Plot for Glucose

Subgroup within study	Model	Study name	Outcome	Std diff in means	Standard error	Lower limit	Upper limit	p-Value
main dataset		Srinivasan M 2006 b	glucose	0.819	0.491	-0.143	1.780	0.095309158
main dataset		Tamashiro KL 2009 a	glucose	0.143	0.646	-1.124	1.410	0.824770762
main dataset		White CL, Morrison CD 2009 a	glucose	0.326	0.429	-0.515	1.167	0.447624268
main dataset		Lecoutre S 2016 a	glucose	-0.188	0.354	-0.882	0.506	0.595686184
main dataset		Lecoutre S 2016 b	glucose	-0.842	0.369	-1.565	-0.119	0.022437013
main dataset		Desai M 2014 b	glucose	2.880	0.714	1.481	4.278	0.000054437
main dataset		Desai M 2014 d	glucose	2.155	0.629	0.923	3.387	0.000607813
main dataset		Desai M 2014 f	glucose	3.123	0.745	1.663	4.582	0.000027584
main dataset		Desai M 2014 h	glucose	2.882	0.714	1.483	4.282	0.000053981
main dataset		Desai M 2015	glucose	2.193	0.731	0.761	3.625	0.002680273
main dataset		Khan IY 2003 a	glucose	0.434	0.584	-0.710	1.579	0.457058278
main dataset		Khan IY 2003 b	glucose	0.096	0.578	-1.037	1.228	0.868620133
main dataset		Khan IY 2003 c	glucose	0.824	0.659	-0.468	2.115	0.211216734
main dataset		Khan IY 2003 d	glucose	2.169	0.728	0.743	3.595	0.002869113
main dataset		Khan IY 2005 a	glucose	1.907	0.696	0.542	3.271	0.006173196
main dataset		Khan IY 2005 b	glucose	0.760	0.598	-0.411	1.932	0.203384432
main dataset		Khan IY 2005 d	glucose	0.095	0.578	-1.038	1.227	0.870002790
main dataset		Khan IY 2005 e	glucose	-0.124	0.578	-1.257	1.009	0.829960771
main dataset		Khan IY 2004 a	glucose	1.764	0.630	0.530	2.999	0.005098487
main dataset		Khan IY 2004 b	glucose	0.753	0.576	-0.376	1.881	0.191010223
main dataset		Vega CC 2015 a	glucose	0.057	0.500	-0.923	1.037	0.909403230
main dataset		Vega CC 2015 b	glucose	0.127	0.501	-0.854	1.108	0.799628239
main dataset		Zambrano E 2010	glucose	1.778	0.747	0.314	3.242	0.017319383
main dataset		Page KC 2009	glucose	0.731	0.422	-0.095	1.558	0.082918121
main dataset		Howie GJ 2009 a	glucose	0.133	0.378	-0.608	0.875	0.724556651
main dataset		Howie GJ 2009 b	glucose	-0.256	0.367	-0.975	0.462	0.484339200
main dataset		Howie GJ 2009 c	glucose	0.105	0.378	-0.636	0.846	0.781476186
main dataset		Howie GJ 2009 d	glucose	-0.513	0.371	-1.240	0.215	0.167006397
main dataset		Smith T 2014	glucose	0.297	0.450	-0.584	1.178	0.509111386
main dataset		Pereira TJ 2015 a	glucose	-0.204	0.579	-1.339	0.930	0.724415932
main dataset		Pereira TJ 2015 b	glucose	-0.368	0.582	-1.509	0.773	0.527261311
main dataset		Latouche C 2014	glucose	0.409	0.652	-0.869	1.686	0.530919348
main dataset		Yang KF 2012	glucose	0.713	0.461	-0.191	1.617	0.122188118
	Fixed			0.419	0.089	0.244	0.593	0.000002625
	Random			0.644	0.164	0.322	0.965	0.000088224

Std diff in means and 95% CI

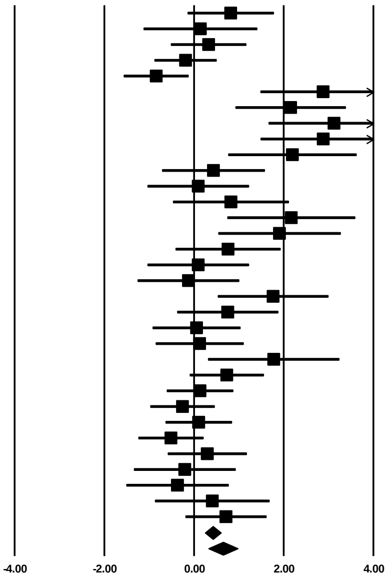

Panel E Forest Plot for Insulin

Subgroup within study	Model	Study name	Outcome	Std diff in means	Standard error	Lower limit	Upper limit	p-Value
main dataset		Srinivasan M 2006 a	insulin	2.740	0.594	1.576	3.903	0.000003930
main dataset		Srinivasan M 2006 b	insulin	2.591	0.639	1.338	3.844	0.000050586
main dataset		Tamashiro KL 2009 a	insulin	1.041	0.686	-0.304	2.385	0.129377034
main dataset		Lecoutre S 2016 a	insulin	0.724	0.365	0.009	1.439	0.047225013
main dataset		Lecoutre S 2016 b	insulin	-0.037	0.354	-0.730	0.656	0.917011254
main dataset		Ambrosetti V 2016 a	insulin	1.401	0.573	0.278	2.523	0.014441305
main dataset		Desai M 2014 b	insulin	5.779	1.137	3.550	8.008	0.000000376
main dataset		Desai M 2014 d	insulin	8.273	1.546	5.244	11.302	0.000000087
main dataset		Desai M 2014 f	insulin	3.625	0.813	2.032	5.218	0.000008200
main dataset		Desai M 2014 h	insulin	1.112	0.537	0.059	2.165	0.038469245
main dataset		Desai M 2015	insulin	7.081	1.556	4.030	10.132	0.000005378
main dataset		Walker CD 2008 a	insulin	-1.429	0.373	-2.160	-0.697	0.000130528
main dataset		Khan IY 2003 a	insulin	-0.559	0.589	-1.712	0.595	0.342347219
main dataset		Khan IY 2003 b	insulin	0.634	0.592	-0.525	1.794	0.283636773
main dataset		Khan IY 2003 c	insulin	0.692	0.651	-0.584	1.968	0.287819144
main dataset		Khan IY 2003 d	insulin	0.635	0.592	-0.525	1.795	0.283262794
main dataset		Khan IY 2005 c	insulin	1.515	0.463	0.608	2.423	0.001068152
main dataset		Khan IY 2005 f	insulin	1.484	0.483	0.537	2.431	0.002126362
main dataset		Khan IY 2004 a	insulin	0.896	0.561	-0.203	1.995	0.110137161
main dataset		Khan IY 2004 b	insulin	1.265	0.609	0.071	2.459	0.037773471
main dataset		Vega CC 2015 a	insulin	0.790	0.519	-0.228	1.807	0.128203483
main dataset		Vega CC 2015 b	insulin	1.147	0.540	0.090	2.205	0.033481818
main dataset		Zambrano E 2010	insulin	2.055	0.782	0.523	3.588	0.008563162
main dataset		Page KC 2009	insulin	0.165	0.409	-0.636	0.967	0.686167850
main dataset		Howie GJ 2009 a	insulin	1.446	0.410	0.643	2.250	0.000420840
main dataset		Howie GJ 2009 b	insulin	1.475	0.412	0.668	2.282	0.000342344
main dataset		Howie GJ 2009 c	insulin	0.930	0.384	0.176	1.683	0.015566555
main dataset		Howie GJ 2009 d	insulin	0.983	0.387	0.225	1.741	0.010989182
main dataset		Smith T 2014	insulin	1.858	0.535	0.809	2.907	0.000515571
main dataset		Pereira TJ 2015 a	insulin	4.398	1.067	2.306	6.490	0.000037830
main dataset		Pereira TJ 2015 b	insulin	0.609	0.591	-0.549	1.766	0.302797972
main dataset		Latouche C 2014	insulin	1.324	0.710	-0.068	2.715	0.062343217
main dataset		Yang KF 2012	insulin	0.131	0.448	-0.747	1.008	0.770518603
	Fixed			0.967	0.091	0.788	1.146	0.000000000
	Random			1.369	0.218	0.941	1.797	0.000000000

Std diff in means and 95% CI

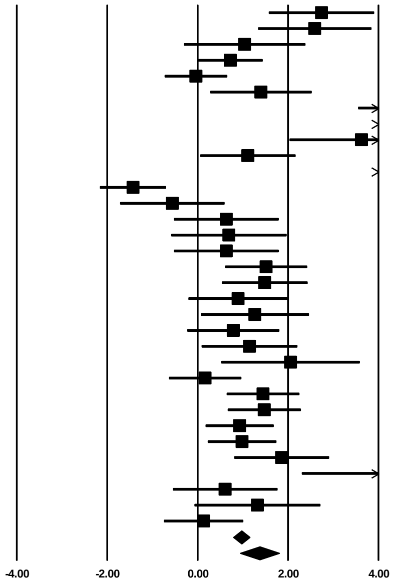

Panel F Forest Plot for HDL-c

Subgroup within study	Model	Study name	Outcome	Std diff in means	Standard error	Lower limit	Upper limit	p-Value
				Statistics for each study				
main dataset		Khan IY 2003 a	HDL-c	-0.778	0.599	-1.951	0.396	0.194070132
main dataset		Khan IY 2003 b	HDL-c	-2.083	0.717	-3.489	-0.678	0.003668155
main dataset		Khan IY 2003 c	HDL-c	-0.444	0.640	-1.699	0.810	0.487550438
main dataset		Khan IY 2003 d	HDL-c	-2.500	0.771	-4.010	-0.990	0.001176866
main dataset		Khan IY 2005 a	HDL-c	0.672	0.593	-0.491	1.836	0.257184281
main dataset		Khan IY 2005 b	HDL-c	-0.139	0.578	-1.271	0.994	0.810582788
main dataset		Khan IY 2005 d	HDL-c	0.500	0.586	-0.649	1.649	0.393768635
main dataset		Khan IY 2005 e	HDL-c	0.430	0.584	-0.715	1.574	0.462023786
main dataset		Khan IY 2004 a	HDL-c	0.240	0.536	-0.812	1.291	0.655181166
main dataset		Khan IY 2004 b	HDL-c	-0.236	0.558	-1.330	0.858	0.672364534
main dataset		Smith T 2014	HDL-c	1.478	0.505	0.489	2.468	0.003391223
main dataset		Gray C, Reynolds CM 2015	HDL-c	0.000	0.577	-1.132	1.132	1.000000000
main dataset		Reynolds CM 2015	HDL-c	0.833	0.602	-0.346	2.013	0.166195169
main dataset		Ghosh P 2001	HDL-c	-1.666	0.519	-2.684	-0.649	0.001325413
	Fixed			-0.131	0.157	-0.438	0.177	0.404680152
	Random			-0.214	0.291	-0.784	0.357	0.463022236

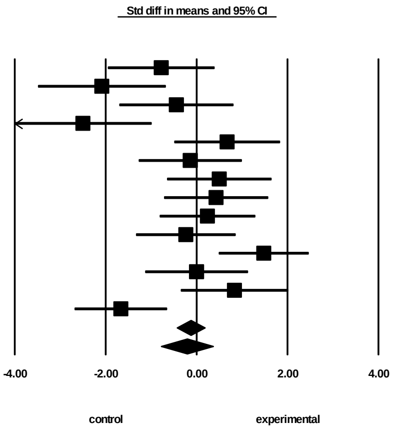

Panel G Forest Plot for Triglycerides

Subgroup within study	Model	Study name	Outcome	Std diff in means	Standard error	Lower limit	Upper limit	p-Value
				Statistics for each study				
main dataset		Srinivasan M 2006 b	triglycerides	1.699	0.550	0.621	2.777	0.002004998
main dataset		Lecoutre S 2016 a	triglycerides	0.269	0.355	-0.427	0.966	0.448054093
main dataset		Lecoutre S 2016 b	triglycerides	-0.260	0.355	-0.956	0.436	0.463661525
main dataset		Seet EL 2015	triglycerides	1.358	0.640	0.103	2.613	0.033964220
main dataset		Desai M 2014 b	triglycerides	1.224	0.545	0.156	2.291	0.024699935
main dataset		Desai M 2014 d	triglycerides	3.046	0.735	1.606	4.486	0.000033938
main dataset		Desai M 2014 f	triglycerides	1.219	0.544	0.152	2.286	0.025202720
main dataset		Desai M 2014 h	triglycerides	1.461	0.563	0.358	2.565	0.009411711
main dataset		Desai M 2015	triglycerides	1.641	0.668	0.333	2.950	0.013937027
main dataset		Khan IY 2003 a	triglycerides	-0.161	0.578	-1.295	0.972	0.780395467
main dataset		Khan IY 2003 b	triglycerides	-0.417	0.584	-1.560	0.727	0.475238177
main dataset		Khan IY 2003 c	triglycerides	1.035	0.673	-0.285	2.355	0.124330790
main dataset		Khan IY 2003 d	triglycerides	1.287	0.634	0.044	2.530	0.042485542
main dataset		Khan IY 2005 a	triglycerides	0.776	0.599	-0.398	1.949	0.195175331
main dataset		Khan IY 2005 b	triglycerides	0.167	0.578	-0.967	1.300	0.773212583
main dataset		Khan IY 2005 d	triglycerides	0.325	0.581	-0.814	1.464	0.576440317
main dataset		Khan IY 2005 e	triglycerides	0.405	0.583	-0.738	1.548	0.487683465
main dataset		Khan IY 2004 a	triglycerides	-0.769	0.554	-1.855	0.316	0.164936375
main dataset		Khan IY 2004 b	triglycerides	0.195	0.558	-0.898	1.288	0.726269396
main dataset		Vega CC 2015 a	triglycerides	3.327	0.772	1.814	4.840	0.000016332
main dataset		Vega CC 2015 b	triglycerides	0.749	0.517	-0.265	1.763	0.147462244
main dataset		Zhang X 2011	triglycerides	1.381	0.511	0.379	2.383	0.006905082
main dataset		Gray C, Reynolds CM 2015	triglycerides	1.248	0.631	0.011	2.484	0.048034033
main dataset		Reynolds CM 2015	triglycerides	1.262	0.632	0.023	2.501	0.045900565
main dataset		Yang KF 2012	triglycerides	0.085	0.447	-0.792	0.962	0.848942767
main dataset		Ghosh P 2001	triglycerides	0.993	0.474	0.064	1.921	0.036248754
	Fixed			0.712	0.107	0.503	0.921	0.000000000
	Random			0.828	0.172	0.491	1.165	0.000001444

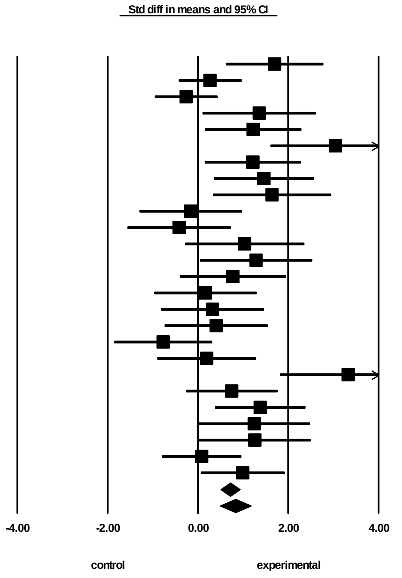

Panel H Forest Plot for Systolic Blood Pressure

Subgroup within study	Model	Study name	Outcome	Statistics for each study: Std diff in means	Standard error	Lower limit	Upper limit	p-Value
main dataset		Guberman C 2013 a	SBP	1.354	0.640	0.099	2.608	0.034442250
main dataset		Guberman C 2013 b	SBP	1.526	0.656	0.240	2.812	0.020022603
main dataset		Desai M 2014 a	SBP	1.321	0.552	0.239	2.402	0.016681515
main dataset		Desai M 2014 c	SBP	1.728	0.586	0.580	2.877	0.003181084
main dataset		Desai M 2014 e	SBP	1.181	0.542	0.119	2.244	0.029229088
main dataset		Desai M 2014 g	SBP	2.384	0.654	1.103	3.666	0.000266318
main dataset		Khan IY 2003 c	SBP	1.414	0.707	0.028	2.799	0.045574105
main dataset		Khan IY 2003 d	SBP	1.464	0.650	0.190	2.738	0.024353050
main dataset		Khan IY 2005 b	SBP	1.491	0.604	0.306	2.675	0.013623071
main dataset		Khan IY 2005 e	SBP	1.513	0.631	0.277	2.748	0.016432339
main dataset		Khan IY 2004 b	SBP	1.491	0.604	0.306	2.675	0.013623071
main dataset		Gray C, Reynolds CM 2015	SBP	1.571	0.660	0.277	2.866	0.017367718
	Fixed			1.521	0.179	1.171	1.871	0.000000000
	Random			1.521	0.179	1.171	1.871	0.000000000

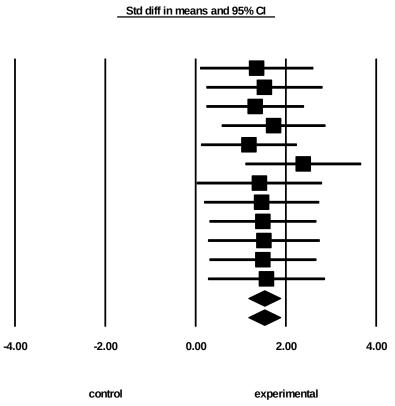

Supplementary Figure S4: Subgroup analyses for HDL-c, extended dataset.

Horizontal lines represent the 95%CIs for the data. The summarized effects (D) are considered statistically significant when their 95%CIs do not cross zero. We used a random-effect model (filled circles) whether heterogeneity was observed, while the fixed-effect model was applied in the absence of heterogeneity (filled squares). Included moderators for the main data set are: strain (Sprague Dawley, Wistar), sex (male, female), offspring age at testing (young adult), maternal age (young adult), intervention timing [perinatal (gestation and lactation), restricted to gestation period], maternal body weight (increased, not increased), birth weight (decreased, increased, not different), and diet type (chow-based, commercial). Abbreviations: n: number of data points, SD: Sprague Dawley, YA: Young Age, GES: gestation period only, GES+LAC: gestation and suckling periods, BW: body weight, NS: not significant.

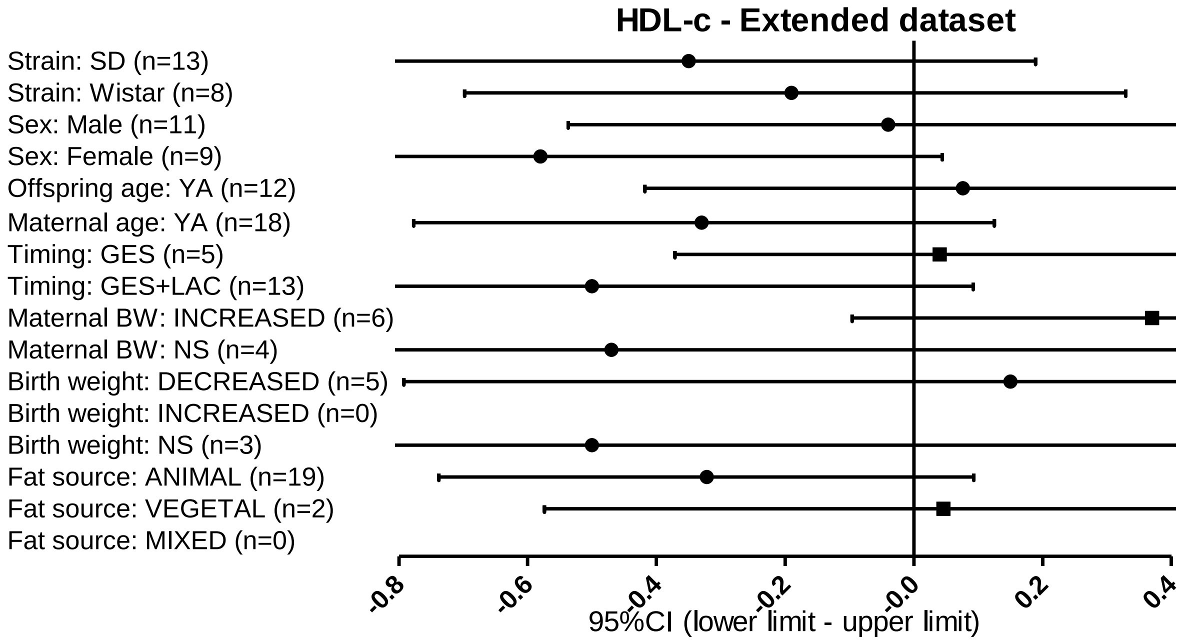

Supplementary Figure S5: Subgroup analyses for main dataset.

Horizontal lines represent the 95%CIs for the data. The summarized effects (D) are considered statistically significant when their 95%CIs do not cross zero. We used a random-effect model (filled circles) whether heterogeneity was observed, while the fixed-effect model was applied in the absence of heterogeneity (filled squares). Included moderators for the main data set are: strain (Sprague Dawley, Wistar), sex (male, female), offspring age at testing (young adult), maternal age (young adult), intervention timing [perinatal (gestation and lactation), restricted to gestation period], maternal body weight (increased, not increased), birth weight (decreased, increased, not different), and diet type (chow-based, commercial). Subgroup analysis of subsets where heterogeneity was not significant was not performed (leptin and SBP subsets). Abbreviations: n: number of data points, SD: Sprague Dawley, YA: Young Age, GES: gestation period only, GES+LAC: gestation and suckling periods, BW: body weight, NS: not significant.

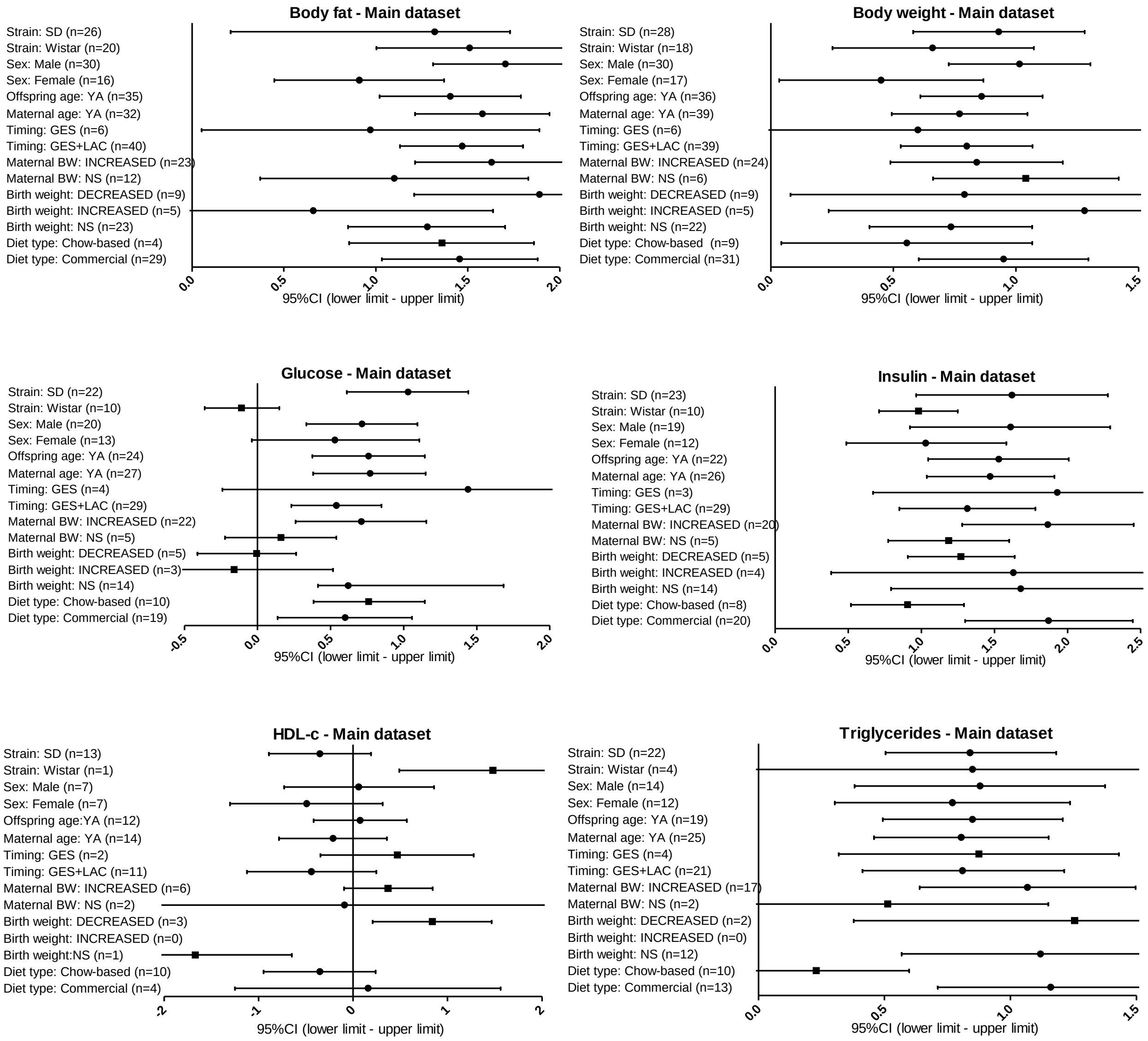

Body fat - Main dataset
Strain: SD (n=26)
Strain: Wistar (n=20)
Sex: Male (n=30)
Sex: Female (n=16)
Offspring age: YA (n=35)
Maternal age: YA (n=32)
Timing: GES (n=6)
Timing: GES+LAC (n=40)
Maternal BW: INCREASED (n=23)
Maternal BW: NS (n=12)
Birth weight: DECREASED (n=9)
Birth weight: INCREASED (n=5)
Birth weight: NS (n=23)
Diet type: Chow-based (n=4)
Diet type: Commercial (n=29)
0.0
0.5
1.0
1.5
2.0
95%CI (lower limit - upper limit)
Body weight - Main dataset
Strain: SD (n=28)
Strain: Wistar (n=18)
Sex: Male (n=30)
Sex: Female (n=17)
Offspring age: YA (n=36)
Maternal age: YA (n=39)
Timing: GES (n=6)
Timing: GES+LAC (n=39)
Maternal BW: INCREASED (n=24)
Maternal BW: NS (n=6)
Birth weight: DECREASED (n=9)
Birth weight: INCREASED (n=5)
Birth weight: NS (n=22)
Diet type: Chow-based (n=9)
Diet type: Commercial (n=31)
0.0
0.5
1.0
1.5
95%CI (lower limit - upper limit)
Glucose - Main dataset
Strain: SD (n=22)
Strain: Wistar (n=10)
Sex: Male (n=20)
Sex: Female (n=13)
Offspring age: YA (n=24)
Maternal age: YA (n=27)
Timing: GES (n=4)
Timing: GES+LAC (n=29)
Maternal BW: INCREASED (n=22)
Maternal BW: NS (n=5)
Birth weight: DECREASED (n=5)
Birth weight: INCREASED (n=3)
Birth weight: NS (n=14)
Diet type: Chow-based (n=10)
Diet type: Commercial (n=19)
-0.5
0.0
0.5
1.0
1.5
2.0
95%CI (lower limit - upper limit)
Insulin - Main dataset
Strain: SD (n=23)
Strain: Wistar (n=10)
Sex: Male (n=19)
Sex: Female (n=12)
Offspring age: YA (n=22)
Maternal age: YA (n=26)
Timing: GES (n=3)
Timing: GES+LAC (n=29)
Maternal BW: INCREASED (n=20)
Maternal BW: NS (n=5)
Birth weight: DECREASED (n=5)
Birth weight: INCREASED (n=4)
Birth weight: NS (n=14)
Diet type: Chow-based (n=8)
Diet type: Commercial (n=20)
0.0
0.5
1.0
1.5
2.0
2.5
95%CI (lower limit - upper limit)
HDL-c - Main dataset
Strain: SD (n=13)
Strain: Wistar (n=1)
Sex: Male (n=7)
Sex: Female (n=7)
Offspring age:YA (n=12)
Maternal age: YA (n=14)
Timing: GES (n=2)
Timing: GES+LAC (n=11)
Maternal BW: INCREASED (n=6)
Maternal BW: NS (n=2)
Birth weight: DECREASED (n=3)
Birth weight: INCREASED (n=0)
Birth weight:NS (n=1)
Diet type: Chow-based (n=10)
Diet type: Commercial (n=4)
-2
-1
0
1
2
95%CI (lower limit - upper limit)
Triglycerides - Main dataset
Strain: SD (n=22)
Strain: Wistar (n=4)
Sex: Male (n=14)
Sex: Female (n=12)
Offspring age: YA (n=19)
Maternal age: YA (n=25)
Timing: GES (n=4)
Timing: GES+LAC (n=21)
Maternal BW: INCREASED (n=17)
Maternal BW: NS (n=2)
Birth weight: DECREASED (n=2)
Birth weight: INCREASED (n=0)
Birth weight: NS (n=12)
Diet type: Chow-based (n=10)
Diet type: Commercial (n=13)
0.0
0.5
1.0
1.5
95%CI (lower limit - upper limit)

Supplementary Figure S6: Meta-regression uncovering the potential influence of moderators.

Bubble plots showing the relationship (fitted regression lines) of moderator on changes in outcomes. The bubbles represent the estimates from each study; positive slopes indicate increased effect sizes with increasing levels of moderator. We examined the potential influence of: 1) offspring age at measurement (offspring_age) in young adults, 2) maternal age at mating/conception (age_mating) in young adult dams, 3) dietary intervention start (diet_duration), 4) litter size (litter_size), 5) increase in energy from fat in experimental diet with respect to control diet (fat_exp-con_kcal), and 6) protein-to-non protein ratio in experimental diet (P:NP). We additionally ran a meta-regression with gender as predictor variable to estimate the impact of sex on effect size (values of 1 for males and 2 for females were arbitrarily assigned). Data are presented only for significant slope p-values separately for each dataset and for each outcome. If visual inspection of the effect plots revealed that the result was driven by a single outlying data point or by two not independent data points from one same study, analysis was repeated after removing those data points.

Body fat – extended dataset

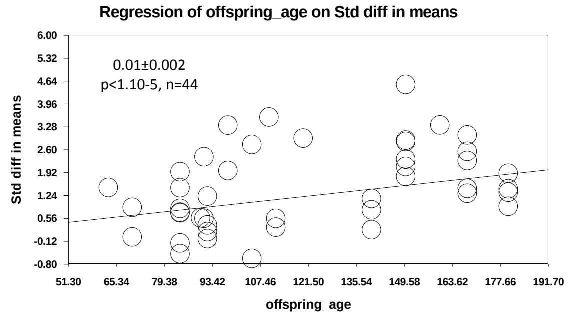

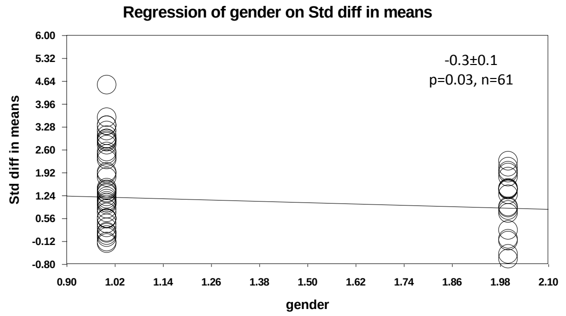

Body fat– main dataset

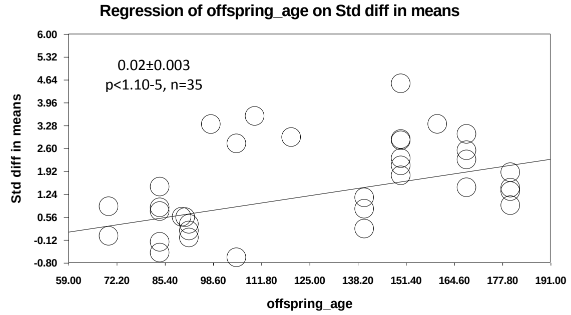

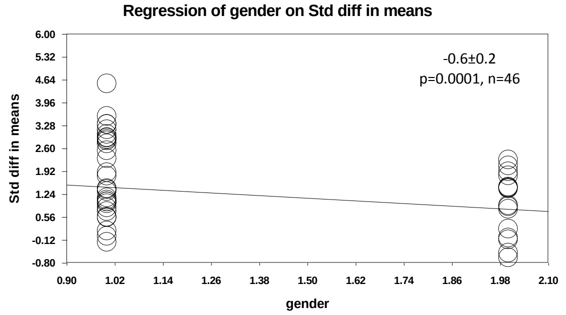

Body weight – extended dataset

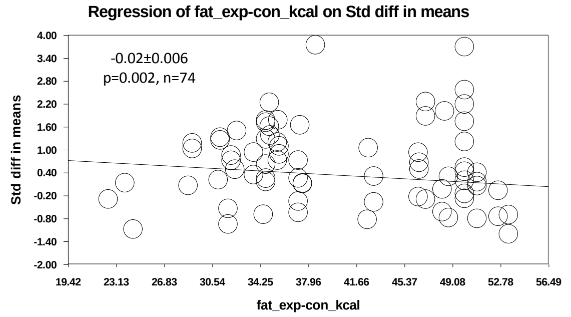

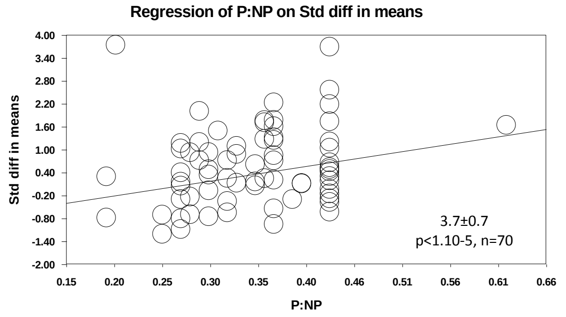

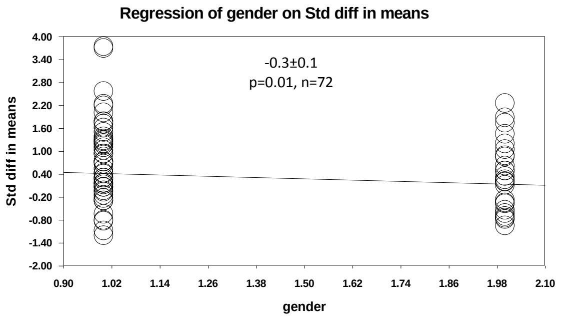

Body weight – main dataset

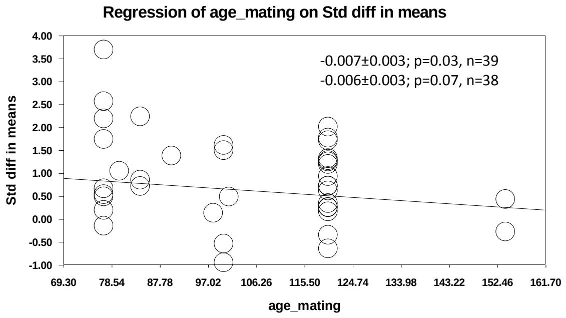

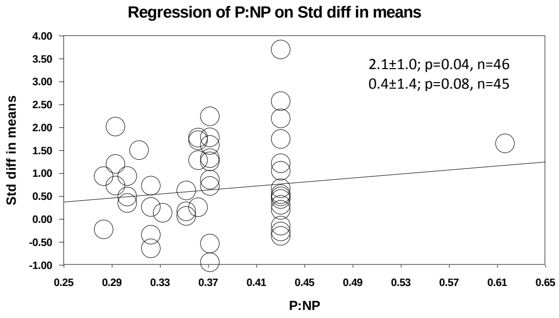

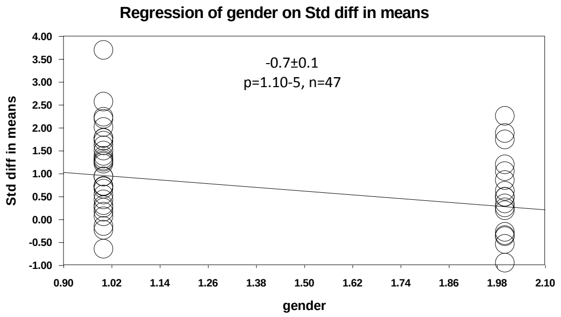

Leptin – extended dataset

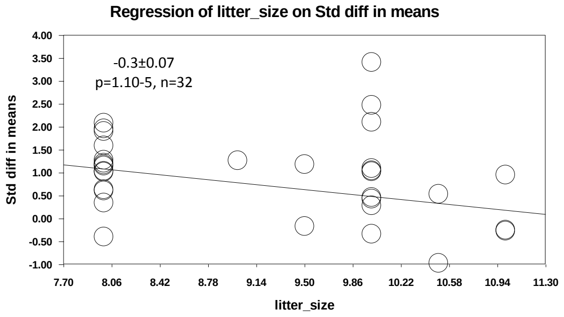

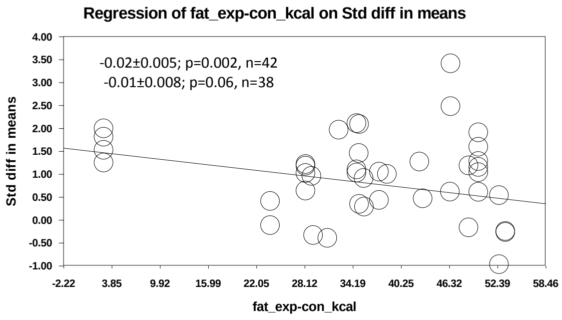

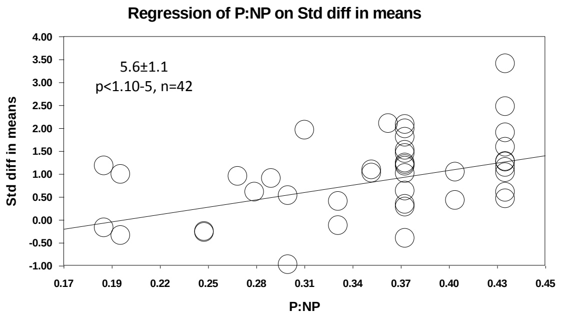

Leptin – main dataset

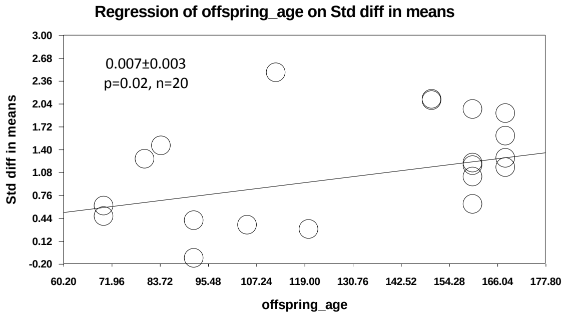

Glucose – extended dataset

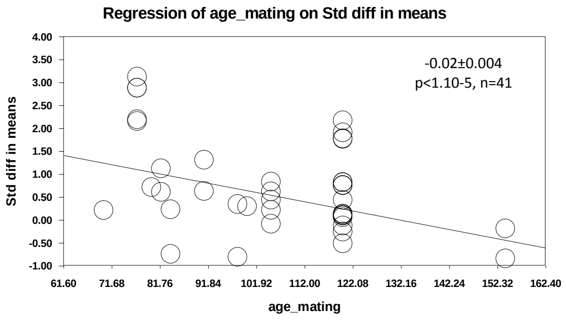

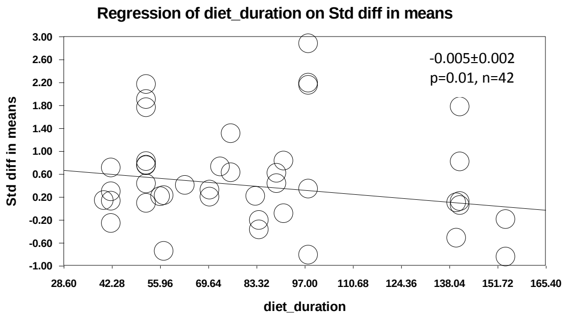

Glucose – main dataset

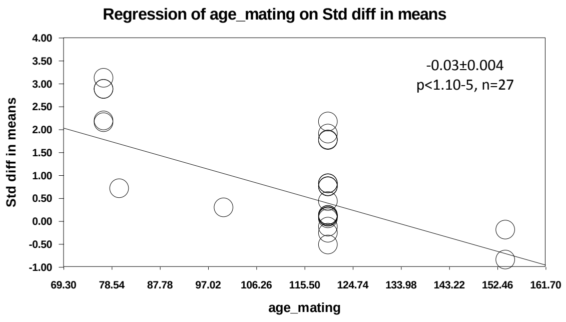

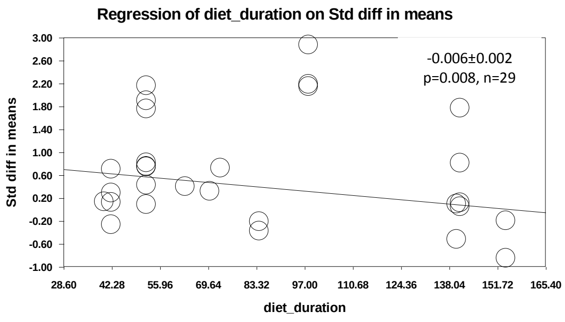

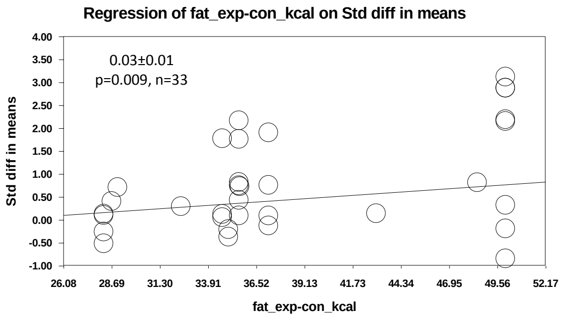

Insulin – extended dataset

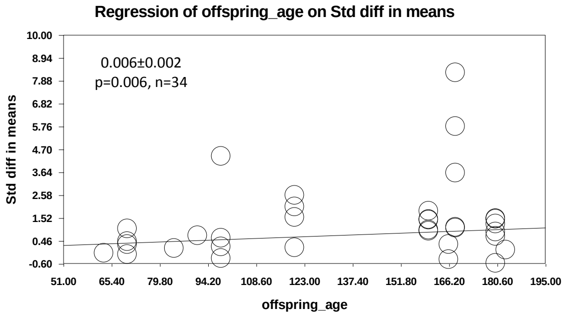

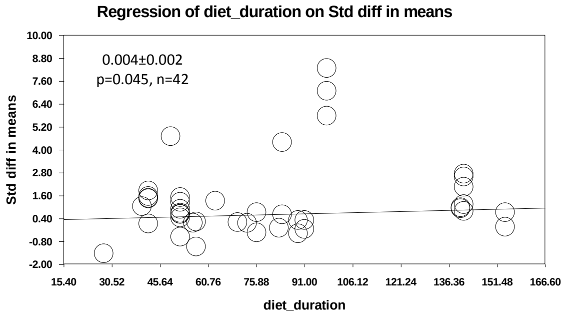

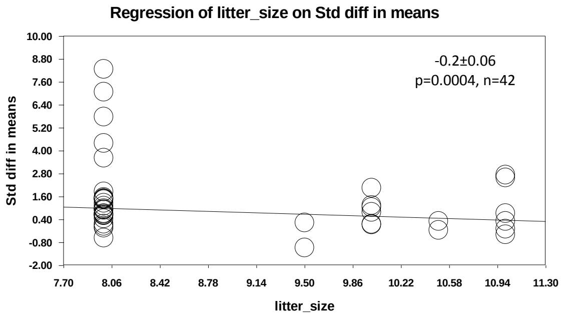

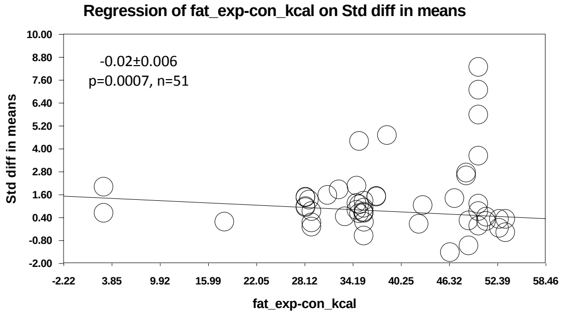

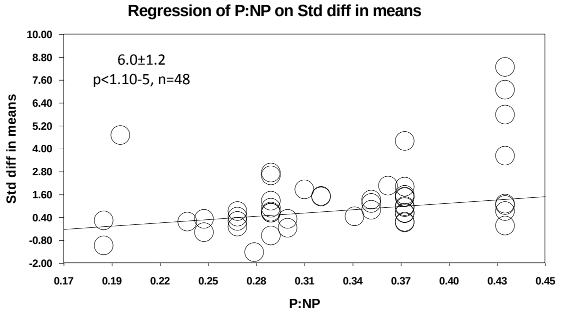

Insulin – main dataset

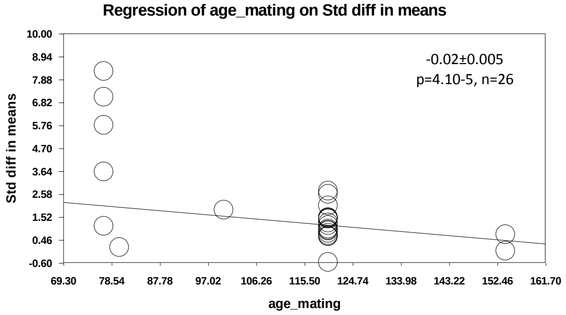

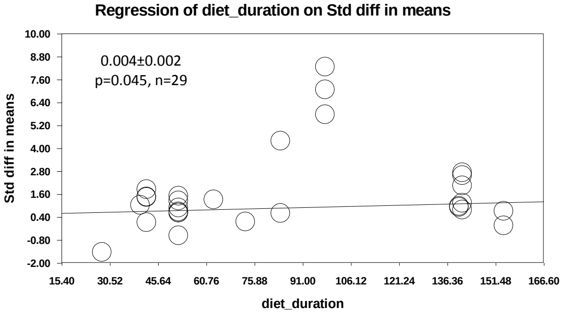

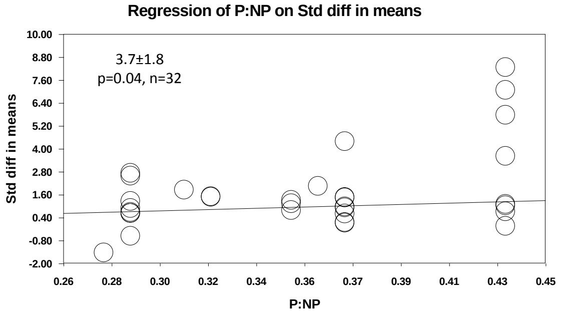

HDL cholesterol – extended dataset

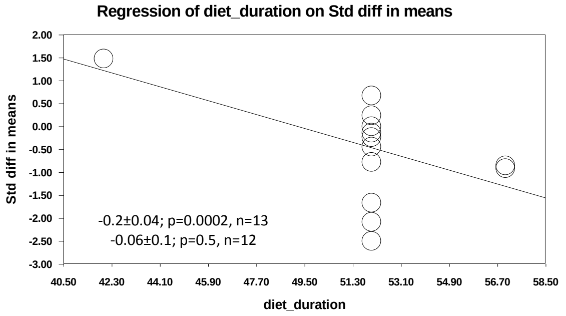

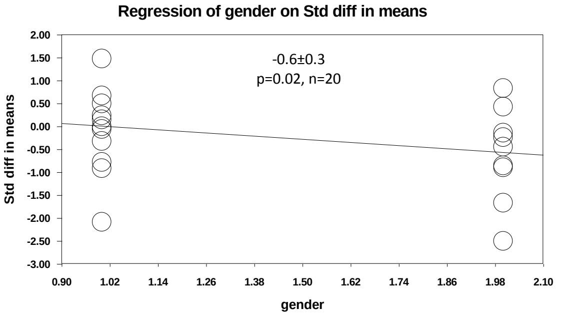

HDL cholesterol – main dataset

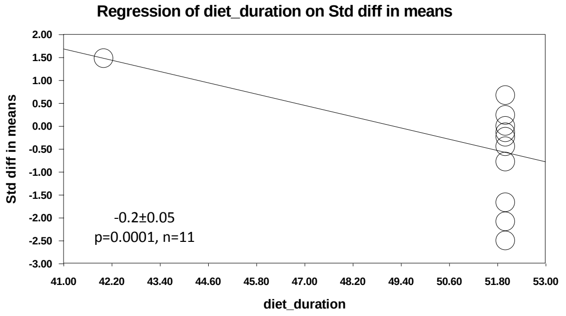

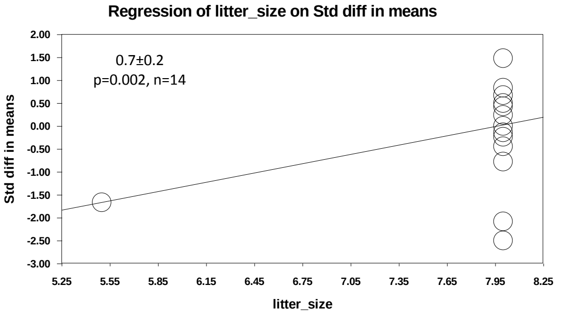

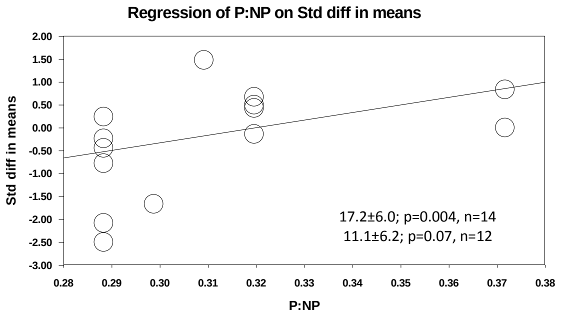

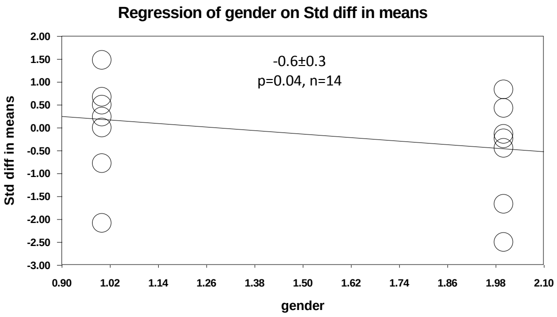

Triglycerides – extended dataset

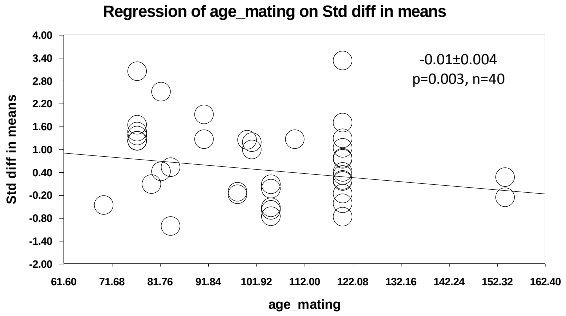

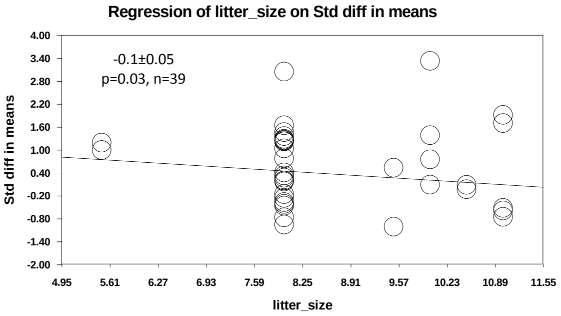

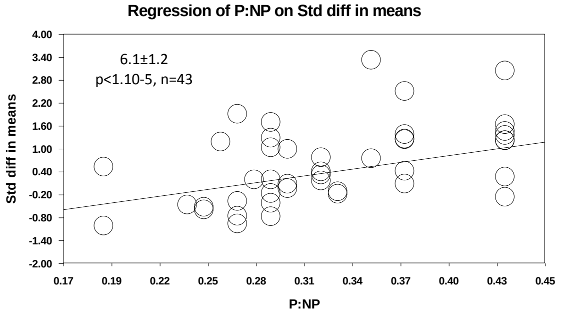

Triglycerides – main dataset

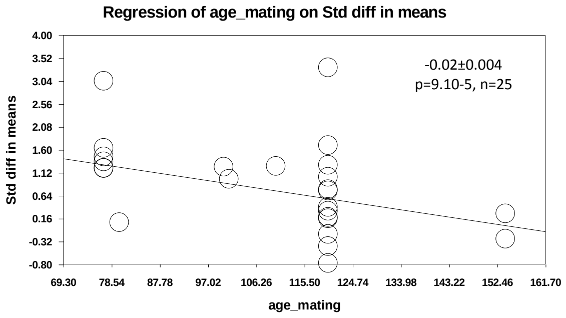

Systolic Blood Pressure – extended dataset

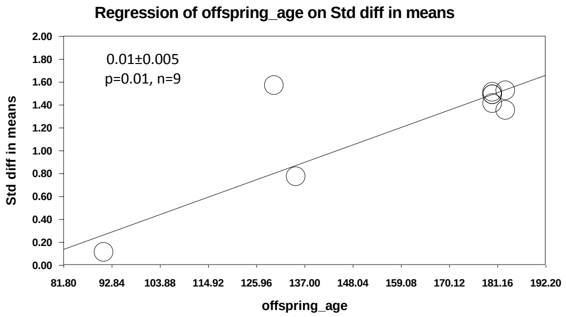
Regression of offspring_age on Std diff in means
0.01±0.005
p=0.01, n=9
Std diff in means
0.00
0.20
0.40
0.60
0.80
1.00
1.20
1.40
1.60
1.80
2.00
81.80
92.84
103.88
114.92
125.96
137.00
148.04
159.08
170.12
181.16
192.20
offspring_age

Supplementary Figure S7: Funnel plots for the presence of data distribution asymmetry and Egger's test for asymmetry in extended and main datasets.

Data are presented separately for each outcome.

Body fat – extended (intercept±ES=4.3±0.7, p-value <0.001) and main (intercept±ES=4.5±0.8, p-value <0.001) datasets.

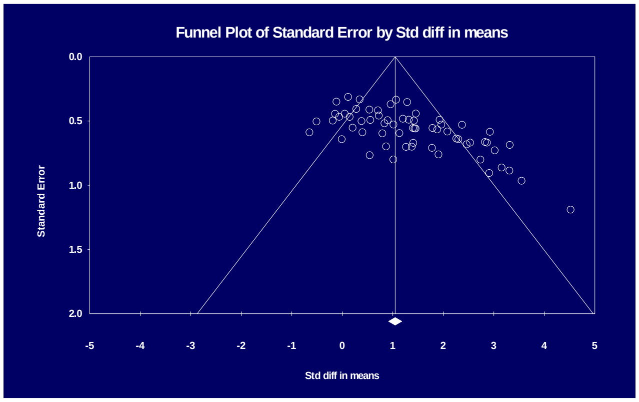

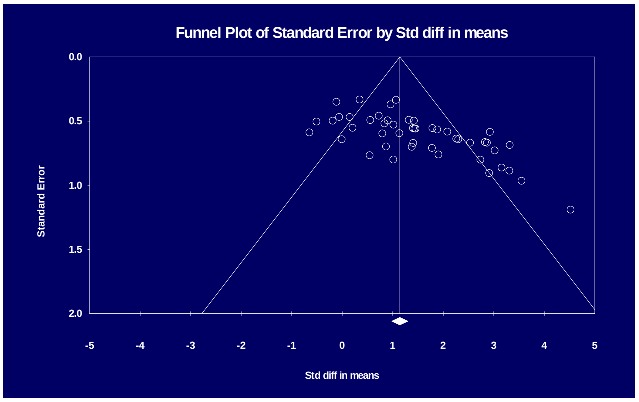

Body weight – extended (intercept±ES= 4.0±0.7, p-value<0.001) and main (intercept±ES= 3.4±1.0, p-value=0.001) datasets.

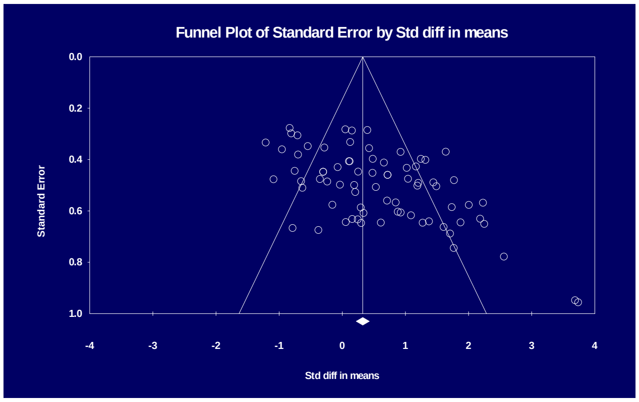

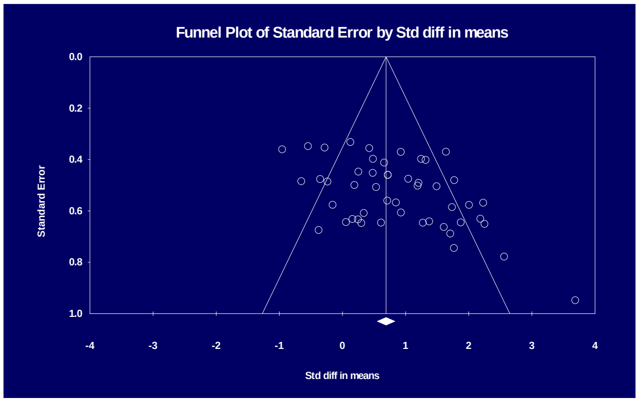

Leptin – extended (intercept±ES= 3.3±0.9, p-value=0.001) and main (intercept±ES= 2.9±0.8, p-value=0.002) datasets.

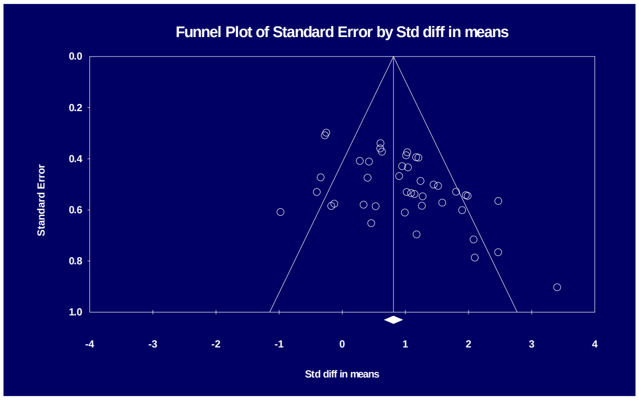

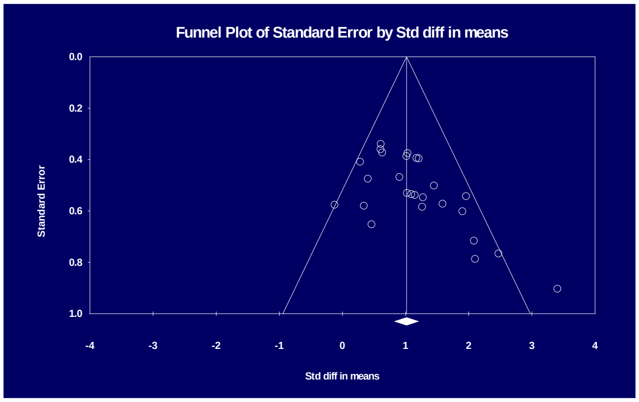

Glucose – – extended (intercept±ES= 3.4±0.8, p-value <0.001) and main (intercept±ES= 5.4±0.9, p-value <0.001) datasets.

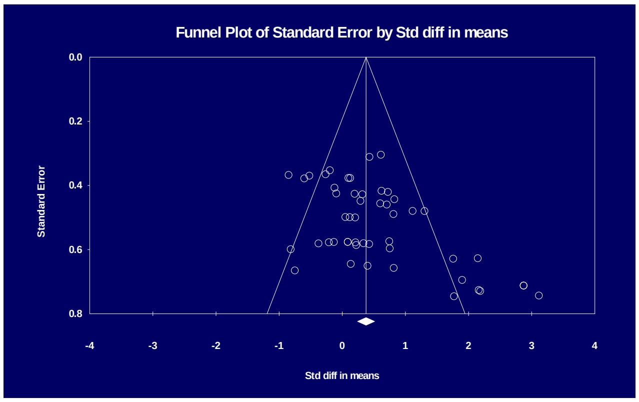

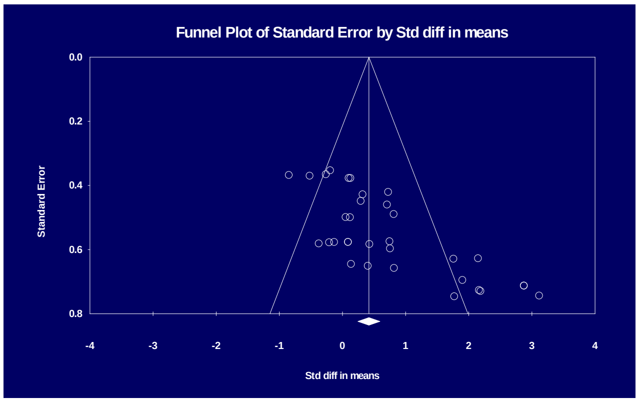

Insulin – extended (intercept±ES= 5.0±0.8, p-value <0.001) and main (intercept±ES= 5.3±0.9, p-value <0.001) datasets.

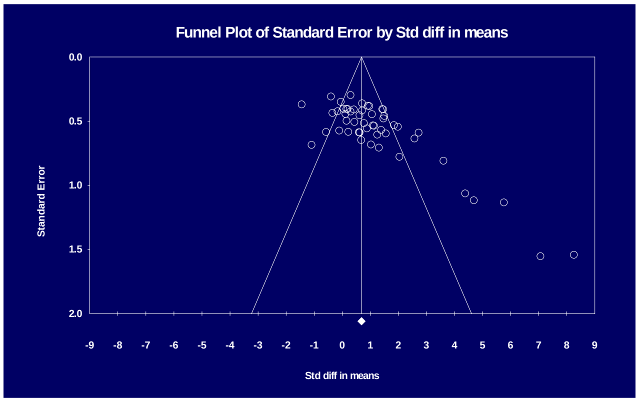

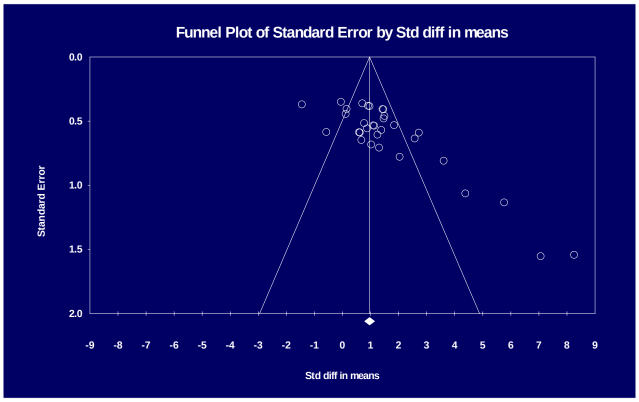

HDL-c – extended (intercept±ES= -2.4±2.0, p-value =0.3) and main (intercept±ES= -9.4±4.1, p-value =0.04) datasets.

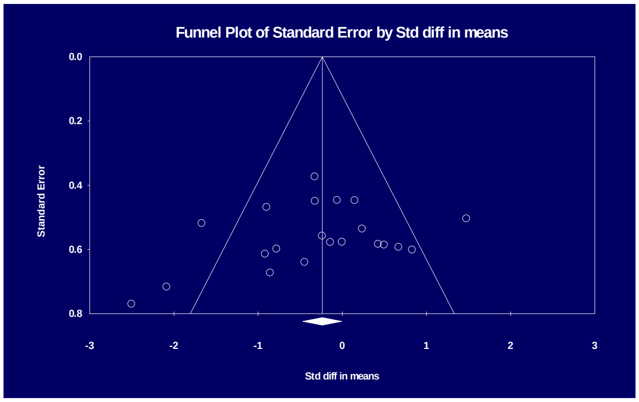

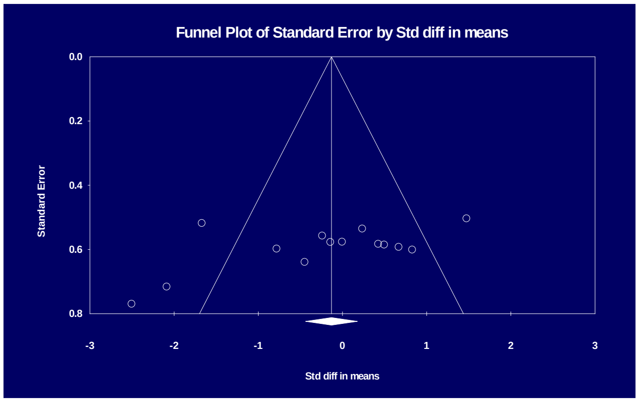

Triglycerides – extended (intercept±ES= 4.4±1.0, p-value <0.001) and main (intercept±ES= 4.7±1.3, p-value =0.002) datasets.

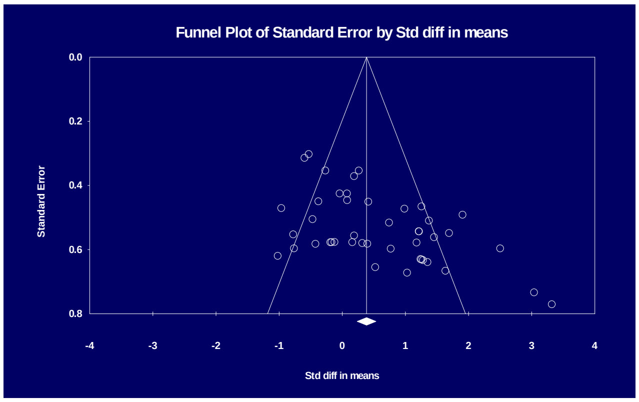

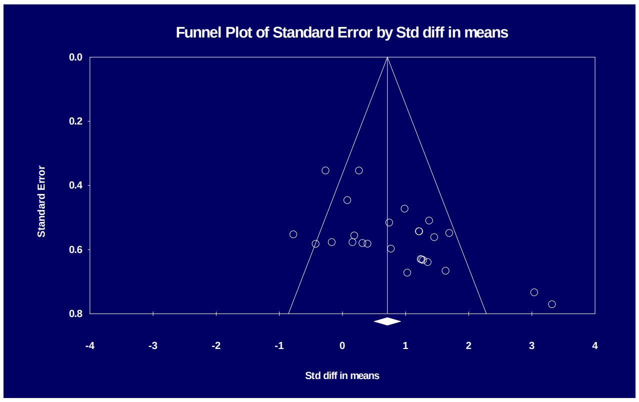

Systolic Blood Pressure – extended (intercept±ES= 5.6±1.0, p-value <0.001) and main (intercept±ES= 2.3±1.8, p-value =0.2) datasets.

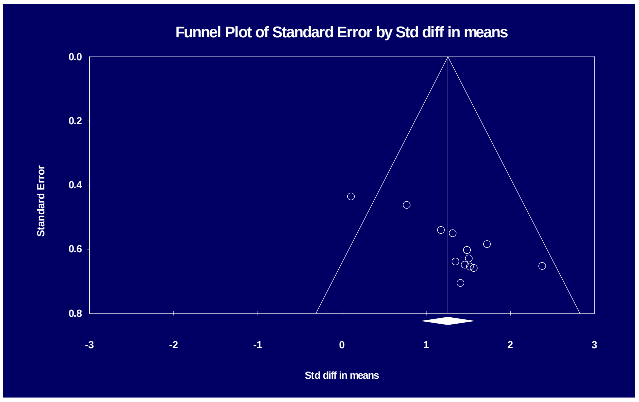

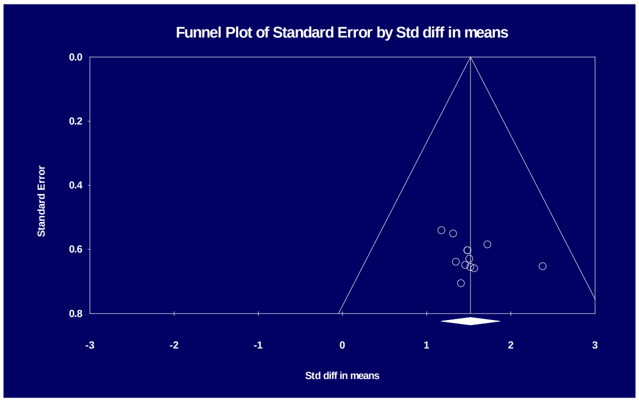

Supplement: Supplementary file 1 — Supplementary Information [file 41598_2017_5344_MOESM1_ESM.pdf]
